# Mechanosensitive phosphorylation of *NFATC4* at S213/S217 drives fibroblast-to-myofibroblast transition and fibrosis

**DOI:** 10.1172/JCI195121

**Published:** 2026-09-01

**Authors:** Safwen Kadri, Laura F. Mattner, Zhen Zeng, Sai Rama Sridatta Prakki, Arun Kumar Verma, Umut Cetin, Christoph H. Mayr, Meshal Ansari, Xin Wei, Sara Asgharpour, Anita A. Wasik, Nikolaus Kneidinger, Mircea-Gabriel Stoleriu, Jürgen Behr, Julien Polleux, Ali Önder Yildirim, Laurens J. De Sadeleer, Wim A. Wuyts, Gerald Burgstaller, Matthias Mann, Martin Mück-Häusl, Herbert B. Schiller

**Affiliations:** 1 Research Unit for Precision Regenerative Medicine, Helmholtz Munich, Comprehensive Pneumology Center, Member of the German Center for Lung Research (DZL), Munich, Germany.; 2 Institute of Computational Biology, Helmholtz Zentrum München, Munich, Germany.; 3 Institute of Lung Health and Immunity, Helmholtz Munich, Comprehensive Pneumology Center, Member of the German Center for Lung Research (DZL), Munich, Germany.; 4 Department of Internal Medicine V, Ludwig-Maximilians University Munich, Member of the German Center for Lung Research (DZL), CPC-M bioArchive, Munich, Germany.; 5 Comprehensive Pneumology Center with the CPC-M bioArchive, Member of the German Center of Lung Research (DZL), Munich, Germany.; 6 Research & Innovation Unit, Department of Ophthalmic Optics, Health University of Applied Sciences Tyrol, Innsbruck, Austria.; 7 Institute of Experimental Pneumology, LMU University Hospital, Ludwig-Maximilians University Munich, Member of the German Center for Lung Research (DZL), Munich, Germany.; 8 Laboratory of Respiratory Diseases and Thoracic Surgery (BREATHE), Department of Chronic Diseases and Metabolism, KU Leuven, Leuven, Belgium.; 9 Max Planck Institute of Biochemistry, Department of Proteomics and Signal Transduction, Martinsried, Germany

**Keywords:** Cell biology, Pulmonology, Fibrosis, Proteomics, Transcription

## Abstract

Mechanosensitive feedback between tissue stiffness and cellular contractile forces instructs cell identity. To characterize phosphorylation-mediated mechanosensing, we charted the global phosphoproteome dynamics of primary human lung fibroblasts on fibronectin-coated polydimethylsiloxane substrates of defined stiffness. We identified a key signaling threshold at 2–8 kPa, above which cells activated cytoskeletal remodeling, ECM secretion, and transition to a *CTHRC1*^+^/*ACTA2*^+^ myofibroblast state, accompanied by stiffness-dependent phosphorylation of the transcription factor *NFATC4* at S213/S217. In micro-CT staged pulmonary fibrosis tissues, *NFATC4* expression increased progressively, colocalizing with *CTHRC1* and *ACTA2* in myofibroblasts. Transcription factor regulon inference from a multicohort pulmonary fibrosis atlas confirmed elevated *NFATC4* activity in disease fibroblasts, revealing a core 119-gene *NFATC4*-dependent fibrotic program with *CTHRC1* as a top target. Phosphomimetic S213D/S217D mutants drove myofibroblast differentiation on soft substrates independently of *TGFB*, while phospho-dead S213A/S217A mutants blocked differentiation even on stiff matrix with *TGFB*, establishing the phospho-switch as both necessary and sufficient. Stiff matrix and *TGFB* converged on this *JNK*- and calcineurin-dependent switch to amplify the fibrotic response. This positions *NFATC4* S213/S217 as a mechanosensitive checkpoint for *CTHRC1*^+^ myofibroblast fate and a candidate therapeutic target in multiorgan fibrosis.

## Abstract 2

How tissue stiffness regulates identity and activity of tissue fibroblasts is unclear. Mass spectrometry based analysis of tissue stiffness dependent phosphoproteome changes reveals how primary lung fibroblasts sense the mechanical properties of their environment. Using integrative computational analysis we predicted a core mechanosensitive gene set in lung fibrosis and identified NFATC4 as a novel regulator of the stiffness dependent transition of fibroblasts to ECM secreting myofibroblasts.

## Introduction

Cells sense and respond to mechanical inputs from their environment by converting extracellular forces into intracellular chemical signals that shape cell identity and function (1, 2). These mechanosensitive responses are fundamental to embryonic development and adult tissue homeostasis, and their dysregulation contributes to diseases including atherosclerosis, cancer, and fibrosis (3, 4). While key mechanosensing effectors such as *YAP1*/*TAZ* and RhoA have been identified, the broader signaling landscape, particularly the phosphorylation events that encode mechanical information, remains poorly characterized. Mass spectrometry–based (MS-based) phosphoproteomics offers an unbiased approach to map these events at proteome scale (5).

Fibrosis is a major unmet medical need, responsible for up to 45% of deaths in industrialized countries, and is driven by the aberrant activation of fibroblasts into contractile, matrix-secreting myofibroblasts (6, 7). In the lung, physiological ECM stiffness (0.2–2 kPa) maintains fibroblast quiescence, whereas fibrotic stiffness (2–35 kPa) induces a profibrogenic phenotype with elevated proliferation and matrix synthesis (8). This mechanical feedback, termed mechanoreciprocity, operates through connections between cell-matrix adhesions and the actomyosin cytoskeleton, amplifying contractile forces in response to increasing ECM stiffness (9). Myofibroblast transition can be stabilized by tensile forces even in the absence of *TGFB* (10), suggesting that mechanical signals are not merely permissive but can be sufficient drivers of fibroblast activation. Yet, the specific signaling events that transduce stiffness into transcriptional reprogramming remain unknown.

Protein phosphorylation on serine, threonine, and tyrosine residues is among the most prevalent and functionally consequential posttranslational modifications, regulating protein conformation, interactions, and subcellular localization across virtually all signaling contexts (11, 12). In the context of cell-matrix adhesions, thousands of phosphorylation sites are regulated in an integrin-dependent manner (13), and cytoskeletal tension propagates to the nucleus to influence differentiation and gene expression (14, 15). Identifying which of these phosphorylation events are mechanistically decisive, rather than merely correlative, requires functional validation with site-specific perturbations.

scRNA-seq studies have revealed that activated myofibroblasts in fibrotic tissues are marked by expression of *CTHRC1* (collagen triple helix repeat containing 1), a secreted glycoprotein that emerges specifically in pathogenic fibroblast populations across lung, heart, and liver fibrosis (16–20). In pulmonary fibrosis, *CTHRC1*^+^ fibroblasts represent a transcriptionally distinct, high collagen–producing subpopulation that expands with disease severity and is absent in healthy lungs (16). Lineage tracing and longitudinal scRNA-seq studies suggest that the fibroblast-to-*CTHRC1*^+^ myofibroblast transition is a key activation step in fibrotic remodeling (17, 21, 22). The upstream signals that drive fibroblasts into this *CTHRC1*^+^ state, and whether mechanical cues are sufficient to do so, have not been established.

*NFATC4* is a calcium- and calcineurin-regulated transcription factor (TF) whose nuclear localization is controlled by concerted dephosphorylation of conserved serine residues in its regulatory domain (23). *JNK* has been shown to phosphorylate *NFATC4* family members and modulate their nuclear localization and transcriptional activity in a context- and domain-dependent manner (24, 25), positioning *JNK*/*NFAT* signaling as a potential integrator of mechanical and cytokine inputs in fibroblasts. *NFAT* signaling has been implicated in myofibroblast differentiation in cardiac fibrosis (26), and a recent spatial transcriptomic study identified *NFATC4* transcriptional activity as enriched in *CTHRC1*^hi^ myofibroblasts of pulmonary fibrosis patients (27). Whether specific phosphorylation sites on *NFATC4* act as a decisive switch controlling nuclear localization and entry into the *CTHRC1*^+^ myofibroblast state in the context of mechanical signaling has not been investigated.

Here, we chart the global phosphoproteome dynamics of primary human lung fibroblasts (pHLFs) across a physiologically relevant stiffness range using fibronectin-coated polydimethylsiloxane (PDMS) substrates, identifying the signaling threshold at which cells commit to a *CTHRC1*^+^/*ACTA2*^+^ myofibroblast state. We integrate this phosphoproteome map with TF regulon analysis of a multicohort pulmonary fibrosis scRNA-seq atlas and with *NFATC4* protein quantification in micro-CT staged patient tissues to nominate mechanosensitive transcriptional regulators active in human disease. Finally, using site-specific phosphomimetic and phosphoablative *NFATC4* mutants together with chemical inhibitors, we dissect the necessity and sufficiency of the S213/S217 phospho-switch in controlling fibroblast cell fate and define its relationship to mechanically and *TGFB*-driven fibrogenesis. Together, our findings establish a phosphorylation-based mechanism linking ECM stiffness to myofibroblast identity and suggest the *NFATC4* S213/S217 phospho-switch as a candidate therapeutic target in multiorgan fibrosis.

## Results

### Matrix stiffness activates human lung fibroblasts.

**Figure 1:**
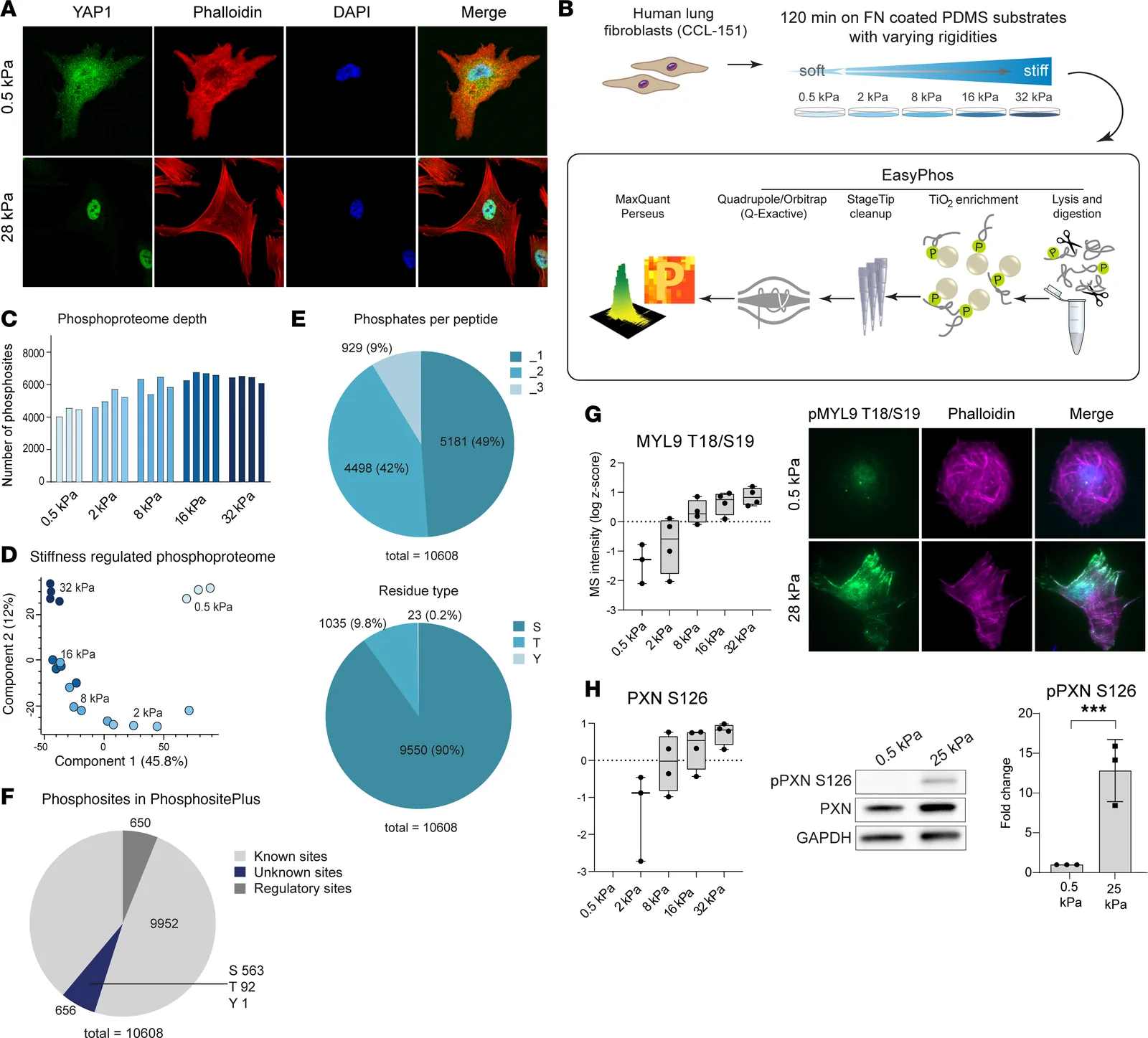
Human lung fibroblasts activate mechanosensitive signaling pathways on stiff ECM substrates. (**A**) Immunofluorescence of *YAP1* (green) in CCL-151 cells seeded for 2 h on fibronectin*-*coated substrates of indicated stiffness. Original magnification, ×40. (**B**) Experimental workflow for phosphoproteomic analysis of CCL-151 cells on varying substrate stiffnesses. FN, fibronectin. (**C**) Bar graph showing the number of quantified phosphosites per condition. (**D**) PCA of the stiffness-regulated phosphoproteome. (**E**) Pie charts showing the proportion of singly, doubly, and triply phosphorylated peptides (top) and phosphorylated S, T, and Y residues (bottom). (**F**) Pie chart showing the proportion of known, previously unreported, and regulatory phosphosites in PhosphoSitePlus. (**G**) Box-and-whisker plots of *MYL9* T18/S19 phosphopeptide intensities across stiffness conditions. Immunofluorescence with phosphospecific *MYL9* T18/S19 antibody (green) confirms stiffness-dependent induction; phalloidin (magenta) marks actin; DAPI (blue) marks nuclei. Original magnification, ×40. (**H**) Box-and-whisker plots of *PXN* S126 phosphopeptide intensities across stiffness conditions. Immunoblot with phosphospecific *PXN* S126 and total *PXN* antibodies confirms stiffness-dependent induction; *GAPDH* loading control (*n* = 3). Band intensities were quantified by densitometry, normalized to GAPDH, and compared between conditions by unpaired 2-tailed Student’s *t* test. ***P = 0.0007. Box-and-whisker plots show median, IQR, and whiskers to 1.5× IQR.

Fibroblasts from a normal healthy male human lung (CCL-151 cells) can be activated by matrix stiffness into a profibrogenic state, characterized by increased proliferation and matrix synthesis rates (8). We seeded CCL-151 cells for 120 min on fibronectin-coated PDMS substrates with a stiffness of 0.5 and 28 kPa and assessed the nuclear versus cytoplasmic localization of the mechanosensitive transcriptional regulator *YAP1* (Figure 1A). *YAP1* is known to translocate to the nucleus in a mechanical force– and cell shape–dependent manner (28, 29). Indeed, in CCL-151 cells, we found nuclear shuttling on stiff 28 kPa PDMS substrates, whereas on soft 0.5 kPa substrates, *YAP1* was localized throughout the whole cell (Figure 1A).

We next generated deep phosphoproteomes of CCL-151 lung fibroblasts during stiffness sensing. CCL-151 cells were seeded for 120 min on fibronectin-coated PDMS gels with varying rigidities from soft to stiff (0.5, 2, 8, 16, and 32 kPa) (Figure 1B). We identified 10,608 class I phosphosites with a localization probability of the phosphosite > 0.75. In each replicate included in the analysis, at least 4,000 phosphosites were measured (Supplemental Table 1; supplemental material available online with this article; https://doi.org/10.1172/JCI195121DS1). With increasing stiffness, more phosphorylation sites were recorded, in the range from 5,400 and 6,700 sites (Figure 1C). Principal component analysis (PCA) of the phosphopeptide quantification showed a good separation of the distinct substrate rigidities and high reproducibility between the single replicates (Figure 1D). The 10,608 class I phosphosites correspond to mainly serine residues (S) with 9,550 sites, followed by 1,035 phosphothreonines (T) and only 23 phosphorylated tyrosine residues (Y) (Figure 1E). Almost half of the 5,181 identified phosphosites are localized on phosphopeptides, which have only 1 phosphate group as a modification. However, 4,498 phosphosites are on doubly phosphorylated peptides, and 929 sites are located on phosphopeptides with at least 3 phosphorylations (Figure 1E). We found 656 phosphosites that were previously unknown, with no entry in the PhosphoSitePlus database (www.phosphosite.org). Furthermore, 650 phosphorylation sites identified were already well characterized and known to be functionally relevant (Figure 1F). After filtering for sites at least measured 3 times in 1 of the stiffness conditions, 8,349 phosphorylation sites remained and were used for further analysis.

To validate the experimental setup, we inspected well-known mechanosensitive phosphosites. Phosphorylation of T19 and S20 on myosin regulatory light chain (*MYL9*) is needed for cells to increase their contractility (13). Due to mechanoreciprocity, contractility of cells should increase on stiff substrates. In agreement with this prediction, we observed a gradual increase in *MYL9* T19/S20 with substrate stiffness in the phosphoproteome data, which we also validated using immunostainings (Figure 1G). Phosphorylation of S126 on the focal adhesion protein paxillin is known to regulate cytoskeletal remodeling and turnover of paxillin in focal adhesions (30). Consistent with the observed change in contractility and cytoskeletal architecture of CCL-151 on stiff substrates, we also observed highly significant changes in *PXN* S126 in both phosphoproteomes and Western blotting (Figure 1H).

In summary, the profibrogenic phenotype of CCL-151 lung fibroblasts on stiff substrates (8) is accompanied by a massive remodeling of the phosphoproteome, including induction of the YAP1 signaling pathway, increased cell contractility, and cytoskeletal rearrangements.

### The signaling landscape of fibroblast mechanosensing.

**Figure 2:**
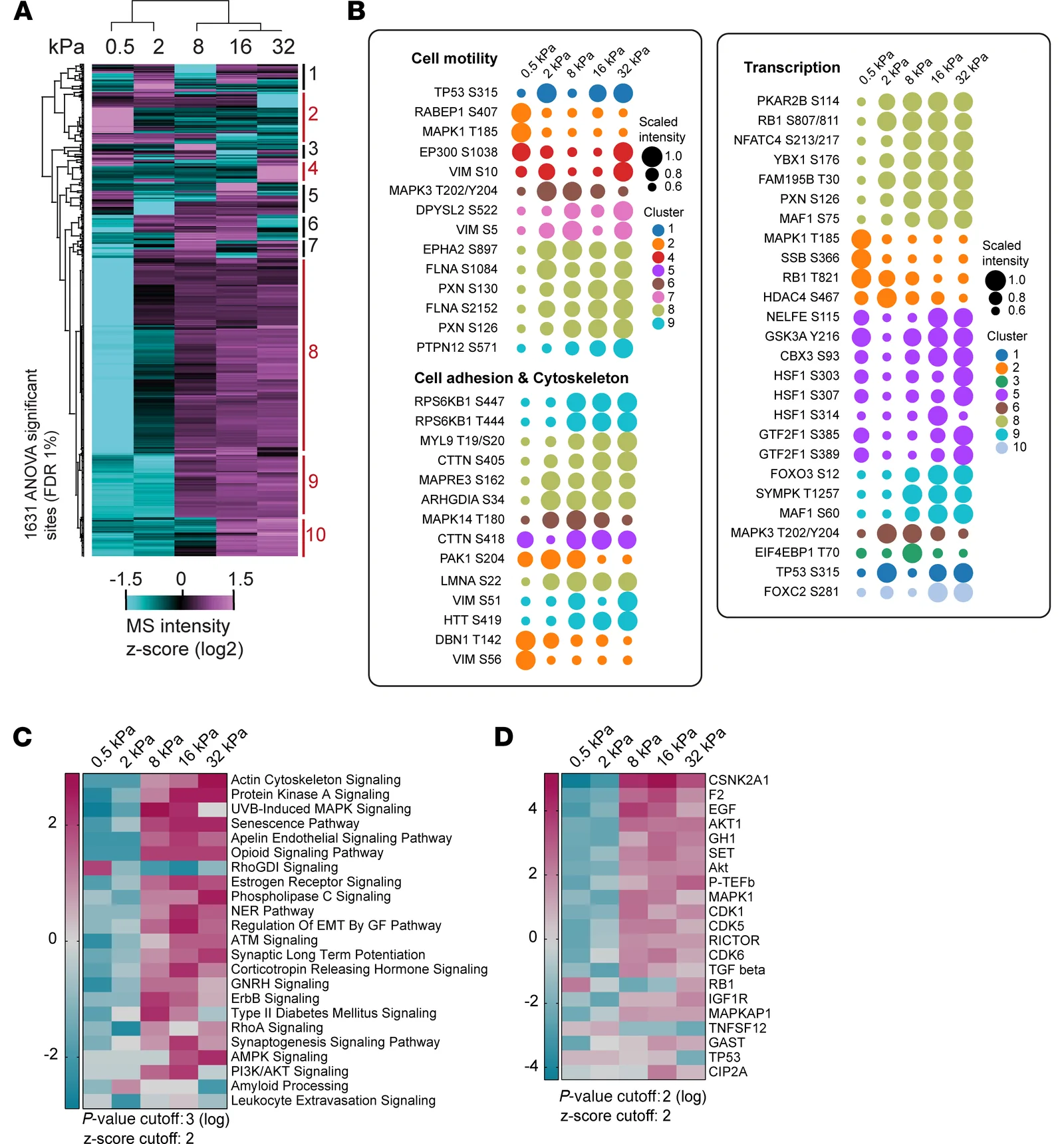
Phosphoproteomics reveals mechanosensitive signaling pathways. (**A**) Heatmap of hierarchically clustered (Pearson) *z* scored MS intensities showing stiffness-dependent phosphorylation differences; 5 of 10 clusters exhibited clear stiffness-associated patterns (red numbers). (**B**) Dot plots showing scaled phosphorylation dynamics across soft to stiff substrates for selected phosphosites with known regulatory functions (PhosphoSitePlus), color coded by cluster. (**C** and **D**) IPA of phosphoproteomic data; enrichment significance shown as log *P* value shading for canonical pathways (**C**) and predicted upstream regulators (**D**).

An ANOVA test revealed 1,631 significantly regulated phosphosites across the different substrate rigidities (1% FDR) (Figure 2A and Supplemental Table 2). These regulated sites account for around 20% of the phosphorylation sites in the entire dataset and belong to 721 individual proteins. Mainly phosphosites with phosphorylation on serine residues were found to be regulated, namely, 1,463 sites, compared with 164 threonines and 4 tyrosines. More than half of the regulated sites increased phosphorylation on stiff substrates. We identified 10 clusters containing between 52 and 648 phosphorylation sites, which showed different patterns of regulation (Figure 2A).

Using the PhosphoSitePlus database, we identified 112 significantly regulated phosphosites with known regulatory function. This allowed us to assign stiffness-regulated phosphosites to previously characterized molecular processes in cell adhesion, cytoskeletal reorganization, cell motility, and transcription regulation (Figure 2B). Several kinases involved in cell adhesion and cell motility were differentially phosphorylated on known functionally relevant sites. For instance, *PAK1* S204, which regulates conformation and enzymatic activity of *PAK1* and plays an important role in the formation of nascent adhesions and cell protrusions (31), was downregulated on stiff substrates. Also, the phosphorylation of *MAPK*1 T185 was downregulated on stiff substrates, indicating higher *MAPK*1 activity in physiologically soft environments (32). The *PTPN12* S571 site, which regulates interaction of this phosphatase with the focal adhesion kinase (*FAK*) and thereby enables dephosphorylation of *FAK* Y397 (33), was increased with stiffness. Many cytoskeleton associated proteins were differentially phosphorylated at important regulatory sites. For instance, *DBN1* T142 blocks the cryptic F-actin–bundling activity of the actin filament binding protein Drebrin (34), which we found gradually decreased with increasing substrate stiffness (Figure 2B). This observation could partially explain the increased F-actin bundling and resulting stress fiber formation on stiff substrates (Figure 1G).

Alterations in ECM substrate stiffness could induce long-term gene expression changes via differential activity of transcription and chromatin remodeling factors. Sites with known regulatory functions on the transcriptional regulators *RB1*, *PKAR2B*, *YBX1*, *NFATC4*, *FAM95B*, *MAF1*, *SYMPK*, and *FOXO3* were gradually increased with substrate stiffness (Figure 2B). To identify the most functionally relevant mechanosensitive transcriptional regulators from this dataset, we applied an additional specificity criterion: we focused exclusively on TFs carrying stiffness-regulated phosphosites at residues with documented roles in controlling transcriptional activity, yielding a high-confidence set of 6 candidates: *EP300*, *TP53*, *NFATC4*, *FOXO3*, *YBX1*, and *RB1*.

Most significantly regulated phosphosites do not have known regulatory functions yet. To reveal possible connections between sites, we mapped selected sites onto canonical signaling pathways (Supplemental Figure 2). Sections of the HIPPO, actin cytoskeleton, integrin, phospholipase C, *RHOA*, *PI3K/AKT*, p70S6K, *ERK*, p38 *MAPK*, and *mTOR* signaling pathways were manually merged and enriched with the phosphosite data from the rigidity sensing experiment. Selected sites were color coded according to the 5 most striking dynamic profiles, which consisted of sites that were generally higher on soft substrates, and then clusters that started to emerge at different levels of rigidity (Supplemental Figure 2).

Finally, we searched for significantly altered pathways based on the phosphosite quantification to score potential upstream regulators in each of the rigidity conditions using the Ingenuity Pathway Analysis platform (IPA; Qiagen). The analyses predicted altered pathways (Figure 2C) and key upstream kinases (Figure 2D) and indicated that a stiffness of 8 kPa represents an important signaling threshold in lung fibroblasts at which the activity of kinases and associated regulation of signaling pathways was drastically altered. To analyze the data, we visualized predicted upstream kinases for selected mechanosensitive phosphosites (Supplemental Figure 1). In an alternative approach, we used Metascape (35) for pathway enrichment analysis (Supplemental Figure 3A).

Casein kinase II (CK-II, CK2A1, CSNK2A1) was identified as the top upstream regulator by the IPA platform (Figure 2D). This result was inferred based on the phosphorylation status of known CK2A1 targets (Supplemental Figure 3B). A Fischer’s exact test was performed with the sites predicted by IPA to be CK2A1 targets to identify statistically enriched protein annotations (Supplemental Figure 3C). Terms such as “regulation of translation” and “mRNA processing” as well as the CK2A1 substrate motif were found to be overrepresented. To validate the in silico finding of enhanced CK-II activity in vitro, cell lysates of CCL-151 cells, which were seeded for 120 min on 0.5 and 25 kPa substrates, were probed with an antibody specific for the phosphorylated pS/pTDXE motif, a CK2A1 consensus sequence (36). This assay independently validated the stiffness-dependent increase in CK2A1 activity, which was sensitive to the specific CK-II inhibitor TBB (Supplemental Figure 3D).

To validate our findings, we repeated the phosphoproteome analysis in freshly isolated pHLFs, identifying 7,191 class I phosphosites with consistent stiffness-dependent regulation of *MYL9* T18/S19 and *PXN* S126 (Supplemental Figure 4). Integration of the CCL-151 and pHLF datasets identified 290 shared mechanosensitive sites (Supplemental Figure 5), confirming a core mechanosensing phosphoproteome conserved across cell systems (see more details in Supplemental Results). We also performed a time-resolved phosphoproteome analysis on stiff substrates to derive the temporal sequence of events during this mechanosensing response (see Supplemental Table 2, Supplemental Figures 4–9, and Supplemental Results).

### Phosphoproteome TF activity inference predicts in vivo regulon scores.

Lung fibrosis in patients leads to a stiffened microenvironment; therefore, many of the altered TF activities in our phosphoproteomic screen should be observed also in patients. We next tested if our in vitro observations hold relevance also for lung fibrosis in vivo, hypothesizing that predictions about TF activity based on phosphoproteomic data should agree with TF activity inference from single-cell transcriptomic data. Posttranslational modifications such as phosphorylation control TF activity; hence, it is often more powerful to infer TF activity from a set of known target genes rather than analyzing the TF expression level directly. Importantly, the 6 TFs selected for in vivo validation — *EP300*, *TP53*, *NFATC4*, *FOXO3*, *YBX1*, and *RB1* — were not chosen based on prior hypothesis but emerged exclusively from the phosphoproteomic data as the transcriptional regulators carrying stiffness-dependent phosphorylation at functionally characterized activity-controlling residues. The following analysis therefore serves as orthogonal validation of mechanosensitive TF activity predictions made in vitro, using independent in vivo transcriptomic data from patients with idiopathic pulmonary fibrosis (IPF).

**Figure 3:**
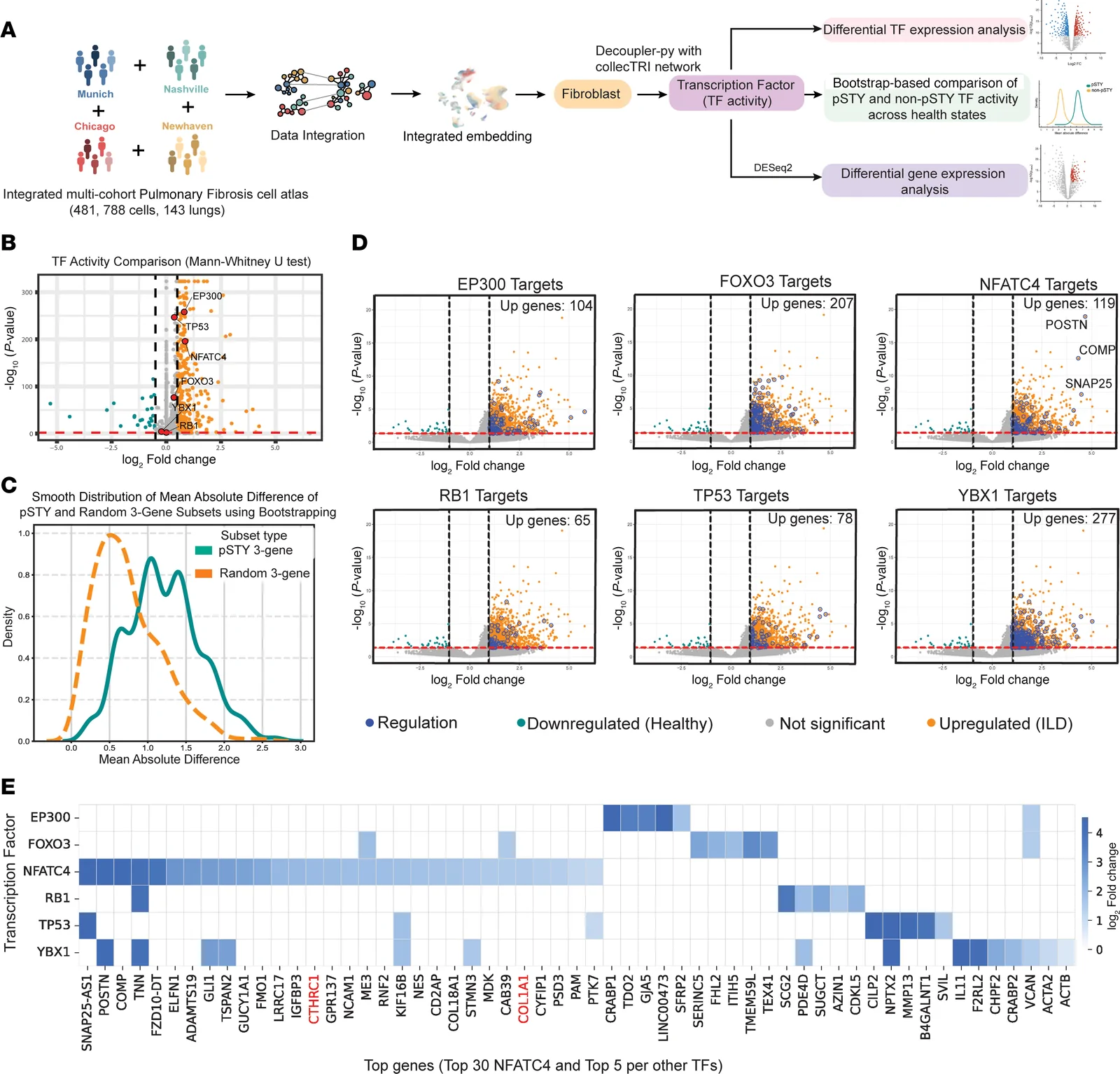
A core mechanosensitive gene set downstream of differentially phosphorylated transcriptional regulators in vivo. (**A**) Analysis workflow. (**B**) Volcano plot of differential TF activity (healthy vs. pulmonary fibrosis fibroblasts), highlighting TFs with stiffness-dependent regulatory phosphosites (*EP300*, *FOXO3*, *NFATC4*, *RB1*, *TP53*, *YBX1*). (**C**) Bootstrap comparison of mean absolute TF activity differences for the stiffness-dependent TF subset versus random TF combinations. (**D**) Volcano plots of differential target gene expression (pulmonary fibrosis vs. controls) per TF regulon, with significantly upregulated genes highlighted (|log_2_FC| > 1 and FDR-adjusted *P* < 0.05; Mann-Whitney *U* test). (**E**) Heatmap of the 30 top upregulated *NFATC4* target genes and the 5 top targets for each remaining TF in IPF versus healthy fibroblasts; color intensity reflects upregulation magnitude.

Using this concept, we can compare the direction of inferred TF activity from gene expression data in patients with the predictions made based on phosphorylation data. To test our hypothesis, we first performed regulon analysis on a large scRNA-seq dataset integrating several patient cohorts (Figure 3A) (37). We compared the transcriptomic signature of fibroblasts from patients with pulmonary fibrosis to those from healthy patients acting as healthy controls and derived a set of differentially expressed genes that were used to predict differential TF activity based on prior knowledge databases and the pySCENIC tool, a data-driven gene regulatory network framework that constructs TF regulons de novo from single-cell transcriptomic data based on coexpression and DNA motif enrichment. Using activity inference with pySCENIC-derived regulons, we confirmed that *EP300*, *TP53*, *NFATC4*, and *FOXO3* showed significantly increased TF activity scores from in vivo IPF fibroblast expression data (Figure 3B). We plotted the distribution of 3 randomly selected TF regulon activities from the in vivo data and found that any combination of 3 factors out of our selection of 6 target factors was significantly enriched in the lung fibrosis group compared with random picks (Figure 3C). This strongly suggested to us that the predictions made through our phosphoproteomic analysis are at least partially confirmed in scRNA-seq data from lung fibrosis patients.

We next examined which known target genes of these TFs were upregulated in fibroblasts from IPF lungs, reasoning that the intersection of prior knowledge gene sets with in vivo differential expression would reveal a core mechanosensitive transcriptional program (Figure 3, D and E). Among the 6 TFs, *NFATC4* emerged as the most compelling mechanosensitive regulator, driving the expression of the 3 most highly upregulated genes and 119 significantly induced targets (log_2_FC > 1, adjusted *P* < 0.05). These targets included established mediators of fibroblast activation (*IGFBP3*, *GLI1*), extracellular matrix remodeling and collagen cross-linking (*POSTN*, *COMP*, *PLOD2*, *COL18A1*, *COL6A2*), myofibroblast-associated matrix turnover and *TGFB* activation (*LTBP3*, *MMP2*, *MRC2*, *AEBP1*), and *CTHRC1* and *COL1A1*, directly highlighting *NFATC4* as a central upstream regulator of fibrotic remodeling programs (Figure 3, D and E). Given the stiffness-dependent phosphorylation of *NFATC4* at key regulatory sites observed in our in vitro experiments, these findings suggest that mechanical activation of *NFATC4* drives a broad and clinically relevant transcriptional program in fibrotic lung fibroblasts in vivo.

### NFATC4 expression progressively increases with IPF disease severity in human lung tissue.

Tissue stiffness is increased during lung fibrosis, which coincides with a transition of fibroblasts into *CTHRC1*^+^/*ACTA2*^+^ contractile myofibroblasts that secrete large amounts of collagens and invade the tissue (16, 38). To determine the clinical relevance of *NFATC4*-mediated mechanosensing in human fibrotic disease, we examined *NFATC4* expression costained with the canonical myofibroblast markers *CTHRC1* and *ACTA2* in lung tissue sections from healthy donors and patients with IPF. The IPF samples were stratified by disease severity into mild (IPF1), moderate (IPF2), and severe (IPF3) degree of tissue remodeling using micro-CT staging, as previously reported (39).

**Figure 4:**
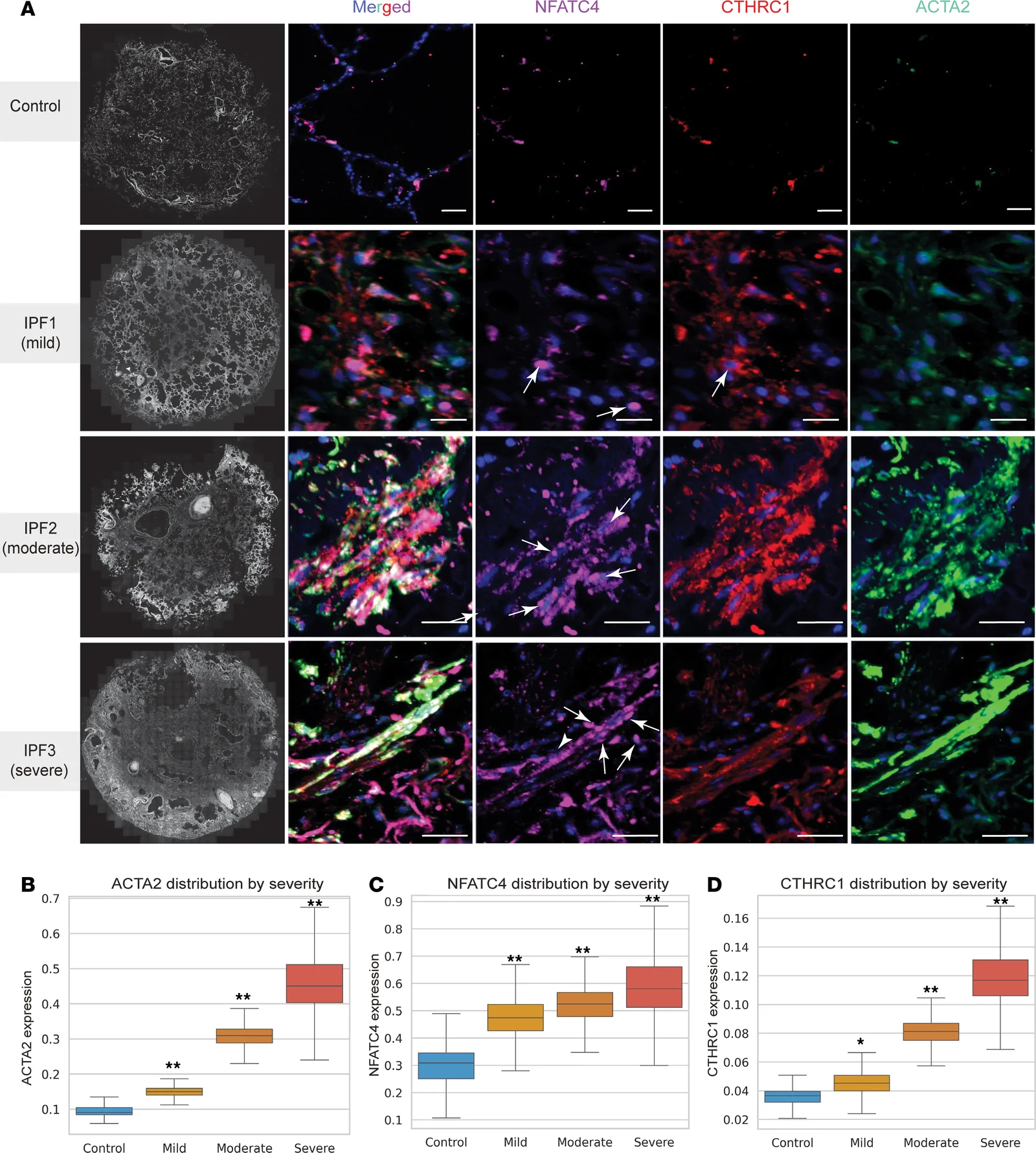
*NFATC4*, *CTHRC1*, and *ASMA* expression increases with IPF disease severity in human lung tissue. (**A**) Representative immunofluorescence images of lung tissue sections from control donors and IPF patients with mild (IPF1), moderate (IPF2), and severe (IPF3) disease. Left column: low-magnification overview (section diameter: 10 mm). Right columns: high-magnification images showing *NFATC4* (magenta), *CTHRC1* (red), *ASMA* (green), and DAPI (blue). The arrows indicate colocalization of *NFATC4* in the nucleus. Scale bars: 50 μm. (**B**–**D**) Quantification of normalized expression levels for *ASMA* (**B**), *NFATC4* (**C**), and *CTHRC1* (**D**) across disease severity stages. Box-and-whisker plots show median, IQR, and whiskers to 1.5× IQR. Data represent mean ± SEM (*n* = 3). Statistics: *P < 0.05, **P < 0.001 versus control; Kruskal-Wallis test with pairwise Mann-Whitney *U* post hoc tests and Bonferroni correction for multiple comparisons.

Immunofluorescence staining revealed minimal expression of all 3 proteins in the parenchyma of healthy control lung tissue, with *ACTA2* restricted to vascular smooth muscle cells (Figure 4A). In contrast, mild IPF samples (IPF1) showed increased expression of *NFATC4*, mostly showing nuclear localization in cells with elevated *CTHRC1* and *ACTA2* expression. This coordinated upregulation of *NFATC4* and myofibroblast markers became progressively more pronounced in moderate (IPF2) and severe (IPF3) samples, where extensive colocalization of all 3 markers was observed in dense fibrotic foci.

Quantitative analysis confirmed significant progressive increases in *ACTA2* (Figure 4B), *NFATC4* (Figure 4C), and *CTHRC1* (Figure 4D) expression correlating with disease severity (*P* < 0.05). These data indicate a potential role of *NFATC4* in myofibroblasts in IPF tissues and suggest that the mechanotransduction pathway we characterized in cell culture models might also be operational in human IPF.

### NFATC4 regulates collagen secretion, cell invasion, and contractility.

**Figure 5:**
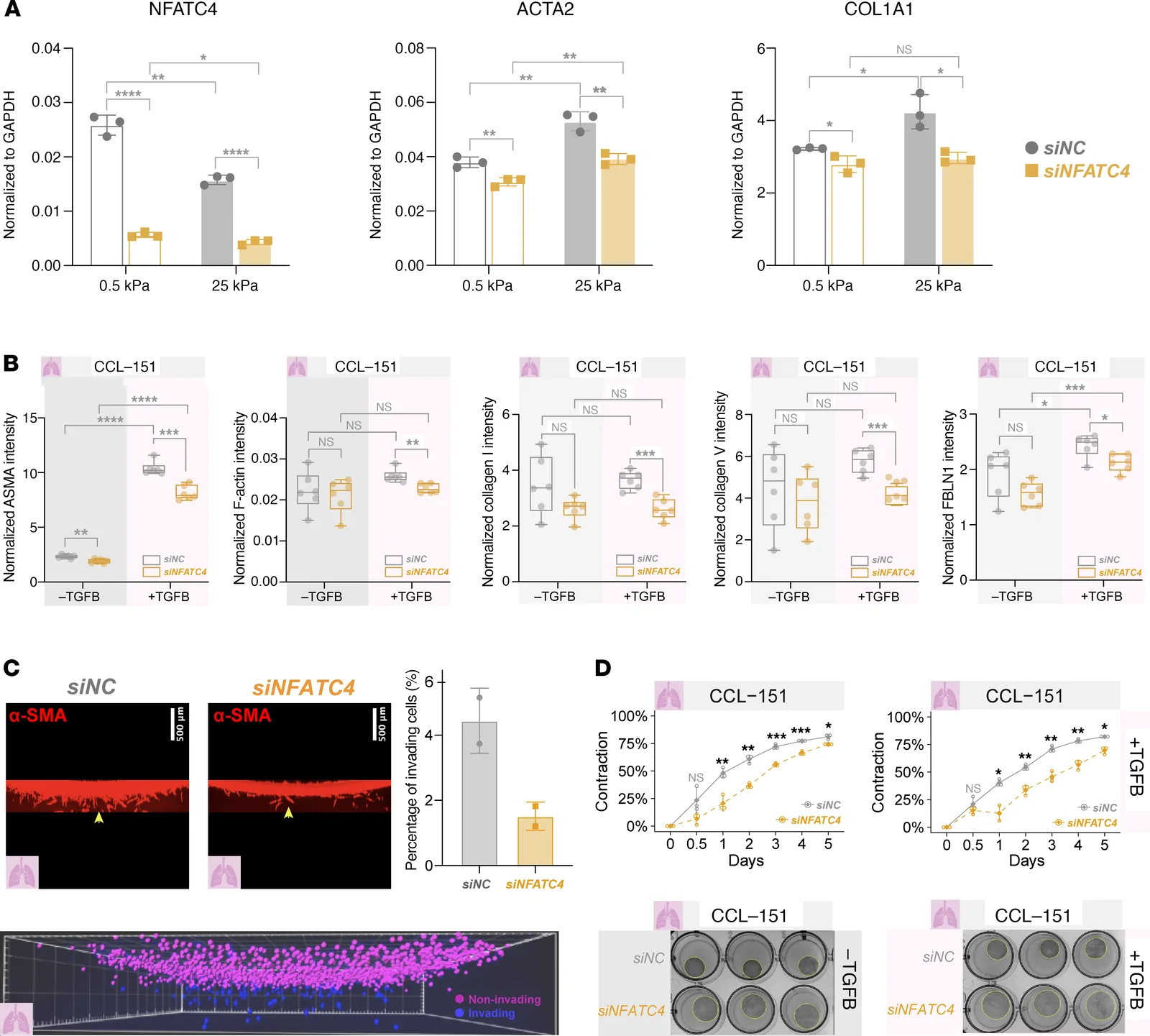
*NFATC4* knockdown attenuates fibroblast-to-myofibroblast transition, collagen deposition, and invasion. (**A**) qPCR of *NFATC4*, *ASMA*, and *COL1A1* mRNA in CCL-151 transfected with siNC or si*NFATC4* on stiff and soft substrates (*n* = 3). (**B**) Fluorescence intensity of intracellular *ASMA*, F-actin, and extracellular collagen type I, collagen type V, and Fibulin-1, normalized to DAPI (*n* = 6). (**C**) Collagen invasion assay. Confocal immunofluorescence images after 3D projection; *ASMA* staining (red); yellow arrow indicates an invasive cell. Scale bar: 500 μm. 3D visualization in Imaris (bottom panel). Quantification shown as relative mean ± SD from 2 replicates. (**D**) Collagen gel contraction in CCL-151 ± *TGFB* (1 ng/mL) over time (*n* = 3). Representative images at day 3 shown below; yellow circles outline gel pads. Statistics: *P < 0.05, **P < 0.01, ***P < 0.001, ****P < 0.0001; unpaired 2-tailed Student’s *t* test with Bonferroni correction for multiple comparisons where applicable. Box-and-whisker plots show median, IQR, and whiskers to 1.5× IQR.

To test the functional relevance of *NFATC4* in fibroblast (patho-)physiology, we used siRNA to knock down *NFATC4* expression in CCL-151 (Figure 5) and pHLFs from a patient with IPF (Supplemental Figure 10). *NFATC4* expression was consistently downregulated > 70% for at least 2–3 days after siRNA transfection. Interestingly, we observed slightly reduced levels of *NFATC4* mRNA in cells plated on stiff substrates compared with soft ones (Figure 5A). Stiff substrates induced the expression of the myofibroblast marker *ACTA2*, as well as collagen type I (*COL1A1*), both of which were significantly reduced in *NFATC4* knockdown cells (Figure 5A), indicating that *NFATC4* indeed is involved in myofibroblast biology.

In addition to qPCR-based testing of gene expression, we analyzed *ACTA2* and F-actin levels as well as the expression, secretion, and assembly of *COL1A1*, collagen type V (*COL5A1*), and Fibulin-1 (*FBLN1*), all of which are key hallmarks of myofibroblast identity, using antibody-based immunofluorescence analysis (Figure 5B). Cells were plated on stiff fibronectin-coated tissue culture plates and treated with *TGFB* to boost fibroblast-to-myofibroblast conversion. Indeed, we observed a significant upregulation of *ACTA2* upon treatment with *TGFB* (Figure 5B). The *NFATC4* knockdown significantly reduced the amount of F-actin as well as protein amounts of *ACTA2*, *COL1A1*, *COL5A1*, and *FBLN1* under these conditions in both CCL-151 (Figure 5B) and IPF-derived pHLFs (Supplemental Figures 11 and 12).

*CTHRC1*^+^/*ACTA2*^+^ myofibroblasts have been shown to be highly invasive in vivo and in vitro (16, 38). We seeded CCL-151 fibroblasts on top of collagen gels and assessed the proportion of invading cells using confocal imaging in *Z*-stacks. Over the course of 96 hours, significantly fewer *NFATC4*-knockdown cells invaded the 3D collagen gel (Figure 5C).

Next, we asked if the ability of fibroblasts to contract a collagen type I spheroid was affected by *NFATC4* expression levels. We seeded 5 × 10^4^ CCL-151 fibroblasts (Figure 5D) or MLT-018, an IPF patient–derived pHLF (Supplemental Figure 10), into collagen gels and analyzed the contraction of the gels over time utilizing collagen contraction assay. *NFATC4*-knockdown cells showed significantly reduced levels of gel contraction in both CCL-151 and IPF-derived pHLFs.

To analyze the effect of *NFATC4* silencing on the cell contractility feedback on stiff substrates in single cells, we plated siRNA transfected cells on fibronectin-coated adhesive micropatterns with an L shape. In this system, the cells adhere to the L shape and form a contractile actomyosin bundle spanning the nonadhesive edge of the L pattern. Cytoskeleton-mediated contraction has been shown to be required for the fibroblast-to-myofibroblast transition (40) and can also affect nuclear morphology (41). Interestingly, it has been shown that fibroblasts activated by increased stiffness have less round nuclei (15). We therefore analyzed the nuclear shapes and distributions in single fibroblasts.

**Figure 6:**
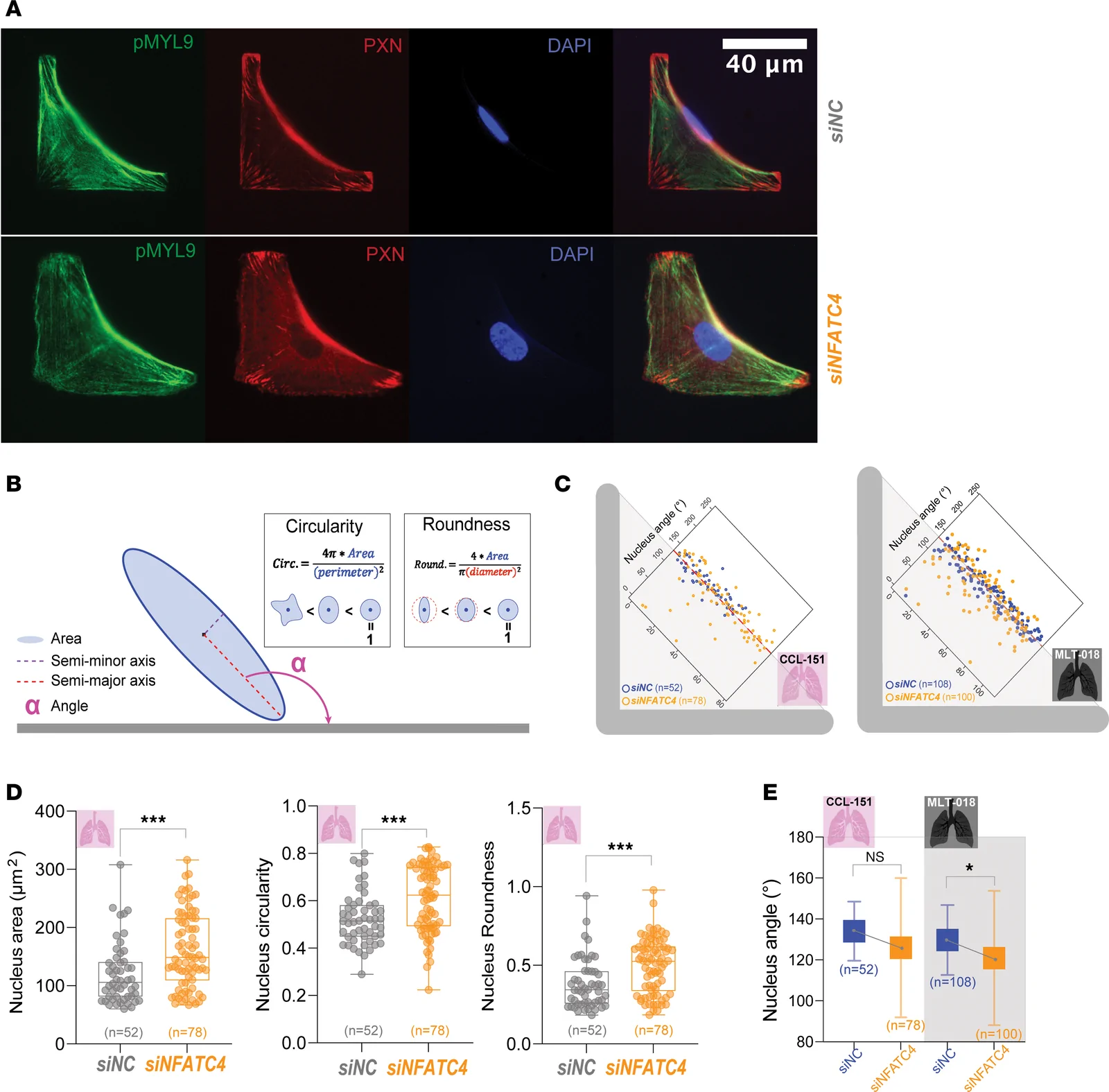
*NFATC4* knockdown alters nuclear morphology and positioning on fibronectin micropatterns. (**A**) CCL-151 fibroblasts transfected with siNC or si*NFATC4* were seeded on L-shaped fibronectin micropatterns and stained for p*MYL9* (active myosin, green), paxillin (*PXN*; focal adhesions, red), and DAPI (nuclei, blue). siNC nuclei are positioned toward the adhesion-free corner and appear compact, whereas si*NFATC4* nuclei are centrally located and more spheroid. Scale bar: 40 μm. (**B**) Schematic of the morphometric parameters used: cell area, semi-axes, orientation angle (α), circularity, and roundness. (**C**) Scatterplots of nucleus angle versus nucleus area for CCL-151 (siNC, *n* = 52; si*NFATC4*, *n* = 78) and MLT-018 (siNC, *n* = 108; si*NFATC4*, *n* = 100). (**D**) Quantification of nucleus area, circularity, and roundness in CCL-151 cells (siNC, *n* = 52; si*NFATC4*, *n* = 78). (**E**) Nucleus angle comparison between siNC and si*NFATC4* in CCL-151 and MLT-018 cells. Statistics: *P < 0.05, ***P < 0.001; paired 2-tailed Student’s *t* test on per-run means (*n* = 3). Data are presented as mean ± SD. Box-and-whisker plots show median, IQR, and whiskers to 1.5× IQR. Pink lung icon, CCL-151; black lung icon, MLT-018.

We noticed that most nuclear centroids of siNC-transfected fibroblasts were pulled toward the contractile adhesion free edge on the L-shaped micropatterns (Figure 6, A–C, and Supplemental Figure 13, A and C), which also resulted in an elongated spherical nuclear shape (Figure 6, A–C, and Supplemental Figure 13B). In contrast, the nuclei of *siNFATC4*-transfected cells were distributed more widely, with a tendency to be positioned away from the contractile edge and oriented more toward the adhesive edge (Figure 6, A–C, and Supplemental Figure 13A). The nuclei of *siNFATC4*-transfected cells displayed a significantly larger area and had a relaxed round shape (Figure 6, D and E, and Supplemental Figure 13), suggesting an overall reduction of cytoskeletal and nuclear tension in *NFATC4*-knockdown cells. F-actin stress fibers decorated with active myosin (pMLC S19/S20) were predominantly located at the contractile nonadhesive edge in siNC-transfected, while these fibers were more widely distributed in *siNFATC4*-transfected fibroblasts (Figure 6A and Supplemental Figure 13A).

### NFATC4 phosphorylation is required and sufficient to induce the fibroblast-to-myofibroblast switch.

**Figure 7:**
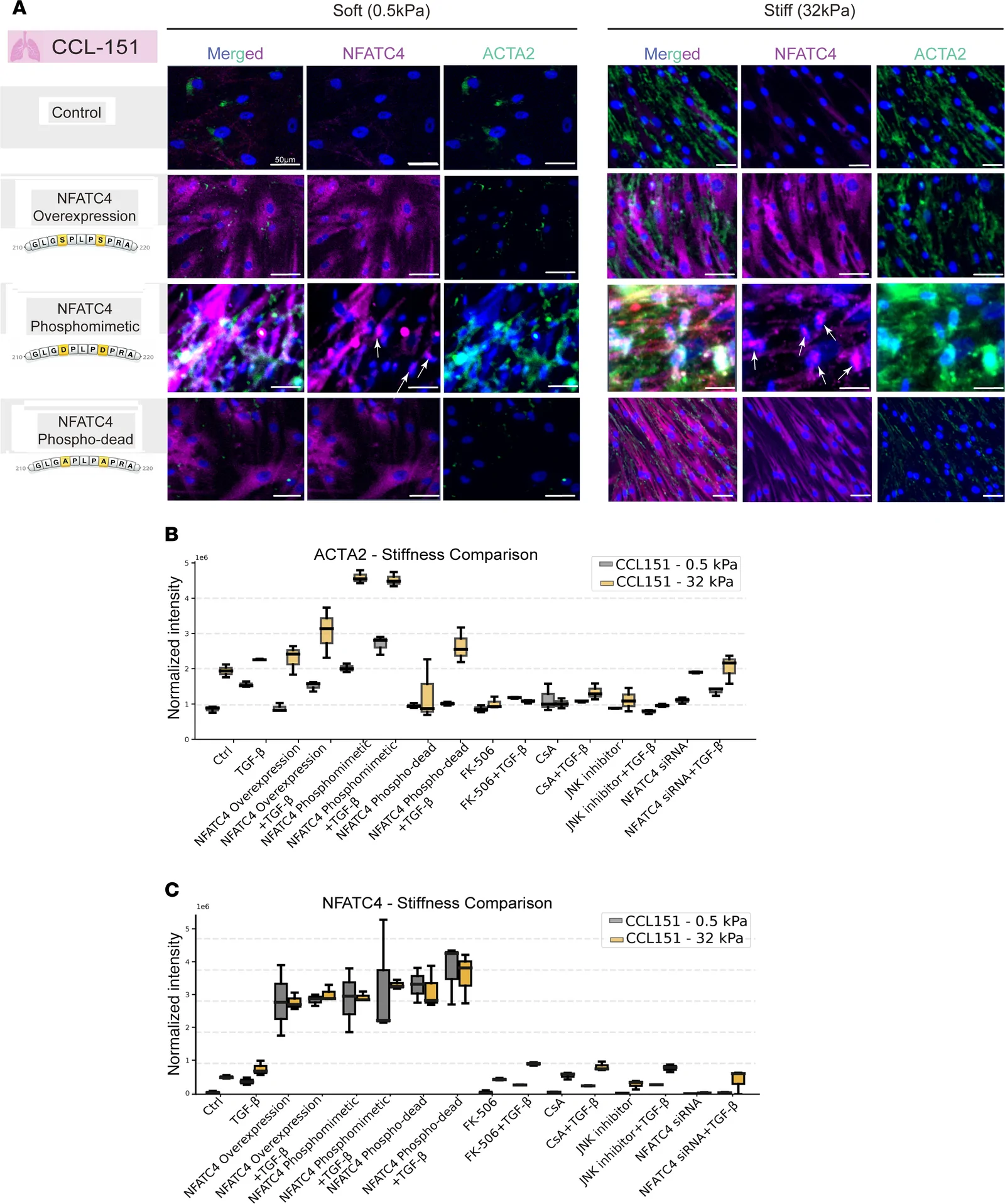
*NFATC4* phosphorylation regulates myofibroblast differentiation on soft and stiff substrates. (**A**) Representative immunofluorescence images of CCL-151 fibroblasts on soft (0.5 kPa) or stiff (32 kPa) substrates, transfected with control, WT *NFATC4*, phosphomimetic (S213D/S217D), or phospho-dead (S213A/S217A) constructs. Channels: *NFATC4* (magenta), *ASMA* (green), DAPI (blue); arrows indicate nuclear *NFATC4*. Scale bars: 50 μm. (**B** and **C**) Normalized *ASMA* (**B**) and *NFATC4* (**C**) intensity in CCL-151 cells on soft (gray) versus stiff (orange) substrates across conditions; data are presented as mean ± SD. Box-and-whisker plots show median, IQR, and whiskers to 1.5× IQR. Statistics: pairwise 2-sample *z* tests (*n* = 3 biological replicates); full results are provided in Supplemental Table 5.

**Figure 8:**
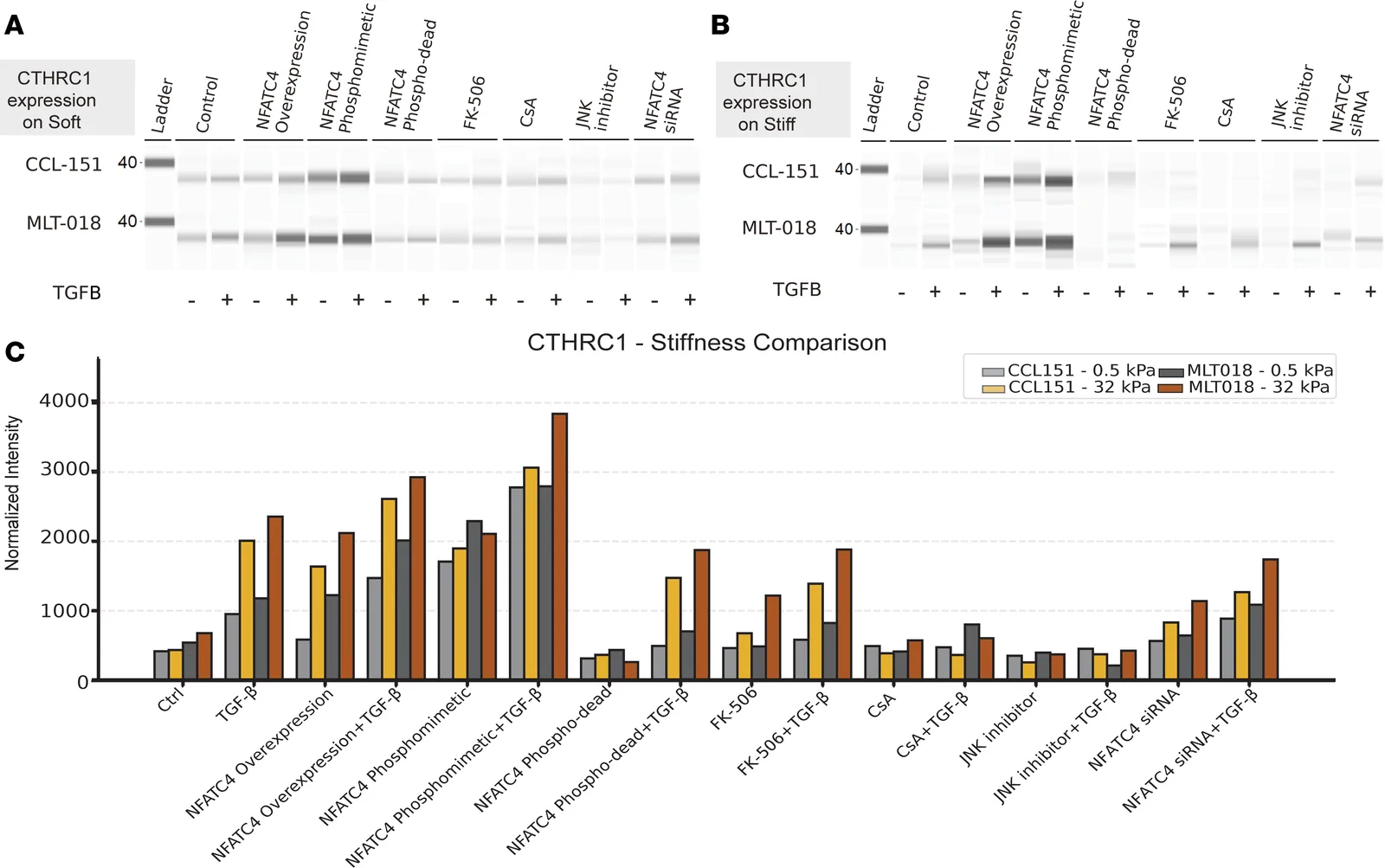
CTHRC1 protein expression in CCL-151 and MLT-018 fibroblasts across substrate stiffnesses, NFATC4 constructs, and pharmacological treatments. (**A** and **B**) Western blots of *CTHRC1* in CCL-151 and MLT-018 fibroblasts on soft (**A**) or stiff (**B**) substrates with *NFATC4* constructs and inhibitors ± *TGFB* (5 ng/mL). (**C**) *CTHRC1* band intensity quantification: CCL-151 soft (light gray), CCL-151 stiff (light orange), MLT-018 soft (dark gray), MLT-018 stiff (dark orange); normalized intensity values.

To directly test the importance of *NFATC4* phosphorylation for the fibroblast-to-myofibroblast switch observed in fibrotic disease, we created phosphomimetic (S213D/S217D), phospho-dead (S213A/S217A), and WT *NFATC4* constructs and overexpressed them in CCL-151 healthy fibroblasts and IPF-derived primary fibroblasts. Transfected cells were cultured on soft (0.5 kPa) or stiff (32 kPa) fibronectin-coated PDMS substrates with and without *TGFB* stimulation. *NFAT* family TFs are known to be regulated by calcium-dependent signaling (42), and the *NFATC4* S213/217 site has been shown to be phosphorylated by *JNK* (24). Therefore, we also assessed the effects of pharmacological inhibition of the calcium-dependent pathway (FK-506, cyclosporin A) and *JNK* (*JNK* inhibitor VIII). In addition, we again used *NFATC4* siRNA for knockdown across the same conditions in both cell lines (Figures 7 and 8 and Supplemental Figure 14). The results were evaluated using Western blot and immunofluorescence staining (Supplemental Table 3).

Under untreated culture conditions, CCL-151 cells on soft substrates showed minimal *ACTA2*, F-actin, and *CTHRC1* expression, while transfer to stiff substrates induced robust upregulation of all 3 markers (Figure 7, A–C, and Supplemental Figure 14D). IPF-derived fibroblasts displayed significantly higher baseline expression of *ACTA2* and F-actin on soft substrates compared with CCL-151 cells, consistent with their constitutively activated fibrotic phenotype, yet retained a further stiffness-dependent increment on 32 kPa substrates. *TGFB* amplified *ACTA2*, F-actin, and *CTHRC1* induction on stiff substrates in both cell lines but had minimal additional effect on soft substrates, demonstrating synergy between mechanical and chemical stimuli.

WT *NFATC4* overexpression did not significantly change *ACTA2*, F-actin, or *CTHRC1* expression compared with control cells on either soft or stiff substrates, with or without *TGFB*, in both CCL-151 and IPF-derived pHLF cells (Figure 7, B and C, and Supplemental Figure 14, C and D). We also confirmed that *CTHRC1* protein levels in WT overexpressing cells were comparable with controls across all stiffness and *TGFB* conditions in both cell lines (Figure 8, A and B). These findings demonstrate that simply increasing *NFATC4* protein levels is insufficient to alter the myofibroblast differentiation program and that the functional effects of *NFATC4* are tightly linked to posttranslational regulation of the protein rather than its expression level. WT overexpression therefore serves as an important control establishing that the dramatic effects of the phosphomimetic and phospho-dead mutants are specifically attributable to the phosphorylation status at S213/S217 rather than to nonspecific consequences of *NFATC4* overexpression.

In stark contrast to WT overexpression, the phosphomimetic mutant (S213D/S217D) drove robust *ACTA2* and *CTHRC1* expression, prominent stress fiber formation, and strong nuclear *NFATC4* accumulation even on soft 0.5 kPa substrates in the absence of *TGFB* in CCL-151 cells, a condition under which control and WT overexpressing cells showed minimal myofibroblast activation (Figure 7, A and B). This demonstrates that constitutive phosphorylation at S213/S217 is sufficient to bypass the mechanical stiffness threshold required for *NFATC4* activation. On stiff substrates, the phosphomimetic mutant induced *ACTA2* and F-actin levels substantially above those of control and WT overexpressing cells (Supplemental Figure 14, C and D), and *TGFB* further amplified this response to produce the highest myofibroblast marker expression observed across the entire experiment in both CCL-151 and MLT-018 cells. Western blot analysis confirmed that *CTHRC1* protein was strongly induced by the phosphomimetic mutant on both soft and stiff substrates in both cell lines, with *TGFB* producing further enhancement and stiff substrate conditions consistently yielding higher *CTHRC1* levels than soft substrate conditions (Figure 8, A–C). In MLT-018 cells, the phosphomimetic mutant similarly induced maximal *ACTA2* and F-actin on both substrate stiffnesses, with the combined phosphomimetic plus *TGFB* condition on stiff substrates exceeding even the elevated baseline of these fibrotic cells (Supplemental Figure 14, C and D).

The phospho-dead mutant (S213A/S217A) failed to induce *ACTA2* expression, stress fiber formation, or *CTHRC1* upregulation under any condition tested in both CCL-151 and MLT-018 cells (Figure 7, A–C, and Supplemental Figure 14, C and D). Immunofluorescence confirmed that phospho-dead *NFATC4* protein was expressed at levels comparable with WT and phosphomimetic constructs but remained predominantly cytoplasmic, demonstrating that S213/S217 phosphorylation is required for nuclear translocation and transcriptional activity (Figure 6A). On stiff substrates, where control cells show robust endogenous myofibroblast activation, phospho-dead transfection suppressed *ACTA2* and F-actin to levels at or below those of untransfected controls (Supplemental Figure 14D). Western blot analysis confirmed that *CTHRC1* protein levels in phospho-dead transfected cells were markedly reduced below control levels on both soft and stiff substrates in CCL-151 and MLT-018 cells (Figure 8, A and B). Notably, *TGFB* stimulation partially restored *CTHRC1* levels in phospho-dead transfected cells, suggesting that *TGFB* can engage *CTHRC1* expression through *NFATC4*-independent mechanisms. Nevertheless, *CTHRC1* levels in the phospho-dead condition remained substantially lower than those observed in control and *NFATC4* overexpression cells under equivalent *TGFB* and stiffness conditions, demonstrating that while *TGFB* can partially compensate for these lower levels, S213/S217 phosphorylation remains the dominant regulatory input controlling *NFATC4*-dependent *CTHRC1* induction.

FK-506 and CsA both reduced *ACTA2* and F-actin on stiff substrates in CCL-151 and MLT-018 cells under basal conditions (Supplemental Figure 14D), with comparable suppression observed in both cell lines. *TGFB* partially counteracted the inhibitory effects of FK-506 and CsA on stiff substrates in both CCL-151 and MLT-018 cells, suggesting that *TGFB* can partially engage *CTHRC1* and *ACTA2* expression through calcineurin-*NFAT*–independent mechanisms in both healthy and fibrotic fibroblasts. *JNK* inhibition markedly reduced *ACTA2*, F-actin, and *CTHRC1* levels on stiff substrates in both CCL-151 and MLT-018 cells (Figure 8C and Supplemental Figure 14, C and D), confirming that *JNK*-mediated phosphorylation of *NFATC4* at S213/S217 is the critical upstream event linking mechanical stiffness to myofibroblast differentiation. Notably, the suppressive effect of *JNK* inhibition was more complete than that of FK-506 or CsA in both cell lines, consistent with *JNK* acting upstream of *NFATC4* phosphorylation. *NFATC4* siRNA knockdown produced consistent suppression of all myofibroblast markers across soft and stiff conditions and with or without *TGFB* in both CCL-151 and MLT-018 cells (Supplemental Figure 14, B–D), in full agreement with our loss-of-function data in Figure 5, and confirming that the effects of *JNK* inhibition are specifically mediated through *NFATC4*.

Finally, we performed subcellular fractionation by Western blotting, which demonstrated that *JNK* inhibition directly reduced nuclear *NFATC4* accumulation under both basal and *TGFB*-stimulated conditions (Supplemental Figure 15), mechanistically linking stiffness-activated *JNK* to *NFATC4* nuclear translocation and establishing a direct kinase-to-TF connection within the mechanosensing cascade.

**Figure 9:**
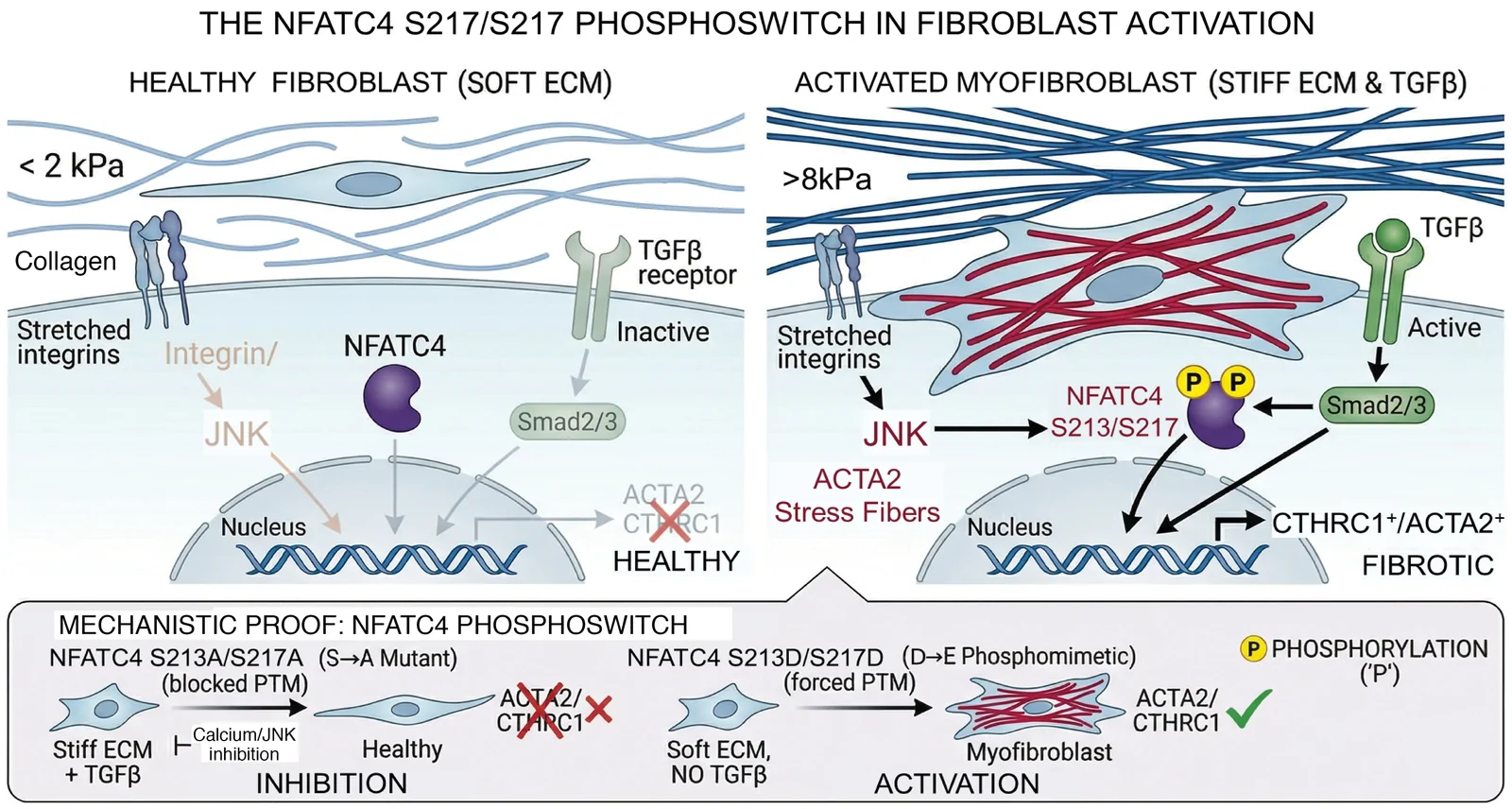
Model of the *NFATC4* S213/S217 phospho-switch controlling fibroblast-to-myofibroblast transition. On soft ECM (<2 kPa), *JNK* signaling and *TGFB*/SMAD2/3 inputs are absent, *NFATC4* remains unphosphorylated and cytoplasmic, and myofibroblast genes such as *ACTA2* and *CTHRC1* are not expressed. On stiff ECM (>8 kPa) with *TGFB*, mechanosensitive *JNK* activation phosphorylates *NFATC4* at S213/S217, driving nuclear translocation and transcriptional induction of the myofibroblast program, including *ACTA2* and *CTHRC1*, while parallel SMAD2/3 activation enhances the fibrotic gene program to firmly establish the *CTHRC1*^+^/*ACTA2*^+^ myofibroblast state. Mechanistic evidence is shown in the bottom panel: the phospho-dead mutant (S213A/S217A, blocked posttranslational modification [PTM]) fails to induce *ACTA2* or *CTHRC1* even on stiff ECM with *TGFB*; conversely, the phosphomimetic mutant (S213D/S217D, mimicked PTM) drives robust myofibroblast differentiation on soft ECM in the complete absence of *TGFB*. P, phosphorylation.

Taken together, the comprehensive gain- and loss-of-function analysis across 2 substrate stiffnesses, *TGFB* stimulation, 2 cell lines of distinct disease origin, and multiple independent inhibitory approaches establishes that phosphorylation of *NFATC4* at S213/S217 (likely by *JNK*) is both necessary and sufficient for stiffness-induced myofibroblast differentiation (Figure 9). The absence of any effect of WT overexpression on myofibroblast markers demonstrates that *NFATC4* function is governed by posttranslational regulation rather than protein abundance, and this phosphorylation-dependent mechanism is fully preserved in IPF-derived fibroblasts and cannot be bypassed by *TGFB* signaling.

## Discussion

Fibroblast-to-myofibroblast transition is a central event in fibrosis that coincides with changes to mechanical properties of the tissue. Yet, the phosphorylation-based signaling logic that converts a mechanical stimulus into a committed transcriptional cell fate decision has remained poorly defined. Here, we used unbiased phosphoproteomics of pHLFs across a physiologically relevant stiffness gradient to chart this signaling landscape at proteome scale and identified a critical mechanosensing threshold at 2–8 kPa, above which cells activate the myofibroblast program. From these data, we nominated mechanosensitive sites on transcriptional regulators, including *NFATC4* S213/S217, that had documented roles in controlling transcriptional activity, and validated these predictions through independent TF regulon analysis in a multicohort pulmonary fibrosis atlas. Gain- and loss-of-function experiments including site-specific phosphomimetic and phosphoablative mutants then established that S213/S217 phosphorylation on *NFATC4* is both necessary and sufficient to drive fibroblasts into a *CTHRC1*^+^/*ACTA2*^+^ myofibroblast state, positioning the *NFATC4* S213/S217 phospho-switch as a convergence point for mechanical and cytokine-driven fibrogenesis.

The phosphoproteomics dataset itself constitutes a resource for the field. We identified 1,631 sites regulated across 5 stiffness conditions, spanning virtually all major mechanosensing organelles and signaling nodes, such as integrin-adhesion complexes, the actomyosin cytoskeleton, the nucleus, mitochondria, and the ER. The time-resolved spreading dataset adds a kinetic dimension, revealing that early cell attachment (10–30 min) is dominated by RNA processing and cytoskeletal remodeling events, while the contractile phase (60–120 min) engages Hippo-*YAP1*/TAZ, RhoA, and mesenchymal differentiation programs. A subset of proteins, including Vimentin, *EP300*, *MAPK12*, *CTTN*, and *TP53*, displayed nonmonotonic phosphorylation profiles across the stiffness gradient rather than the gradual monotonic changes seen for most regulated sites. These irregular patterns are unlikely to reflect technical noise, given the high replicate reproducibility demonstrated by PCA. Instead, they might reflect the threshold-gated and feedback-regulated nature of mechanosensitive signaling: proteins regulated by competing kinases and phosphatases whose activities peak at different stiffness thresholds naturally produce biphasic net phosphorylation profiles. *CTTN* and Vimentin are substrates of multiple kinases with opposing effects on cytoskeletal dynamics (43–45), while *EP300* and *TP53* integrate signals from several converging pathways through complex multisite phosphorylation (46, 47). These nonmonotonic patterns represent an underexplored feature of the mechanosensing phosphoproteome whose full biological significance warrants future investigation (48).

The identification of *NFATC4* as a mechanosensitive transcriptional regulator emerged from a stringent 2-step filtering strategy: first, selecting the 112 phosphosites with known regulatory functions from the 1,631 ANOVA significant sites; then, selecting those within the transcription regulation category, focusing exclusively on factors carrying phosphorylation at residues with documented roles in controlling transcriptional activity. We validated elevated regulon activities in IPF fibroblasts compared with healthy controls in the pySCENIC analysis of the multicohort atlas. The bootstrap comparison confirmed that any combination of 3 factors from this mechanosensitive set showed greater enrichment in IPF fibroblasts than randomly selected triplets, providing statistical evidence that the in vitro phosphoproteomic predictions are operationally relevant in human disease. Among the 6 candidates, *NFATC4* stood out as the most compelling: the prior knowledge database suggests it regulated the 3 most highly upregulated genes in IPF fibroblasts and 119 significantly induced targets, including established mediators of fibroblast activation (*IGFBP3*, *GLI1*), ECM remodeling and collagen cross-linking (*POSTN*, *COMP*, *PLOD2*, *COL18A1*, *COL6A2*), and myofibroblast-associated matrix turnover (*LTBP3*, *MMP2*, *MRC2*, *AEBP1*). The convergence of unbiased phosphoproteomic prediction with independent in vivo transcriptomic validation provides strong evidence that *NFATC4* is not merely a correlate of fibrotic disease but an active driver of the mechanosensitive transcriptional program in IPF fibroblasts.

The transition from physiological to pathological ECM stiffness is a critical and underappreciated driver of fibrosis progression. In the healthy lung, parenchymal stiffness is approximately 1–5 kPa, as measured by atomic force microscopy microindentation (49, 50), well below the mechanosignaling threshold we identified for fibroblast activation. During fibrosis, progressive ECM remodeling and collagen cross-linking stiffen the interstitium to 10–50 kPa in established fibrotic regions (8, 51), and ECM microstructure changes have been detected at the leading edge of fibrotic lesions before overt matrix deposition occurs, suggesting that mechanical alterations precede and may drive disease spread (52). As fibroblasts respond to this early stiffening by increasing collagen secretion and contractility, processes we show here to be dependent on *NFATC4* phosphorylation, ECM stiffness increases further, creating a self-reinforcing mechanosensing loop documented in multiple fibrotic contexts (53). The progressive colocalization of *NFATC4*, *ACTA2*, and *CTHRC1* across mild, moderate, and severe IPF lung tissue sections is consistent with this model, suggesting that *NFATC4*-mediated mechanosensing becomes increasingly operative as tissue stiffness accumulates with disease severity. Notably, soft matrices of physiological stiffness (~1 kPa) have been shown to deactivate IPF fibroblasts and reduce their contractility and proliferative rate (53), demonstrating that the mechanical microenvironment is a primary determinant of fibroblast activation state independently of soluble signals. At this stage, stiff ECM itself becomes the primary disease-propagating signal, independently of the initial injurious stimulus (52).

The mechanistic core of this study is the bidirectional phospho-switch proof. WT *NFATC4* overexpression at comparable protein levels had no effect on *ACTA2*, F-actin, or *CTHRC1* under any condition tested, establishing that *NFATC4* function is governed by posttranslational regulation rather than protein abundance, consistent with the well-established principle that *NFAT* TFs are tightly regulated at the level of phosphorylation-dependent nucleocytoplasmic shuttling (54, 55). The phosphomimetic mutant (S213D/S217D) bypassed the mechanical stiffness requirement entirely, driving robust myofibroblast differentiation on soft 0.5 kPa substrates in the complete absence of *TGFB*, a condition under which control cells show minimal activation. Conversely, the phospho-dead mutant (S213A/S217A) abolished all myofibroblast markers despite comparable protein expression, remained cytoplasmic, and could only weakly be rescued by *TGFB* stimulation on stiff substrates, which might be explained by remaining endogenous nonmutated *NFATC4* or additional compensatory mechanisms downstream of *TGFB* driving myofibroblast differentiation independently of calcineurin-*NFAT*, potentially through SMAD-dependent pathways (7, 56). The strong reduction of transcriptional activity in the phospho-dead mutant despite maximal upstream stimulation formally establishes S213/S217 phosphorylation as the critical regulatory switch controlling *NFATC4* nuclear localization and transcriptional output. Critically, all findings were fully reproduced in IPF-derived primary fibroblasts, which despite their constitutively activated fibrotic phenotype retained identical phosphorylation-dependent regulation, demonstrating mechanistic conservation between healthy and disease-derived fibroblasts. The convergence of *JNK* and calcineurin inhibition, and phospho-dead mutant phenotypes, together with the *NFATC4* siRNA data, confirms that the effects of *JNK* and calcineurin inhibition are specifically mediated through *NFATC4* rather than through parallel targets.

The Ca²^+^/calcineurin/*NFAT* axis has been implicated in mechanosensing in several cell types. Cell spreading on stiff substrates induces endoplasmic and nuclear calcium release (57), and the ability of cellular nuclei to sense mechanical forces is at least partially dependent on calcium store release as a downstream mediator (58). Our finding that calcineurin inhibition suppresses stiffness-induced myofibroblast differentiation in healthy fibroblasts is consistent with this paradigm. The nuclear morphology data from L-shaped micropatterns adds a further dimension: *NFATC4*-knockdown cells displayed larger, rounder, more relaxed nuclei with redistributed focal adhesions and F-actin, indicating that *NFATC4* regulates cytoskeletal tension and nuclear mechanotransduction (59), likely through transcriptional control of cytoskeletal effectors including myosin heavy chain genes (60–62). This feedback between *NFATC4* transcriptional activity and cytoskeletal tension may amplify the initial mechanosensing signal, contributing to the self-reinforcing nature of the myofibroblast state.

The role of *NFATC4* in fibroproliferative disease extends beyond the lung. In the heart, *NFATC4* activation downstream of calcineurin and syndecan-4 drives cardiac fibroblast-to-myofibroblast differentiation, upregulating collagen type III and MRTF-A in response to mechanical pressure overload (26). In the liver, RCAN1-mediated inhibition of calcineurin suppresses *NFATC4* nuclear translocation and alleviates fibrosis in the CCl4 model (63), providing independent evidence that *NFATC4* is a profibrotic effector downstream of calcineurin in mesenchymal cells. In the kidney, *NFAT*/calcineurin signaling has been similarly implicated in renal tubular fibrosis and ECM deposition (64). A recent spatial transcriptomic study of interstitial lung diseases identified *NFATC4* transcriptional activity as enriched in *CTHRC1*^hi^ myofibroblasts and showed that *NFATC4* knockdown attenuates *TGFB*- and stiffness-induced fibroblast activation (27), providing correlative evidence in human tissue that complements our mechanistic site-specific mutant data. Collectively, these findings across cardiac, hepatic, renal, and pulmonary fibrosis establish *NFATC4* as a broadly conserved profibrotic effector in fibroproliferative diseases of mesenchymal origin and suggest that the *JNK*-*NFATC4* phospho-switch identified here may represent a conserved mechanosensing node across organ systems.

The identification of *CTHRC1* as a downstream target of phosphorylated *NFATC4* is particularly noteworthy. *CTHRC1* has been established as a defining marker of the pathological *CTHRC1*^+^/*ACTA2*^+^ myofibroblast population that drives fibrosis progression across lung, heart, and liver in multiple single-cell studies (16–20), and its expression is absent in healthy lung parenchyma. The progressive colocalization of *NFATC4*, *CTHRC1*, and *ACTA2* in fibrotic foci of increasing disease severity in human IPF tissue positions *NFATC4* as an upstream regulator of this pathological cell state. Whether *CTHRC1* is a direct transcriptional target of *NFATC4*, through *NFAT* binding sites in its promoter, or is induced indirectly through *NFATC4*-dependent cytoskeletal and ECM remodeling programs remains to be determined and represents an important question for future work.

We acknowledge several limitations of this study. The kinase upstream of *NFATC4* S213/S217 is identified as *JNK* based on pharmacological inhibition and prior work (24, 25), but direct phosphorylation of these sites by *JNK* in lung fibroblasts was not demonstrated by kinase assay or phospho-specific antibody after *JNK* activation. The heterogeneity of mechanosignaling between CCL-151 and pHLFs was substantial, reflecting cell-intrinsic differences in mechanosensing thresholds that may be relevant to the diversity of fibroblast responses observed in vivo. However, the stiffness threshold of 2–8 kPa was highly consistent across both cell systems as the major activation boundary, with several signaling events already emerging at 2 kPa. The functional experiments were performed in 2D culture systems; whether the phospho-switch operates equivalently in 3D tissue environments and in vivo remains to be established. Finally, while the pharmacological data with FK-506, CsA, and *JNK* inhibitor VIII provide proof of concept for therapeutic targeting of the *NFATC4* pathway, validation in established preclinical fibrosis models is required to assess therapeutic efficacy in vivo.

In summary, we provide a proteome-wide map of phosphorylation events during human lung fibroblast mechanosensing and identify the *NFATC4* S213/S217 phospho-switch as a decisive regulatory node linking ECM stiffness to myofibroblast identity. The necessity and sufficiency of this single phosphorylation event, demonstrated through bidirectional site-specific mutants in both healthy and IPF-derived fibroblasts, establishes a direct causal connection between mechanical signaling and the transcriptional commitment to a *CTHRC1*^+^ myofibroblast fate. The convergence of mechanical (*JNK*-dependent) and cytokine (*TGFB*/Smad2/3) inputs at this phospho-switch, and its conservation in disease-derived fibroblasts, positions *NFATC4* S213/S217 as a candidate therapeutic target for interrupting the self-reinforcing mechanosensing loop that drives fibrosis progression.

## Methods

### Sex as a biological variable.

Both male and female human lung fibroblasts were used; sex was not considered as a biological variable.

### Cell culture.

CCL-151 (ATCC, mycoplasma negative) was maintained in DMEM/F-12 (Thermo Fisher Scientific, 31330038) with 10% FBS (Sigma-Aldrich, S0615-500ML) and 1% penicillin/streptomycin (Thermo Fisher Scientific, 15140122). pHLFs were obtained from the CPC-M bioArchive at the Comprehensive Pneumology Center. pHLFs derived from peritumor control tissues and IPF patient–derived lung explants were cultured in DMEM/F12 medium with 20% FBS and 1% penicillin/streptomycin. All cells were cultivated at 37°C and 5% CO_2_ in a humidified atmosphere.

### Plasmid generation.

Human *NFATC4* coding sequence was PCR amplified from healthy lung cDNA (KOD Hot Start Polymerase, Sigma-Aldrich) using primers h*NFATC4*-for (5′-ccatgggggcggccagctgcgagg-3′) and h*NFATC4*-rev (5′-ttcaggcaggaggctcttctccagg-3′). The WT HA-tagged overexpression plasmid [p(A)-AAV_Cp-HA-hNFATC4_SFFVp-NESmCherry] was generated by In-Fusion cloning (Takara) into p(A)-AAV_Cp_SFFVp-NESmCherry (CMV promoter) using primers HA-h*NFATC4*-for (5′-TACGACGTGCCCTACGCCGGCGGAAGCGGCGGAGGGGCGGCCAGCTGCGAGG-3′) and h*NFATC4*-rev, with backbone amplified using h*NFATC4*-bb-for (5′-cctggagaagagcctcctgcctgataatagggagaccacaacgg-3′) and HA-h*NFATC4*-bb-rev (5′-CCGGCGTAGGGCACGTCGTAGGGGTAGCCCATGGTGGCGGTTAACAGGCC-3′). The phosphomimetic (S213D/S217D) plasmid was generated by primer-overhang mutagenesis using hNFTATC4(S213D/S217D)-for (5′-cctcccgctttggcctgggcGACccgctgcccGACccccgggcctcccctcggcc-3′) and h*NFATC4*-rev for the C-terminal fragment, and h*NFATC4*-bb-for and h*NFATC4*(206-212)-rev (5′-gcccaggccaaagcgggagg-3′) for the backbone/N-terminal fragment, followed by In-Fusion cloning. The phospho-dead (S213A/S217A) plasmid was generated analogously using h*NFATC4* (S213A/S217A)-for (5′-cctcccgctttggcctgggcgccccgctgcccgccccccgggcctcccctcggcc-3′). Further sequence details are available upon request.

### Western blot.

Cells were lysed in RIPA buffer (50 mM Tris-HCl pH 7.6, 150 mM NaCl, 1% NP-40, 0.1% SDS, 0.5% sodium deoxycholate) with protease (Roche) and phosphatase inhibitors (PhosStop, Roche) on ice for 30 min and centrifuged at 14,000*g*/10 min/4°C. Protein was quantified by BCA (Thermo Fisher Scientific). Samples (10–20 μg) in 6× Laemmli buffer (6% SDS, 300 mM Tris-HCl pH 6.8, 25% glycerol, 324 mM DTT, 0.1% bromophenol blue, 50 mM NaF, 100 mM β-glycerophosphate) were heated at 95°C/5 min, resolved on 10%–15% SDS-PAGE, and transferred to PVDF membranes (350 mA/2 h/ice). Membranes were blocked in Roti-Block (Roth) with primary antibodies overnight at 4°C and secondary antibodies for 1 h at room temperature (RT) (1:20,000). Chemiluminescence was detected by ChemiDoc (Bio-Rad), and bands were quantified in Image Lab as signal normalized to loading control. Antibodies were as follows: anti-*GAPDH* (1:1,000, Cell Signaling Technology, 5174S), anti–CK IIα (1:1,000, Abcam, ab70774), anti–p(S/T) DXE CK2 substrate (1:1,000, Cell Signaling Technology, 8738), anti-pMLC2 Thr18/Ser19 (1:2,000, Cell Signaling Technology, 3674), anti–p*FOXO3*a S7 (1:1,000, Cell Signaling Technology, 14724S), anti-pPaxillin S126 (1:1,000, Life Technologies, 441022G), anti-pCBX3 Ser93 (1:1,000, Cell Signaling Technology, 2600), anti-rabbit IgG-HRP (1:20,000, Cell Signaling Technology, 7074), and anti-mouse IgG-HRP (1:20,000, Cell Signaling Technology, 7074).

### Phosphoproteome sample preparation.

CytoSoft 6-well dishes (Advanced BioMatrix; 0.2, 0.5, 2, 8, 16, and 32 kPa) or plastic dishes were coated with fibronectin (10 μg/mL, 1 h, RT). 500,000 cells/well were seeded 120 min, then lysed in GdmCl buffer (6 M GdmCl, 100 mM Tris pH 8.5, 10 mM TCEP, 40 mM CAA), heated at 95°C/5 min, cooled on ice for 15 min, sonicated (Bioruptor, 10 × 30 s max), heated again, and diluted 1:2 with water. Proteins were acetone precipitated overnight (4× volume, −20°C), collected (4,000*g*/10 min/4°C), washed twice with 80% acetone, air-dried, and resuspended in TFE digestion buffer (10% TFE, 100 mM ammonium bicarbonate). They were then digested in Lys-C (1:100 w/w, 37°C/1 h/350*g*) and then trypsin overnight. Phosphopeptides were enriched with TiO_2_ beads (Titansphere 10 μm, GL Sciences, 5010-21315; 1:10 beads/protein) in 80% ACN/6% TFA (5 min/350*g*/40°C), washed 4 times with 60% ACN/1% TFA, eluted twice with 40% ACN/15% NH_4_OH onto C8 StageTips (CDS Empore), concentrated by SpeedVac (Thermo Fisher Scientific), and acidified with 10% TFA (65). Peptides were desalted on SDB-RPS StageTips (wash: 0.2% TFA; elution: 80% ACN/5% NH_4_OH), concentrated, and reconstituted in MS loading buffer (2% ACN, 0.3% TFA).

### Single-run MS/MS.

Peptides were separated on a 40 cm × 75 μm C18 column (1.9 μm ReproSil, 50°C; Dr. Maisch) on an EASY-nLC 1000 (Thermo Fisher Scientific) at 300 nL/min using 0.1% formic acid (A)/60% ACN + 0.1% formic acid (B): 5%–25% B over 85 or 180 min, then 25%–50% B over 35 or 60 min. Data were acquired on a Q Exactive Orbitrap: full scan (300–1,600 *m*/*z*, R = 60,000, target 3e6), up to 5 data-dependent high-energy collision dissociation MS/MS (target 1e5, fill 120 ms, isolation 1.6 *m*/*z*, NCE 25%, underfill 40%, R = 15,000). Dynamic exclusion (40 or 60 s) and apex trigger (4–7 s) were enabled.

### LC-MS/MS data processing.

MaxQuant v1.5.3.34/Andromeda was used for LC-MS/MS data processing with FDR < 0.01 (protein, peptide, modification) (66) and the following criteria: variable mods: Met oxidation, N-terminal acetylation, phospho(STY); fixed: carbamidomethyl (C); minimum peptide length: 7 aa; match between runs: 0.7 min. The UniProt human database was used (April 2018; 20,138 entries). The following were excluded: rigidity dataset replicate 1 at 0.5 kPa (low IDs, outlier), spreading dataset replicate 2 at 20 min, and replicate 4 at 30 min.

### TF activity inference in vivo.

TF activity was inferred with pySCENIC v0.12.1 (67) on a preannotated mesenchymal subset from a multicohort IPF atlas (37) using normalized expression and a CollecTRI gene regulatory network with univariate linear modeling (68). Activities were compared between healthy and interstitial lung disease (ILD)/IPF samples by Mann-Whitney *U* test; significance was determined by |logFC| ≥ 0.5, *P* < 0.01 and visualized with ggplot2 v3.5.1/ggrepel v0.9.5. Bootstrap analysis (1,000 iterations, 3-TF random subsets vs. 6 pSTY TFs: *EP300*, *FOXO3*, *NFATC4*, *RB1*, *TP53*, *YBX1*) compared mean absolute activity differences (ILD vs. healthy) by Kolmogorov-Smirnov and Mann-Whitney *U* tests and was visualized by kernel density estimation. Pseudobulk differential gene expression (DESeq2) on a stratified-subsampled dataset identified pSTY-TF targets significantly upregulated in ILD (logFC >1, *P* < 0.05), aggregated as a stiffness-associated gene signature. Overlaps were visualized with R package Venn v1.12 (https://github.com/schillerlab/2025_Laura_Mattner_Mechanosensing_study/blob/dc88bd881acde8ef1ede37d2ace6bec9f2dd516d/Mechanosensing_core_geneset_signature.ipynb; commit ID dc88bd881acde8ef1ede37d2ace6bec9f2dd516d).

### RNAi.

Fibroblasts (passages 7–10) were seeded on fibronectin*-*coated plates (10 μg/mL, Sigma-Aldrich, F1141-1MG) in antibiotic-free medium. Pooled silencer select siRNAs were as follows: *NFATC4* (s9484 + s9483), *YBX1* (s9732 + s9733), and negative control (4390843; all from Thermo Fisher Scientific). Transfection was performed with Lipofectamine RNAiMAX (Invitrogen, 13778100) in Opti-MEM (catalog 31985062): siRNA and RNAiMAX were diluted separately, combined, incubated for 20–30 min, and added to cells. Some experiments were retransfected at 48 h and cultured further for 24 h.

### Immunofluorescence.

Cells were seeded on fibronectin*-*coated coverslips or ibidi dishes (0.5/28 kPa) for 120 min. The samples were then washed 3 times with PBS, fixed with 4% PFA for 20 min at RT, permeabilized with 0.1% Triton X-100 for 10 min, and blocked with 5% BSA for 30 min at RT. Primary antibodies diluted in 1% BSA/PBS were incubated overnight at 4°C, followed by incubation with secondary antibodies for 2 h. Nuclei were stained with DAPI for 10 min, and samples were mounted using fluorescent mounting medium or maintained in PBS (69). The samples were imaged on a Zeiss LSM 710 or AXIO imager. Antibody and primer details are provided in Supplemental Table 4.

### ECM deposition.

384-well CellCarrier plates (PerkinElmer, 6007550) were coated with fibronectin (10 μg/mL, 1 h, RT). 6,000 cells/well were seeded in antibiotic-free medium. siRNA complexes were added overnight, and the medium was replaced with 1% FBS for 24 h of starvation, with or without *TGFB* (1 ng/mL) for 3 days. For staining, primary antibody mix was added directly (3 h, 37°C), then secondary antibodies + Hoechst 33342 (1 μg/mL, 1 h), washed 3 times in DPBS, and fixed with 4% PFA (30 min, 37°C).

### Collagen gel contraction.

1.5 × 10^5^ cells/mL was mixed with collagen type I with or without *TGFB* (1.5 ng/mL), neutralized with 0.5 M NaOH, dispensed (500 μL/well, 24-well plate), and polymerized for 20 min at 37°C. Medium with or without *TGFB* (1 ng/mL, 600 μL) was added, and gels were released with a pipette tip (70). Contraction was imaged using ChemiDoc at 12, 24, 48, 72, 96, and 120 h (71).

### Collagen type I invasion assay.

Rat tail collagen type I (Thermo Fisher Scientific, A1048301) was polymerized in 96-well plates (50 μL/well, 4 h, 37°C). 2 × 10^4^ cells were seeded on top, invaded for 96 h, fixed (4% PFA, 30 min), permeabilized (0.25% Triton X-100, overnight), stained with DAPI (1:1,000), AF647-Phalloidin (1:150, A22287, Thermo Fisher Scientific), anti–α-SMA (1:250) overnight at 4°C, and then donkey anti–mouse-568 (1:500, 1 h, 37°C). Z-stacks (50 images, 500 μm range) on LSM 710. 3D projections were performed in ImageJ (NIH). Nuclei were quantified by spot detection in Imaris v9.9.1, filtered by z-position.

### RT-qPCR.

RNA isolated with a Quick-RNA Microprep Kit (Zymo, R1050) and quantified with a Nanodrop (Thermo Fisher Scientific). cDNA from 100 ng RNA (GoScript RT, Promega, A5000; 42°C/1 h). qPCR was performed on a LightCycler 480 (GoTaq Master Mix, Promega, A6001) at 95°C for 2 min, 50 times (95°C 5 s/60°C 1 min). Expression was determined by 2^−ΔΔCt^ with *GAPDH* as reference. Primers are provided in Supplemental Table 4.

### Fibronectin micropattern experiments.

Cells were retransfected at 48 h, cultured 24 h further, dissociated with 50% TrypLE/DPBS, and washed in DMEM/F12 with 1% FBS. 25,000 cells were seeded on L-shaped fibronectin*-*micropatterned coverslips (5 μg/mL, 1 h, 37°C) in 6-well plates. After approximately 30 min attachment, the coverslips were washed to remove floating cells and incubated 150 min further at 37°C. The cells were fixed (4% PFA, 10 min, 37°C), permeabilized (0.2% Triton X-100, 10 min), blocked (2% BSA, 1 h), primary antibodies (anti-paxillin 1:300, BD Biosciences, 610051; anti-pMLC 1:150, Cell Signaling Technology, 3674S; 3 h, 37°C), secondary antibodies + phalloidin (1:150, Invitrogen #A22287, 1 h), stained with DAPI, and mounted. Nucleus area and roundness (= 4 × area/π × major axis²) were calculated in ImageJ (72).

### Bioinformatic and statistical analysis.

Using Perseus, phosphosites were filtered at localization probability > 0.75 and log_2_ transformed, and reverse hits were removed. GO, KEGG, Pfam, GSEA, CORUM, and PhosphoSitePlus annotations were added. Valid-value filter was ≥3/group (rigidity), with ≥4/group (spreading). The data were median-normalized, and missing values were imputed using a width of 0.3 and a downshift of 1.8. ANOVA was performed both with and without imputation, and the results were combined. Significant sites were *z* scored, hierarchically clustered, and assessed by PCA. Fisher’s exact test, 5% FDR, was used for annotation enrichment. The *z* scored phosphosite averages were uploaded to IPA for core phosphorylation analysis. Comparison analysis was extracted for canonical pathways, upstream regulators, and diseases and functions. Perseus cluster members were analyzed in Metascape by express analysis (*H*. *sapiens*); a combined pathway heatmap was generated. Perseus output was imported to Scanpy (73, 74) for clustering and visualization of CCL-151 stiffness, spreading, merged, and CCL-151/phLF combined datasets.

### Statistics.

Quantitative data are presented as mean ± SD. Pairwise comparisons were performed using 2-sample *z* tests (in vitro immunofluorescence, *n* = 3), 2-tailed Student’s *t* tests (siNC vs. si*NFATC4* comparisons and micropatterning experiments), or Kruskal-Wallis test followed by pairwise Mann-Whitney *U* tests with Bonferroni correction across all markers and severity groups (IPF severity analysis). For in vitro multicondition experiments, *z* test *P* values were corrected for multiple comparisons using the Bonferroni method. Phosphoproteomic hits were identified by 1-way ANOVA at 1% FDR in Perseus. A *P* value of less than 0.05 was considered statistically significant.

### Study approval.

The study was approved by the local ethics committee of the Ludwig-Maximilians University Munich (ethic vote 333-10). Written informed consent was obtained for all study participants.

### Data availability.

Proteome raw data and MaxQuant processing tables can be downloaded from the PRIDE repository (75) under accession number PXD063448. Values for all data points in graphs are reported in the Supporting Data Values file.

## Author contributions

HBS conceived the research narrative and experimental design and supervised the entire project. HBS, LFM, SK, and MMH wrote the manuscript. LFM and HBS performed proteomic experiments and analyzed the data. SK and ZZ performed *NFATC4* loss-of-function experiments and analyzed the data. MMH and SK generated point mutants and performed loss- and gain-of-function experiments. SK, ZZ, LFM, XW, SA, AAW, and MMH performed validation experiments. AKV and UC assisted with cell culture experiments. SRSP performed integrative analysis of TF regulons. LFM, HBS, CHM, SK, and MA performed bioinformatic analysis of phosphoproteomic data. NK, MGS, JB, WAW, and LJDS provided patient material and resources. JP generated adhesive micropatterns. AÖY, GB, MM, and HBS provided resources and funding. All authors read and approved the manuscript. Co–first authorship order was assigned by mutual agreement among the co–first authors and senior author, reflecting the relative volume of experimental contributions, with SK contributing primarily to functional validation and mechanistic experiments and LFM contributing primarily to phosphoproteomic data generation.

## Conflict of interest

The authors have declared that no conflict of interest exists.

## Funding support

- German Research Foundation (DFG) project number 324864813 (to HBS).
- German Center for Lung Research (DZL).
- Helmholtz Association.
- Max Planck Society.

## Supplementary Material

Supplemental data

Unedited blot and gel images

Supplemental table 1

Supplemental table 2

Supplemental table 3

Supplemental table 4

Supplemental table 5

Supporting data values

## Version 1 09/01/2026

Electronic publication
