# Supplementary material for: Mechanosensitive phosphorylation of NFATC4 at S213/S217 drives fibroblast-to-myofibroblast transition and fibrosis
Source: J Clin Invest. 2026 Sep 1;136(17):e195121. doi: 10.1172/JCI195121 (PMC13528934; doi:10.1172/JCI195121)
Supplement: Supplementary file 1 [file jci-136-195121-s116.pdf]

Supplementary results text

Phosphoproteomics in primary human lung fibroblasts

To validate some of our observations in freshly isolated primary human lung fibroblasts (pHLF), we isolated pHLF from non-involved peritumor human lung tissue and repeated the phosphoproteome analysis in the same way as done for CCL-151 cells (Figure 1). Similar to CCL-151 the pHLF were spreading more on stiff substrates (Figure S4A). In pHLF we detected 7191 class-I phosphosites with a localization probability > 0.75. Principal component analysis showed a clear and reproducible separation of the distinct substrate rigidities (Figure S4B). The depth of pLFH phosphoproteomes was lower as in CCL-151 with between 2000 and 4000 quantified sites per replicate (Figure S4C-F). After filtering for sites that were at least measured four times in one of the stiffness conditions, 4725 sites remained for further analysis. Similar to CCL-151 (Figure 1) we found increased phosphorylation of MYL9 T19/S20 (Figure S4G) and PXN S126 (Figure S4H) when pHLF were cultured on stiff substrates. After filtering for significant sites, we identified 290 shared mechanosensitive phosphorylation sites in CCL-151 and pHLF (ANOVA significant with FDR <1%). In order to visualize the overlap in the kinetics across both experiments we embedded the PCA space of phosphosite intensities in one integrated UMAP plot. Using the louvain clustering algorithm we determined 7 distinct clusters of phosphosites in this integrated dataset (Figure S5A).

In particular louvain cluster 1 and cluster 4 displayed comparable kinetic profiles between CCL-151 and pHLF (Figure S5B) and thus represent highly conserved mechanosensitive phosphorylation sites (Figure S5C), such as ABLIM3 S503/504 (Figure S5D), which plays a role in regulation of serum-response factor (SRF)-dependent transcription (65). We subjected sites in Louvain cluster-1 and -4 to Metascape based pathway enrichment analysis and found that cluster-1 was enriched for GO terms such as 'actin cytoskeleton organization' and 'rRNA metabolic process', as well as the reactome pathway RHO GTPases activate Citron kinase. Cluster-4 showed significant enrichment for the GO terms 'actin cytoskeleton organization', 'histone modification' and 'regulation of cell morphogenesis', as well as the reactome pathway 'RHO GTPases activate PAKs'. Furthermore, cluster-4 was enriched in the DisGenNET terms 'fibromuscular dysplasia' and 'lung diseases'.

In summary, we have identified candidate signaling pathways and kinases involved in feedback connections of lung fibroblasts sensing the stiffness of their ECM environment.

Time-resolved analysis of lung fibroblast mechanosensing

When cells first adhere to the ECM substrate they undergo a process of cell spreading, which for CCL-151 lung fibroblasts on stiff substrates was isotropic in the first 30-60 minutes, followed by cell polarization and formation of large focal adhesions and stress fibers within the next hour (Figure S6A, B). Increased cell contractility over time was associated with increased phosphorylation of the myosin light chain on MYL9 T19/S20 (Figure S6B). To globally reconstruct cellular signaling during cell spreading and generate a time-resolved map of mechanosensitive phosphosites we measured phosphoproteomes at 10, 20, 30, 60, 90, and 120 minutes after seeding on FN coated stiff substrates (Figure S6C). A total of 12364 class-I phosphosites were detected (Table S3). PCA analysis showed good agreement between experimental replicates and a clear separation of timepoints (Figure S6D). We detected 42.8 % singly, 44.1 % doubly and 13.1 % triply phosphorylated peptides, with serine being the most phosphorylated residue, followed by threonine and only few tyrosine phosphorylations (Figure S7A).

The data set contains 689 regulatory phosphosites with a known function, and 883 sites that were not covered in the PhosphositePlus database (Figure S7B). The average phosphoproteome depth per replicate sample was 7224 (Figure S7C). Looking into two mechanosensitive phosphosites validated in the first experiment we found gradually increased PXN S126 and MYL9 T19/S20 over time (Figure S7D, E). ANOVA analysis (1 % FDR) resulted in 1265 significantly regulated phosphosites (Figure S7F, Table S4), which comprised two main clusters, representing the early phase of cell attachment (10-30 min) and the polarized contractile phase (60-120 min) respectively (Figure S6D and S7F). This broad categorization was refined by hierarchical clustering of the sites which resulted in eleven different cluster trends (Figure S6E). To highlight potential functional connections between these sites we mapped them according to their cluster identity on distinct signaling pathways (Figure S6F), illustrating relevant sections of the pathways ERK/MAPK signaling, actin cytoskeleton signaling, integrin signaling, phospholipase C signaling, Paxillin signaling, signaling by Rho GTPases signaling, HIF1a signaling, and eiF4 and p70S6K signaling (Figure S6F). We used Metascape and Ingenuity Pathway Analysis (IPA) for prediction of regulated pathways and upstream regulators (Figure S7G-I). Out of the 1265 significant sites, we found 90 sites with known regulatory functions in cell adhesion, cytoskeletal reorganization, cell motility, cell growth and transcription (Figure S8A-E).

To integrate the stiffness sensing and cell spreading time-course experiments, the identity of significantly regulated sites across the two datasets was compared. We identified 227 phosphorylation sites that were significantly regulated in both data sets (Figure S9A; Table S5), and visualized the overlap in a shared UMAP embedding (Figure S9C). Hierarchical clustering analysis (Pearson correlation) of this integrated dataset revealed three clusters with distinct regulation (Figure S8F). In cluster A we found 42 mechanosensitive sites that were increased on stiff substrates and peaked late in the cell attachment time-course (Figure S8G). Interestingly these sites were enriched for cytoskeletal processes on both actin and microtubule filament networks as well as the regulation of histone modifications and the 'CDC5L protein complex', which is essential for pre-mRNA splicing (Figure S8H). In cluster B we identified 61 phosphorylation sites belonging to 38 proteins, which are enriched for GO terms such as 'RNA splicing' and 'regulation of mRNA processing' as top enriched annotations (Figure S8G, H). Overall, the mechanosensitive sites in this cluster were increased on stiff substrates but in contrast to cluster A already peaked at 20–30 minutes after cell attachment. Cluster C contained 63 phosphosites corresponding to 34 different proteins. Here the increase in phosphorylation was already observed above 2 kPa and also the phosphorylation status of the corresponding sites in the spreading time course was continually increasing over time.

In summary, we used time-resolved phosphoproteomics of cell spreading on a stiff fibronectin substrate to analyze the temporal dynamics of mechanosensitive signaling events.

Supplementary figures

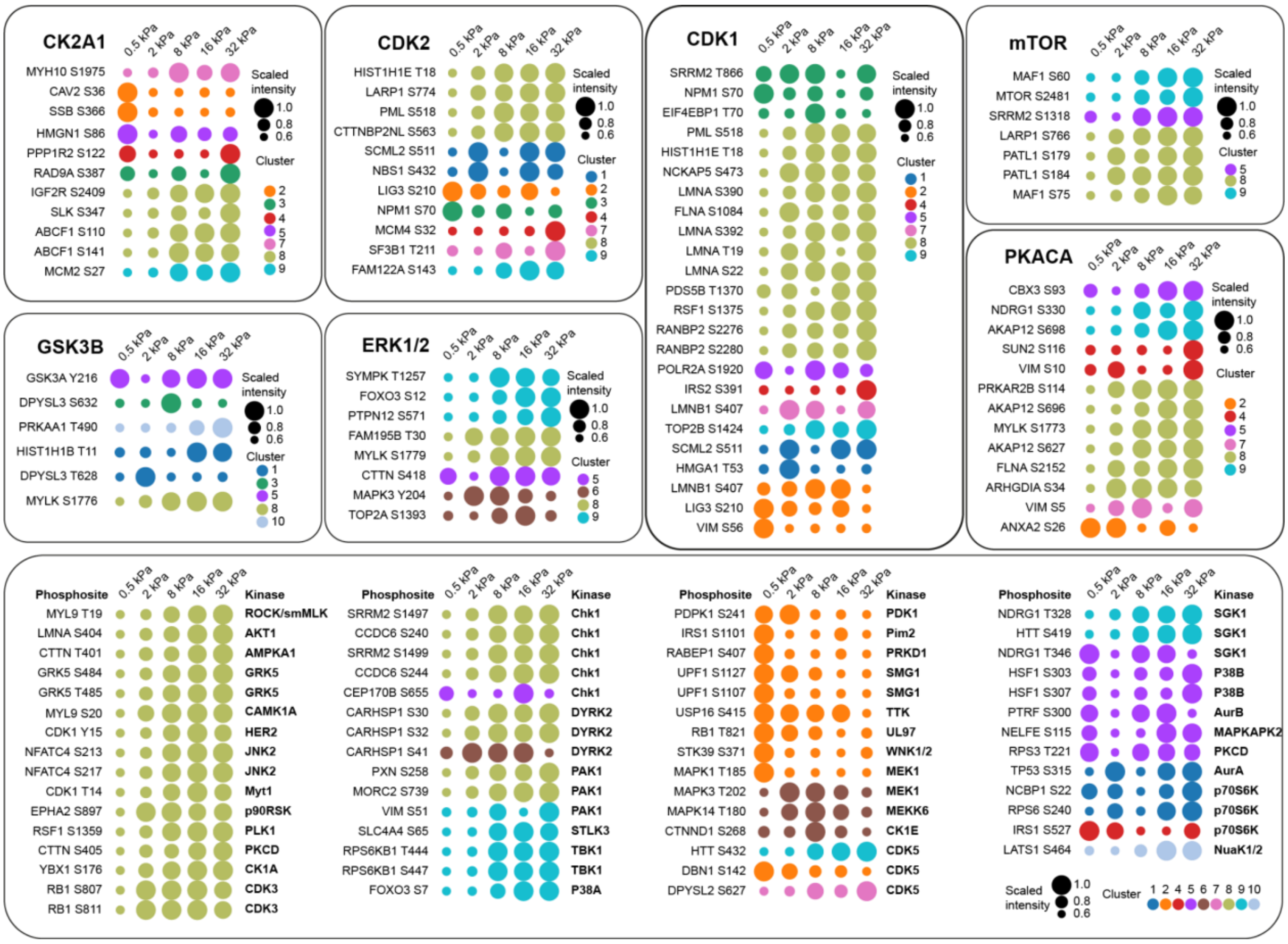

Figure S1. Predicted upstream kinases of mechanosensitive phosphosites.

Dot plots show the dynamics of scaled phosphorylation intensities on soft to stiff substrates for selected phosphosites with known regulatory site processes (PhosphositePlus database) associated with the indicated functional categories. Cluster identities from main Figure 2 are indicated by colors. The predicted upstream kinases for the regulated motifs are shown.

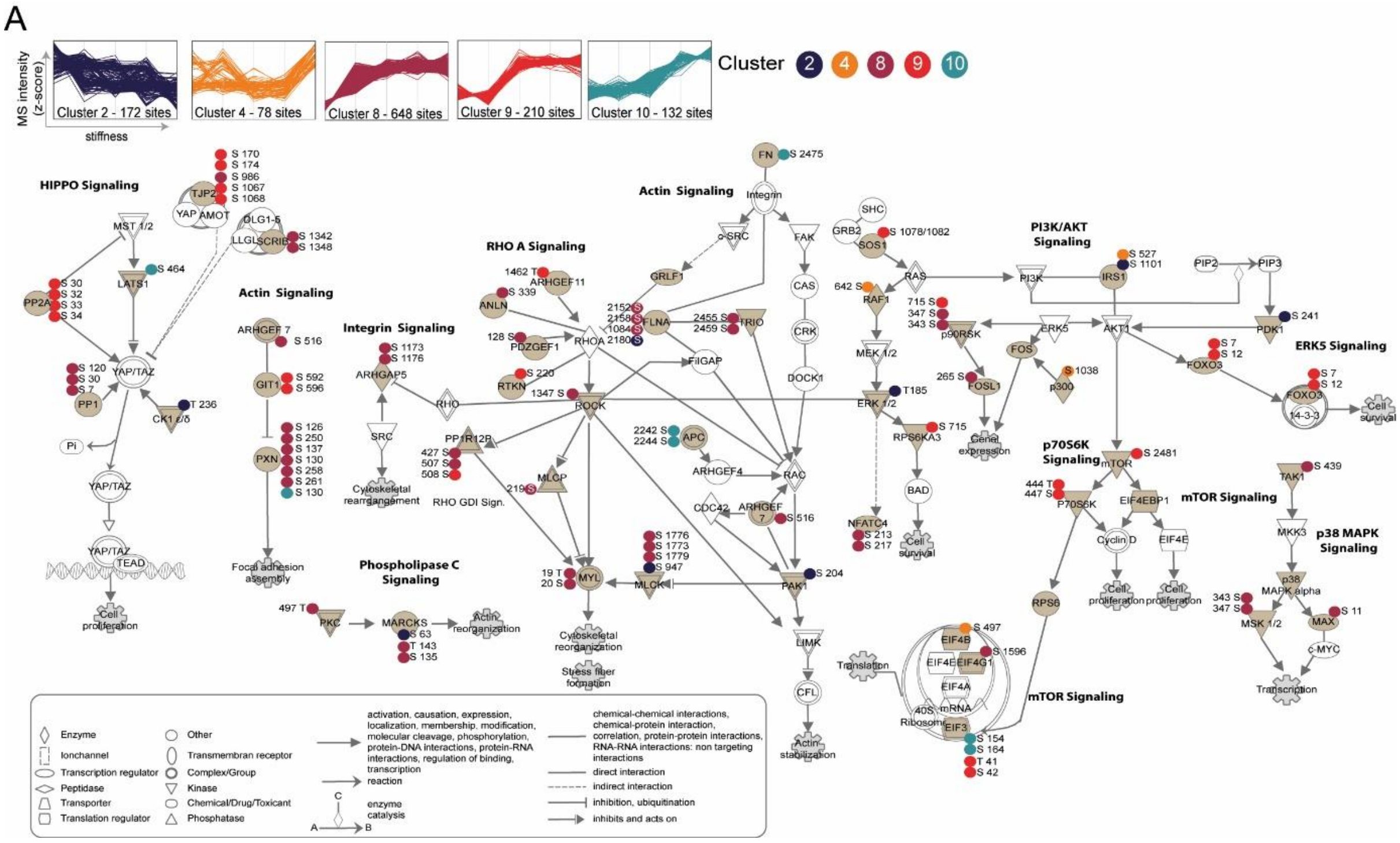

Figure S2. Stiffness-regulated phosphoproteome clusters map onto canonical mechanosensitive signaling pathways. (A) Line plots illustrating cluster-specific rigidity responses (cluster 2: blue; 4: orange; 8: burgundy; 9: red; 10: turquoise), alongside a pathway map of stiffness-regulated phosphosites within canonical signaling networks; light brown nodes indicate proteins with ≥1 significantly regulated phosphosite; white nodes indicate undetected or non-significant phosphosites.

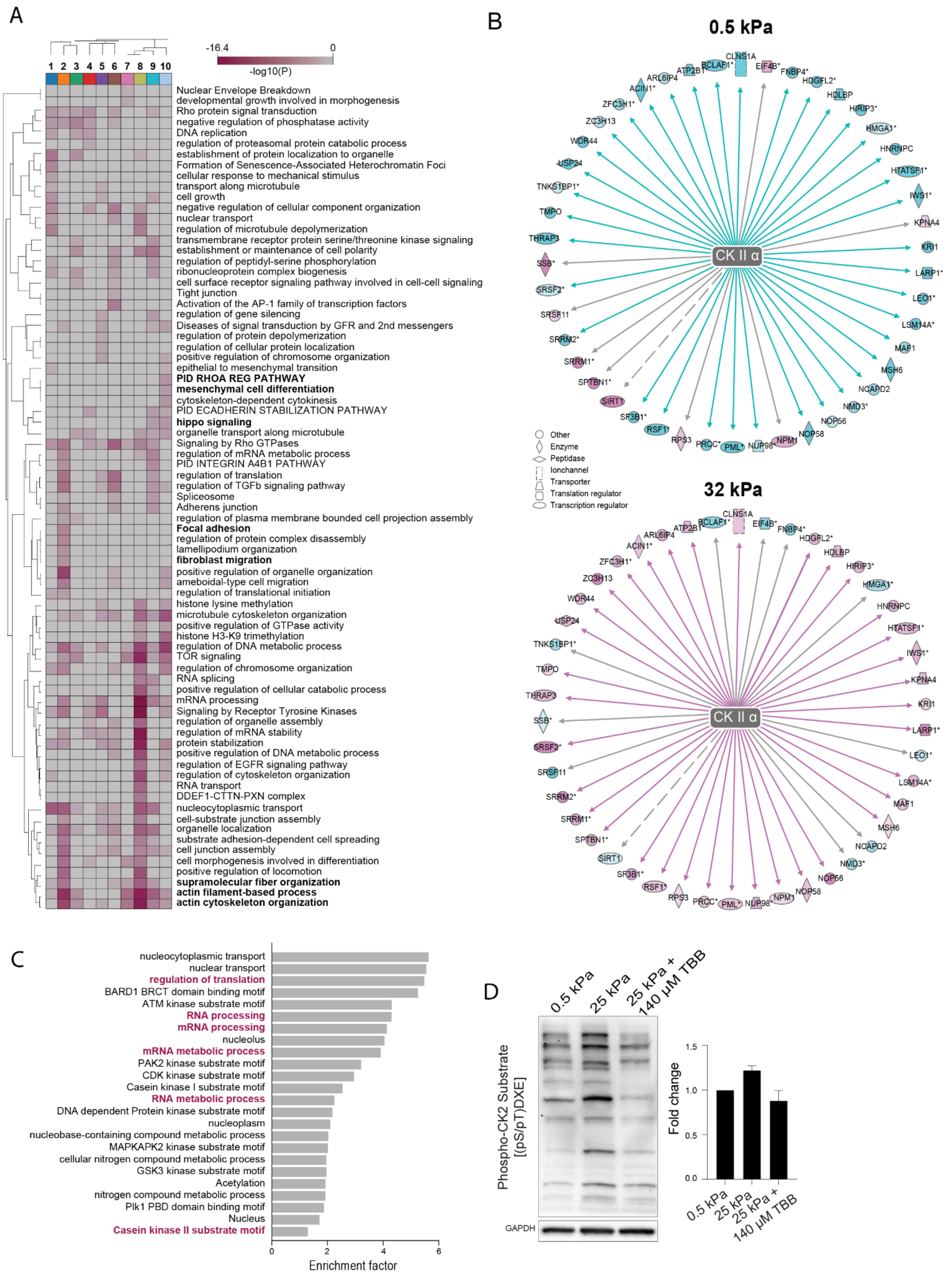

Figure S3. Pathway and upstream regulator analysis reveals stiffness regulated processes. (A) Heat map shows enriched terms across gene lists of clusters 1–10 in main Figure 2, colored by p-values. Enrichment analysis was performed with Metascape (www.metascape.org). **(B)** The graphs show CK2A1 targets predicted by Ingenuity Pathway Analysis (IPA) and their phosphorylation status in the phosphoproteomic data set. Turquoise color shades indicate low phosphorylation and pink color shades indicate high phosphorylation. Doted lines indicate an indirect interaction and continuous lines specify a direct interaction from prior knowledge databases. **(C)** Results of pathway enrichment analysis of the IPA CK2A1 target sites using Fisher exact test (FDR < 5%). **(D)**

Immunoblot probed with an antibody specific for proteins containing a pS/pTDXE motif, which represents a CK2 phosphorylation consensus sequence. GAPDH served as a loading control (n=3).

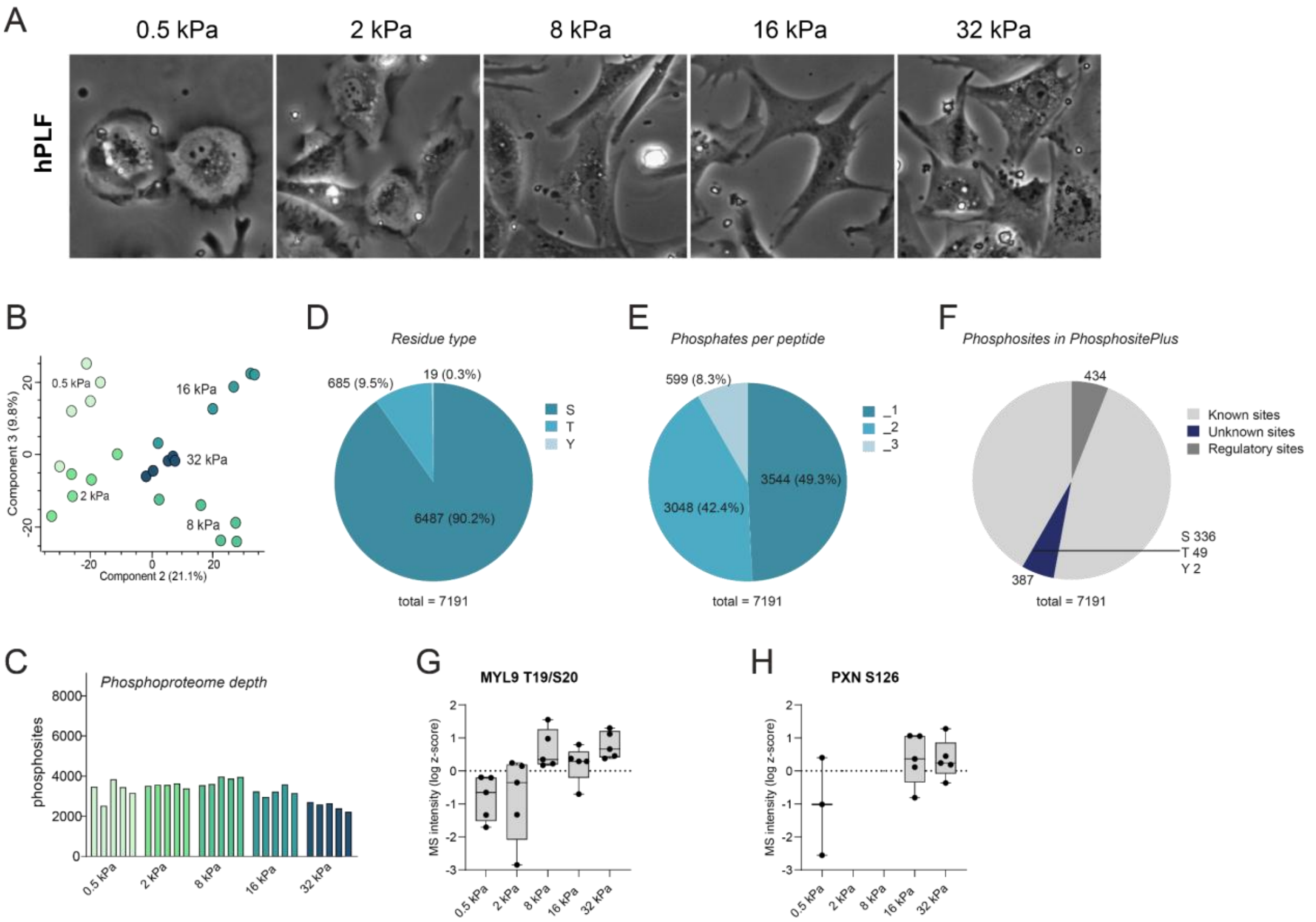

Figure S4. Human primary lung fibroblasts activate mechanosensitive signaling pathways. (A) Phase contrast images show primary human lung fibroblasts (pHLF) from peritumoral resection of a non-CLD patient seeded on fibronectin coated substrates with the indicated stiffness. **(B)** Principal component analysis (PCA) of the stiffness regulated phosphoproteome in pHLF. **(C)** The bar graph shows the number of quantified phosphosites in the indicated experimental conditions. **(D)** Pie chart represents the percentage of identified phosphorylated serines (S), threonines (T) and tyrosines (Y) residues. **(E)** The percentage of identified singly, doubly and triply (or more) phosphorylated peptides. **(F)** The number of already known sites, previously unreported sites and known sites with an assigned regulatory function in the PhosphoSitePlus database (www.phosphosite.org). **(G)** The box plot shows phosphopeptide intensities of MYL9 T19/S20 at the indicated experimental conditions in pHLF. **(H)** The box plot shows phosphopeptide intensities of PXN S126 at the indicated experimental conditions in pHLF.

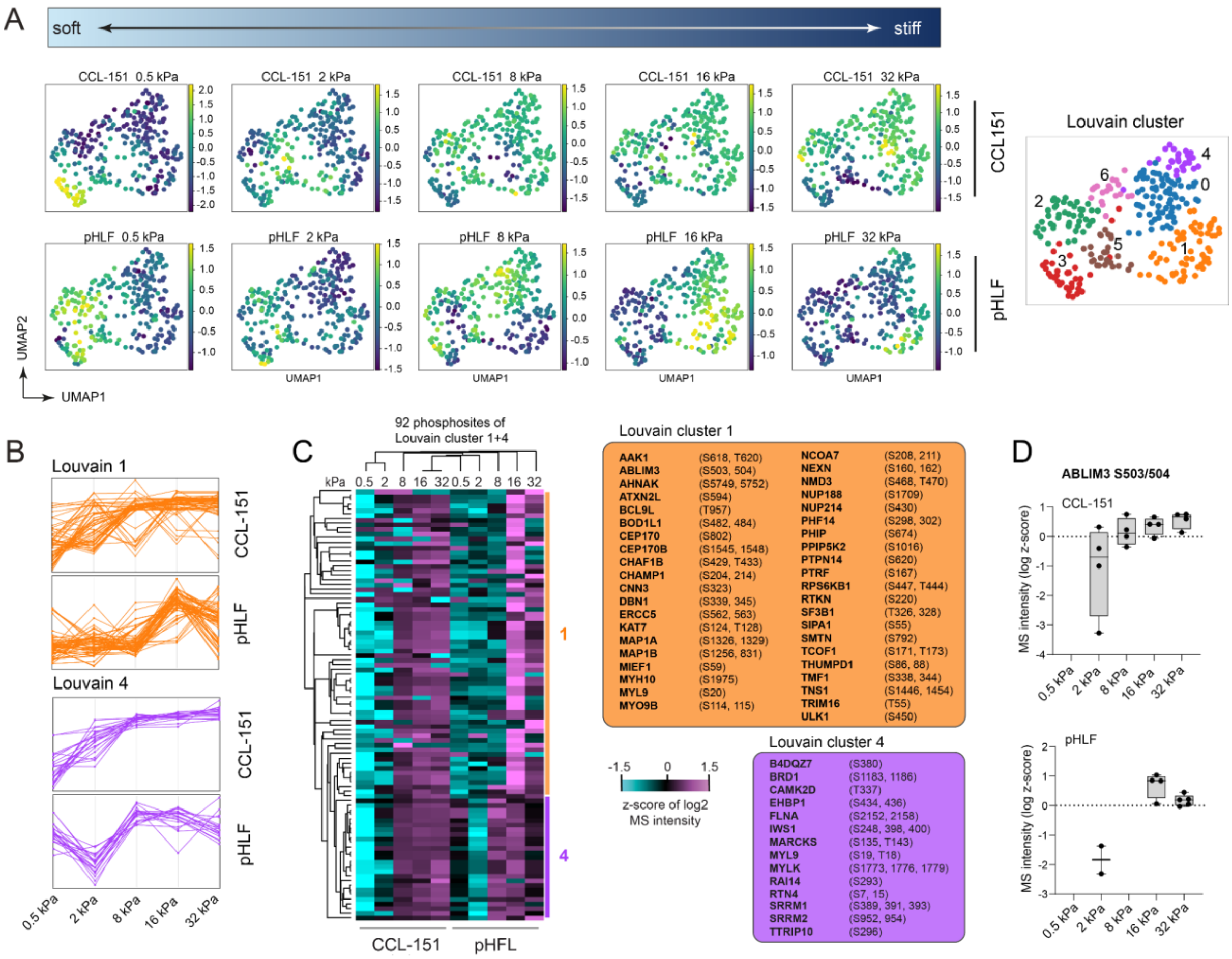

Figure S5. Integrative analysis of phosphoproteomes reveals conserved mechanosensitive sites in primary human lung fibroblasts. (A) The UMAP shows integrated phosphosites from CCL-151 and pHLF experiments. Each dot represents one unique phosphorylation site. The color code displays phosphorylation intensities in the indicated experimental conditions. In the right panel 7 clusters of phosphosites calculated by the louvain algorithm are shown. **(B)** The line plots illustrate the z-scored kinetic profiles of phosphosites in louvain cluster 1 and 4 for the two experiments. **(C)** Heatmap depicts hierarchical clustering (Pearson correlation) of the z-scored MS intensities across the indicated conditions. **(D)** The box plot shows phosphopeptide intensities of ABLIM3 S503/504 across the indicated experimental conditions.

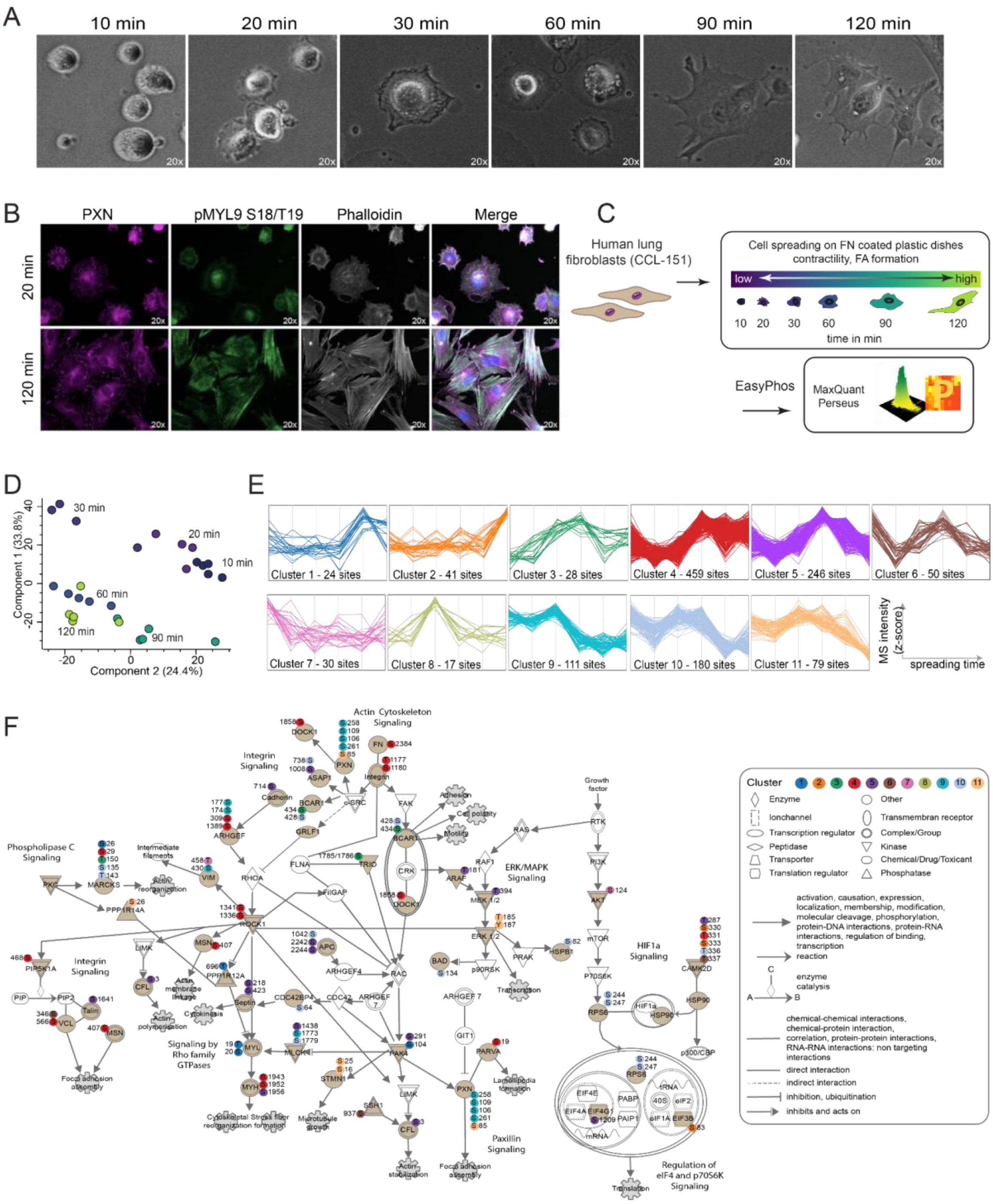

Figure S6. Time resolved analysis of phosphoproteome dynamics reveals distinct phases of cell spreading. (A) Phase contrast images show representative CCL-151 cells seeded on fibronectin-coated glass coverslips at the indicated timepoints. **(B)** Immunostainings show CCL-151 cells after spreading for 20 or 120 minutes. Staining of PXN in magenta, phosphorylated MYL9 (Thr19/Ser20) in green and actin (Phalloidin) in white. **(C)** Experimental design. **(D)** PCA of the regulated phosphoproteome during cell spreading. The two first components separate the data according to spreading time. **(E)** The 11 clusters with distinct activation profiles were defined by hierarchical clustering (Pearson correlation). **(F)** Mapping of a subset of regulated phosphosites during cell spreading on canonical signaling networks. The phosphorylation sites are color-coded according to their cluster identities shown in (E). Proteins color coded in light brown are contained in the phosphoproteomic data set. The white colored proteins were either not found to be phosphorylated in the data set or measured but were excluded by the settings of the performed ANOVA test.

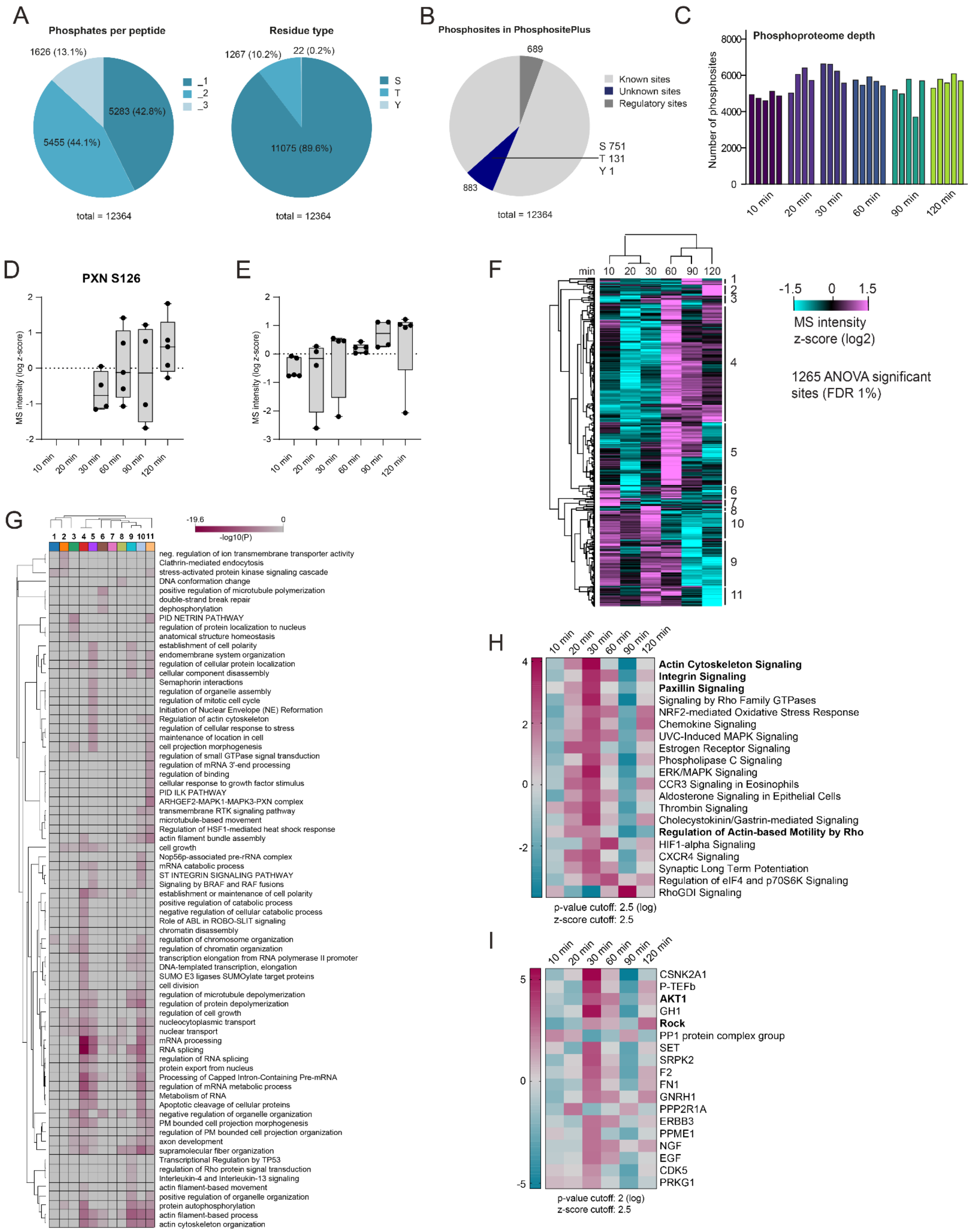

Figure S7. Distinct phases of cell signaling during fibroblast cell spreading on stiff substrates. (A) Left panel: pie chart presenting the percentage of identified singly, doubly and triply (or more) phosphorylated peptides. Right panel: pie chart presenting the percentage of identified phosphorylated serines (S), threonines (T) and tyrosines (Y) residues. **(B)** Pie chart showing the number of already known sites, previously unreported sites and known sites with an assigned regulatory function in the PhosphoSitePlus database. **(C)** The bar graph shows the number of quantified phosphosites in the indicated experimental conditions. **(D)** Phosphorylation of PXN at serine residue 126 is increasing during cell spreading on stiff substrates. **(E)** Phosphorylation of MYL9 on threonine residue 19 and serine residue 20 is increasing with spreading time on stiff substrates. **(F)** Heatmap shows hierarchical clustering (pearson correlation) of z-scored phosphosite intensities across the indicated time points. **(G)** Heat map shows hierarchical clustering of -log10 p-values of enriched terms for the clusters in (F). Enrichment analysis

was performed with Metascape (www.metascape.org). **(H, I)**. Phosphoproteomic data was analyzed using the Ingenuity pathway Analysis (IPA) platform. Significance of term enrichment is indicated through shading as log p-value. Regulated pathways belonging to "Canonical pathways" **(H)** and predicted "Upstream regulators" **(I)**.

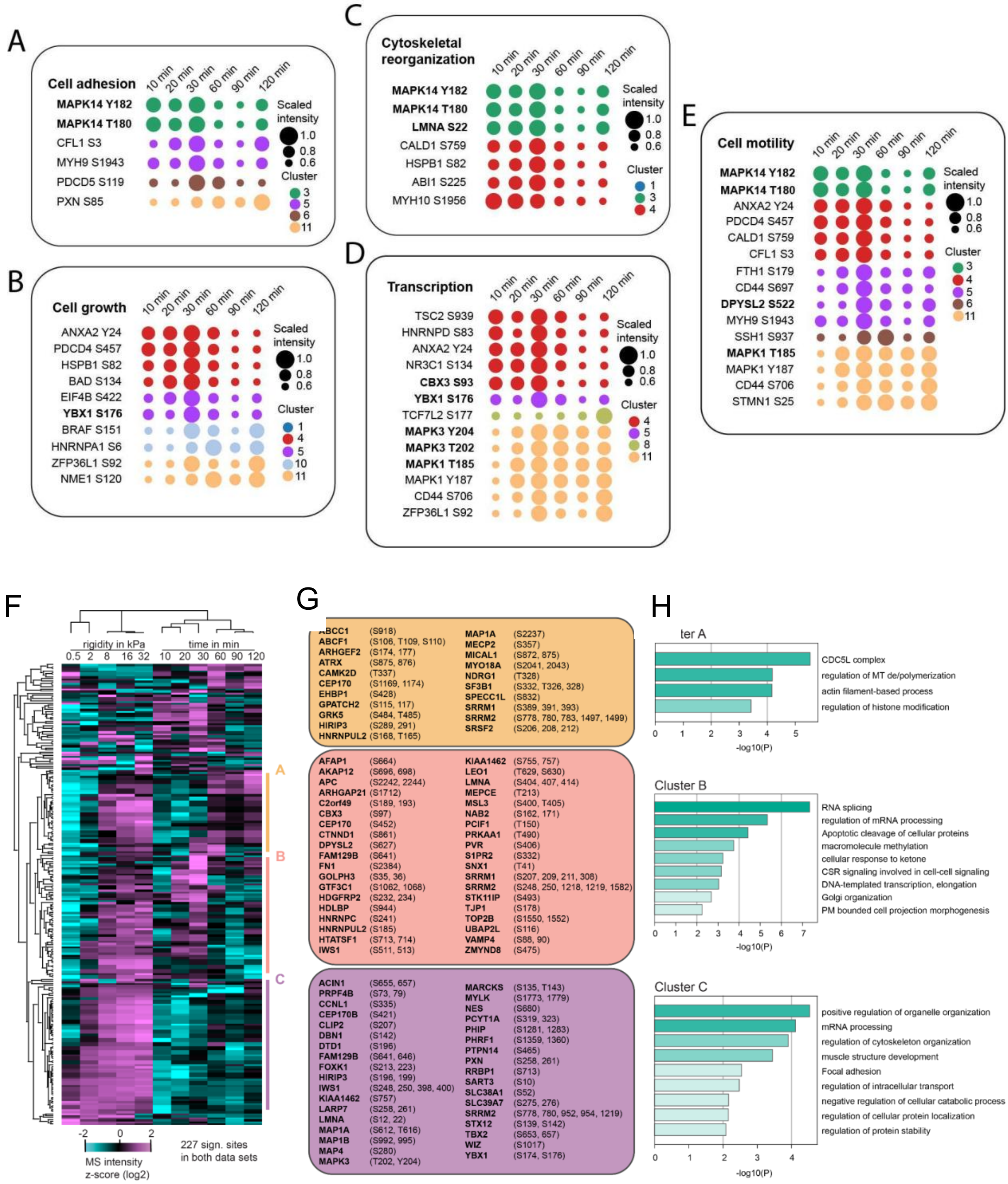

Figure S8. Kinetics of mechanosensitive sites after cell attachment. (A–E) Dot plots showing the dynamics of scaled phosphorylation intensities over spreading time of selected phosphosites associated with the regulatory sites process **(A)** 'Cell adhesion', **(B)** 'Cell growth', **(C)** 'Cytoskeletal reorganization', **(D)** 'Transcription' and **(E)** 'Cell motility. Mechanosensitive sites are highlighted in bold font. Dots are color-codes per cluster as presented in Figure S5. **(F)** Heatmap depicts hierarchical clustering (Pearson correlation) of the z-scored MS intensities across the indicated conditions. **(G)** Corresponding proteins to the regulated phosphosites with their respective phosphorylated sites in the clusters A, B and C. **(H)** Bar graph shows enriched terms across gene lists of clusters A, B and C, colored by -log10 p-values. Enrichment analysis was performed with Metascape (www.metascape.org).

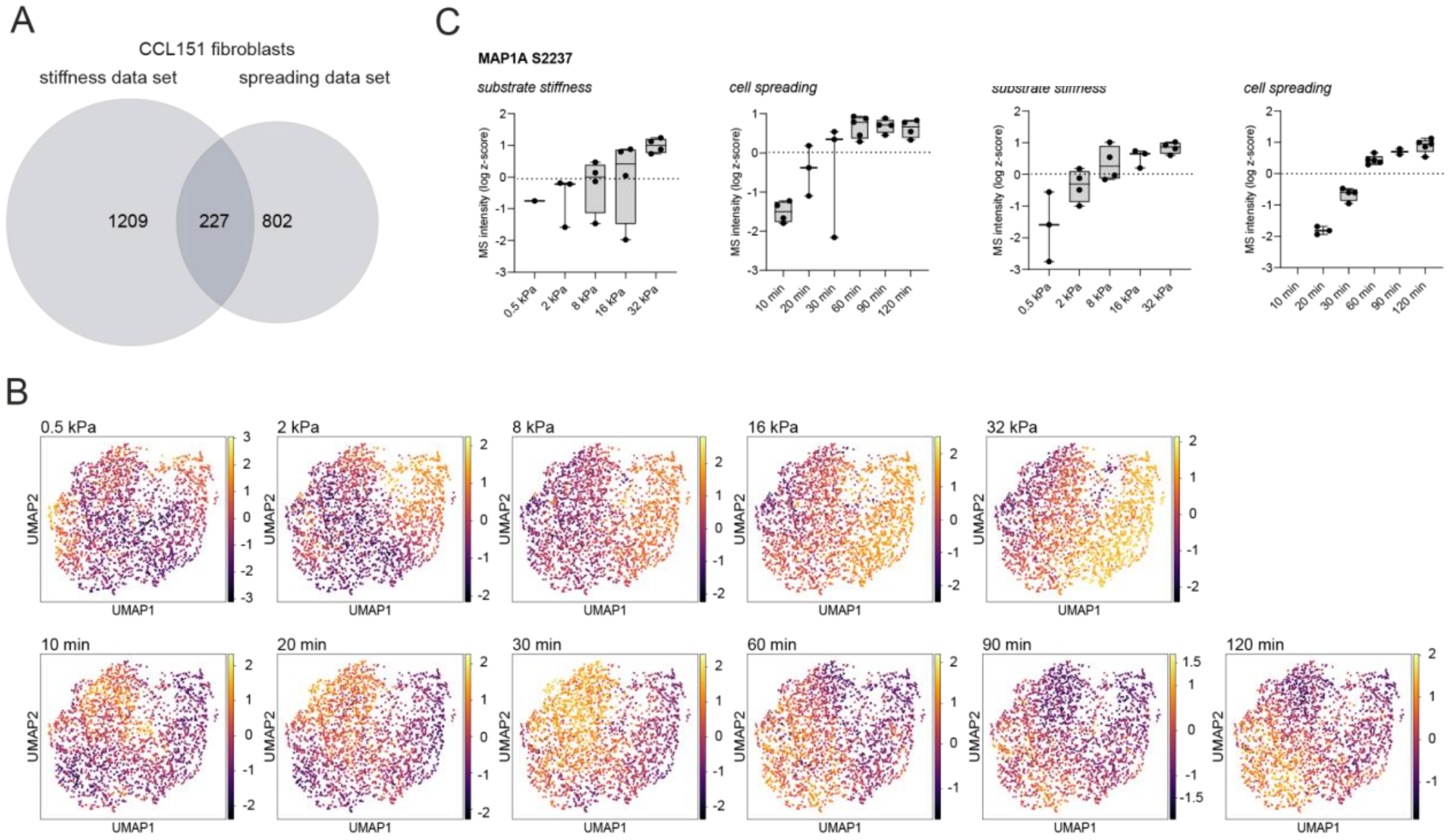

Figure S9. Course of phosphorylation and data integration to assess time-resolved aspects of stiffness sensing. (A) Venn diagram showing the overlap of significantly regulated phosphorylation sites in both stiffness and spreading datasets. **(B)** Box plot of phosphopeptide intensities of MAP1A S2237 and PXN S126 at the indicated experimental conditions. **(C)** The UMAPs for each substrate rigidity and spreading timepoint are color-coded according to the phosphorylation intensity of each site and visualize the course of phosphorylation across both datasets.

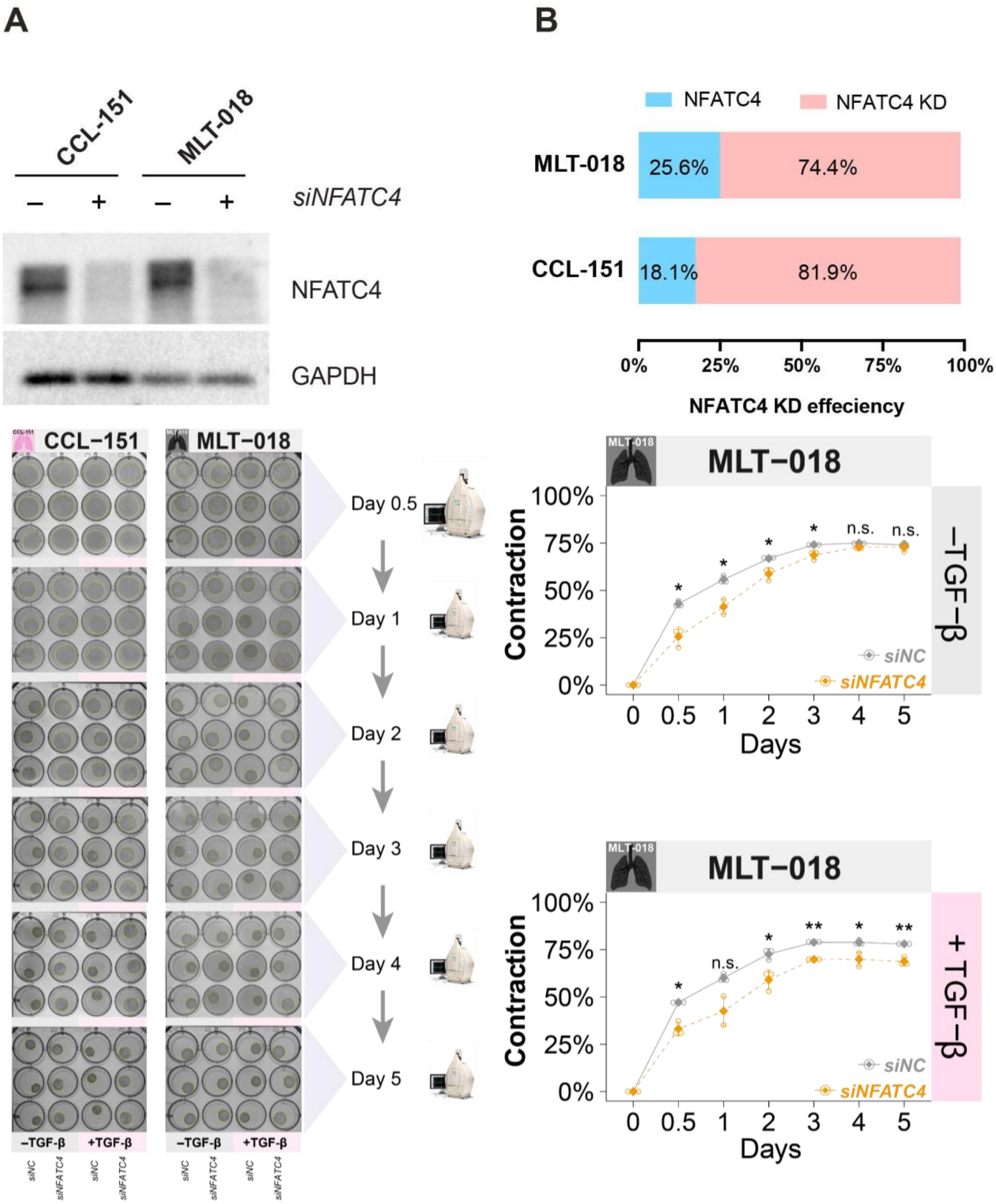

Figure S10. CCL-151 and MLT-018 induced-collagen gel contraction. (A) WB analysis of NFATC4 expression in two fibroblast cell lines. **(B)** Quantification of NFATC4 bands in A. GAPDH was used as a loading control for protein normalization. **(C)** Pictures of CCL-151 (left) and MLT-018 (right) induced-collagen I gel contraction on days 0.5, 1, 2, 3, 4, and 5, with the absence or presence of 1ng/mL TGF-β. The area of collagen pads was outlined by the yellow circle. Areas were accordingly measured with ImageJ. **(D)** MLT-018-induced collagen I contraction kinetics over time, with (upper panel) or without (lower panel) 1ng/mL TGF-β. Statistics: *p < 0.05, **p < 0.01, ***p < 0.001, ****p < 0.0001, and n.s.: not significant according to the t-test.

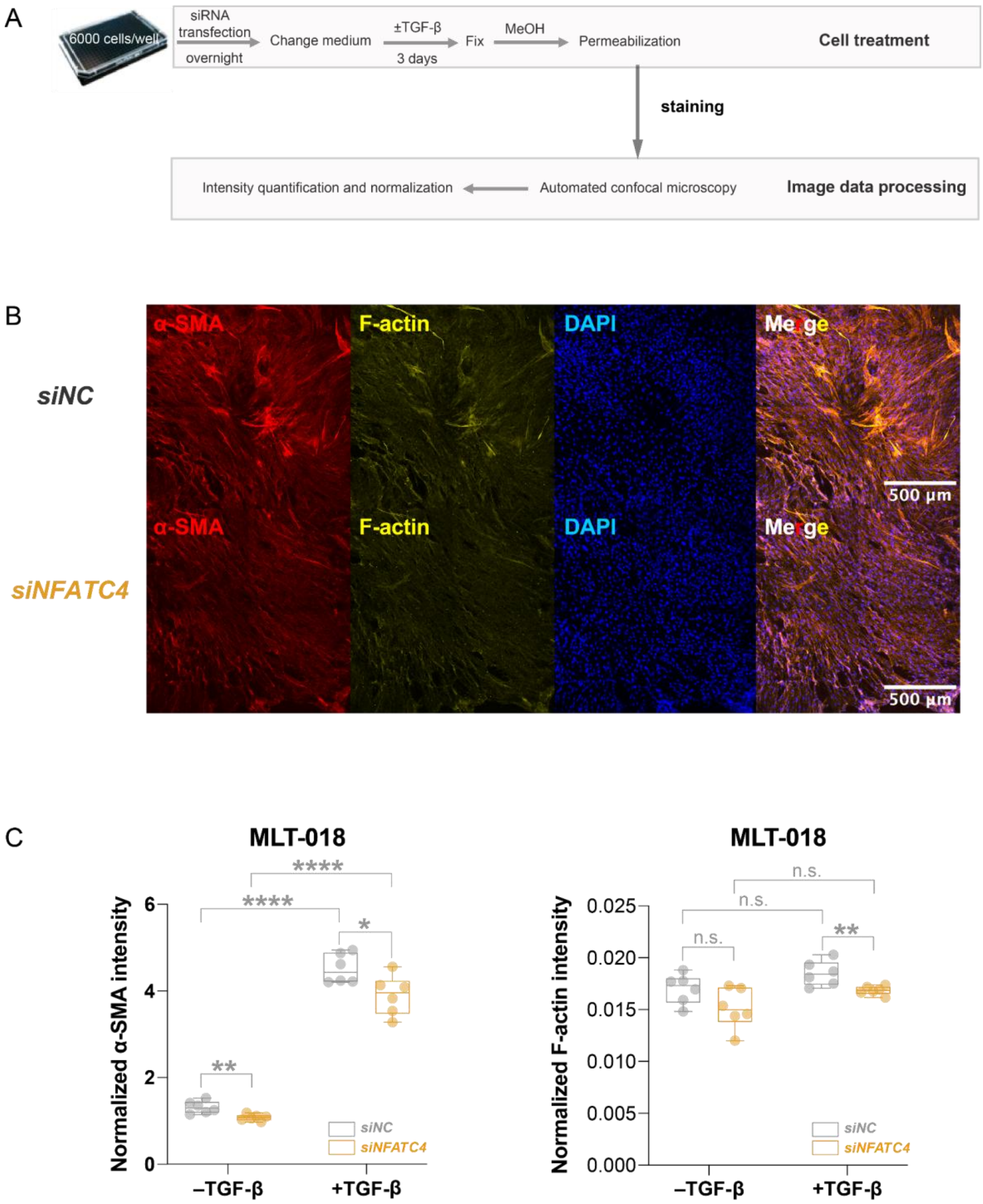

Figure S11. NFATC4 knockdown reduced ACTA2 and stress fiber. (A) Flowchart of actin staining. Fibroblasts were seeded in a Fibronectin-coated 384-well CellCarrier plate, transfected with siRNAs, treated with TGF-β (1 ng/mL in medium containing 1% FBS) for 3 days after starvation. Fibroblasts were then fixed with MeOH and stained. Confocal images were acquired, and maximum intensity projection (MIP) images were generated and processed. **(B)** Representative images showing the expression of alpha-smooth muscle actin (ACTA2) and filamentous actin (F-actin) in MLT-018 cells without TGF-β treatment. Scale bars, 500 μm. **(C)** Quantification of expression levels of ACTA2 and F-actin (fluorescence intensity normalized to Hoechst 33342 intensity) in MLT-018 cells, two-tailed Student's t-test for each pairwise comparison (n=6). Statistics: *p < 0.05, **p < 0.01, ***p < 0.001, ****p < 0.0001, and n.s.: not significant according to the t-test.

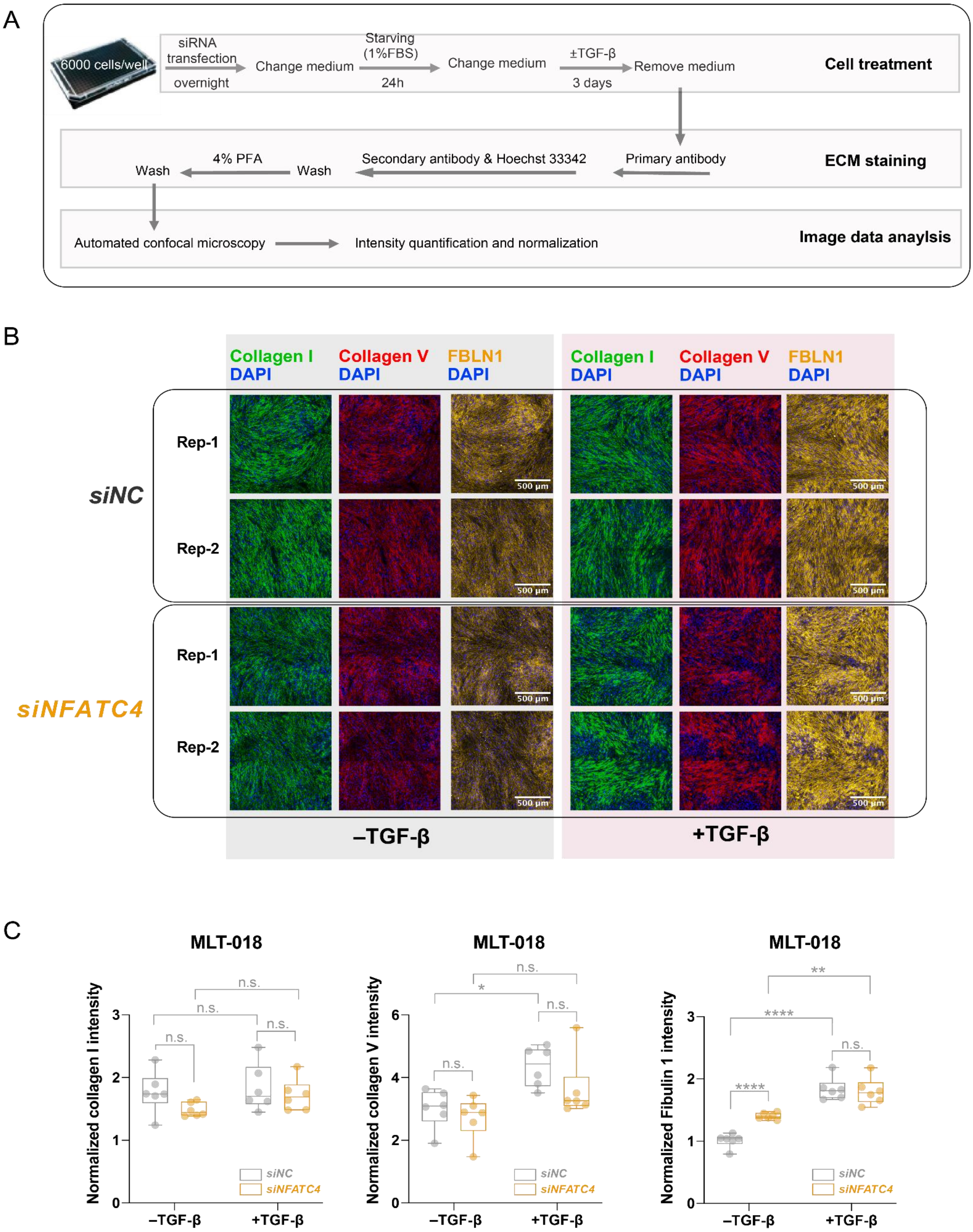

Figure S12. ***NFATC4*** **knockdown reduced ECM deposition. (A)** Flowchart of extracellular matrix (ECM) deposition assay. Fibroblasts were seeded in an Fibronectin-coated 384-well CellCarrier plate, transfected with siRNAs, and treated with TGF-β (1 ng/mL in medium containing 1% FBS) for 3 days after 24h starvation. Fibroblasts were then stained for ECM proteins. Confocal images were acquired, and maximum intensity projection (MIP) images were generated and processed. **(B)** Representative images showing the expression of collagen I, collagen V and FBLN1 in CCL-151. Scale bars, 500 μm. Abbreviation: Rep, Replicate. **(C)** Quantification of expression levels of collagen I, collagen V and FBLN1 (fluorescence intensity normalized to Hoechst 33342 intensity), two-tailed Student's t-test for each pairwise comparison (n=6) .

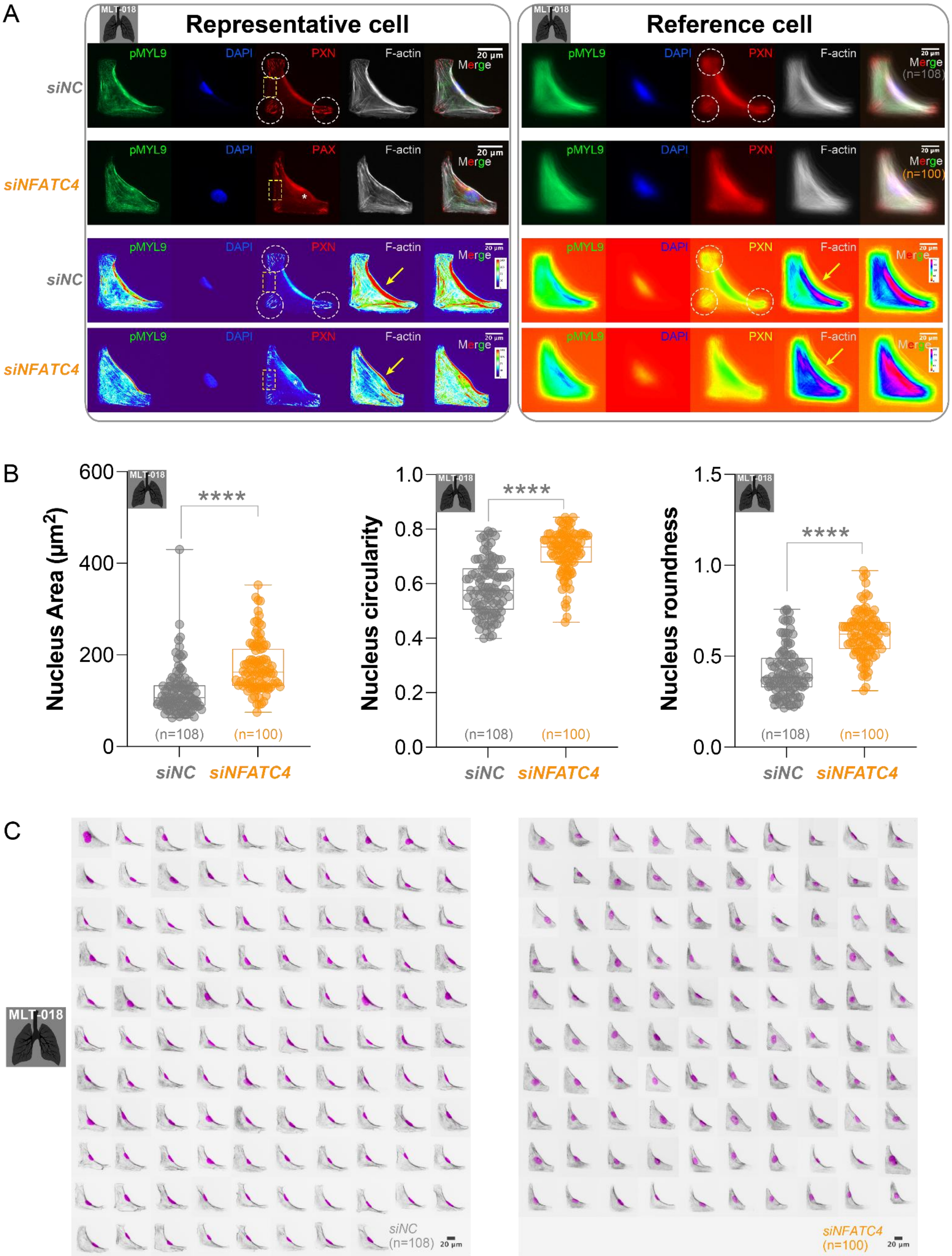

Figure S13. siNFATC4 affected cytoskeleton and nucleus phenotype. (A) MLT-018 cells on L-shaped micropattern were stained for pMYL9 (myosin activity marker, green), Paxillin (a component of focal adhesions, red), F-actin (stress fiber, gray), and nucleus (blue). A total of 108 *siNC*-transfected MLT-018 fibroblasts and 100 *siNFATC4*-transfected MLT-018 fibroblasts were analyzed. The Reference cell pictures (right panel) were generated using the average intensity (upper two rows). The fluorescent intensity of each channel was also illustrated by heatmaps (lower two rows). In *siNC*-transfected fibroblast, the focal adhesions indicated by Paxillin

preferentially enriched at the 3 points of an L-shape micropattern (white dashed circles), while in *siNFATC4*-transfected cells, the focal adhesions also distribute on the adhesive edges of the micropattern (yellow dashed box outline, left panel). In addition, a region of the lower expression level of Paxillin was shown (white asterisk); interestingly, we found a "paxillin outline" around the nucleus in most of the *siNFATC4*-transfected MLT-018, suggesting Paxillin is associated with nucleus shape, and NFATC4 may also regulate the perinuclear Paxillin. However, this paxillin outline is not obvious in *siNFATC4*-transfected CCL-151 fibroblasts. The distribution of F-actin also shows a more relaxed cytoskeleton architecture in *siNFATC4*-transfected fibroblasts. In *siNC*-transfected cells, F-actin bundles are highly stretched on the non-adhesive edge of micropatterns (lower two rows, yellow arrows), which is the contraction edge, suggesting involvement in cell contraction. In contrast, these fibers are more evenly distributed in *siNFATC4*-transfected fibroblasts. **(B)** Quantification of MLT-018 nucleus area, circularity, and roundness. **(C)** The shape and position of MLT-018 nuclei. Activated myosin was indicated by pMYL9 (gray), and the nucleus was indicated by DAPI (magenta). Statistics were calculated using a paired two-tailed Student's t-test on per-run means (n=3). Data are presented as mean ± SD; *P < 0.05, ***P < 0.001.

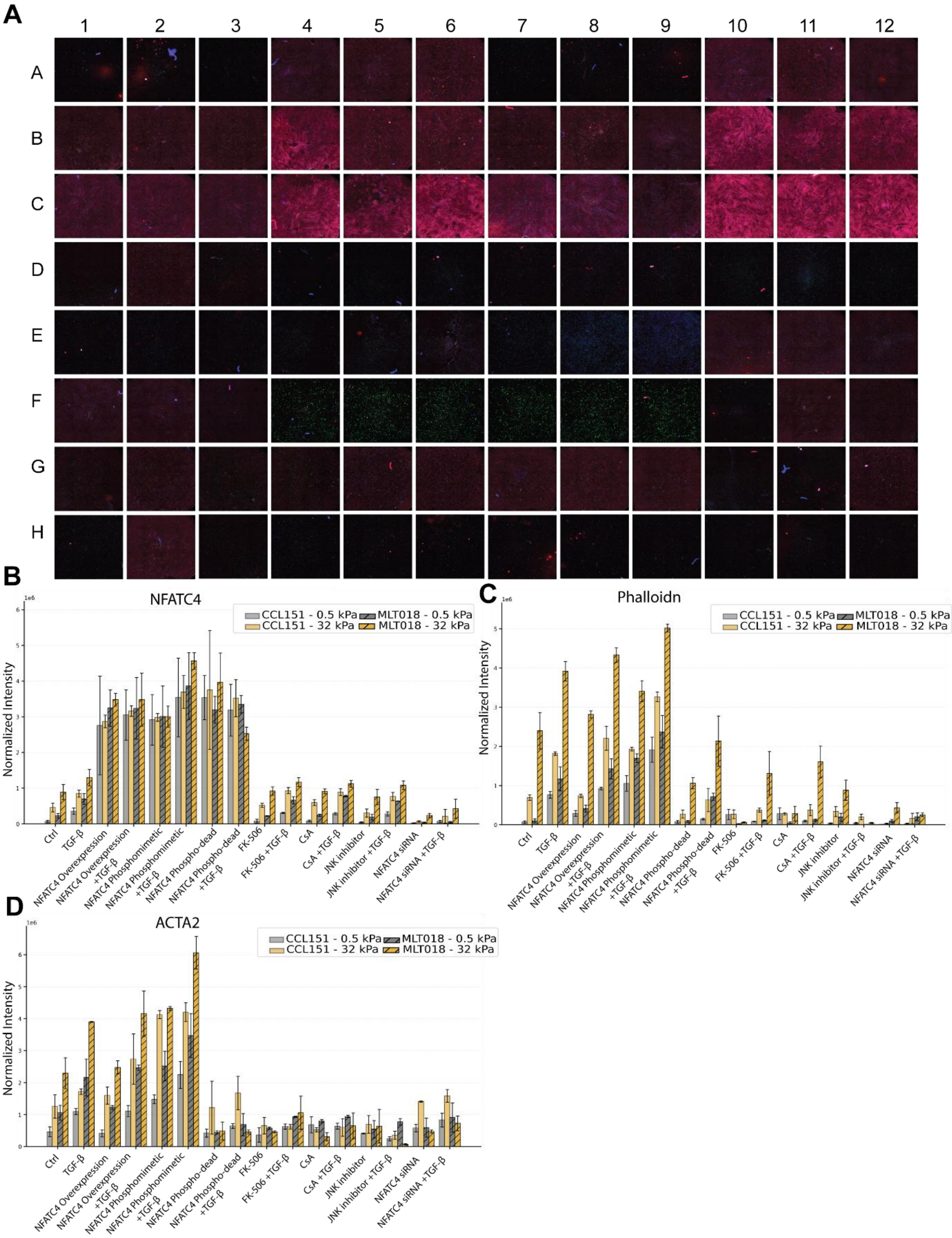

Figure S14. NFATC4 phosphorylation is required for myofibroblast differentiation downstream of mechanical stiffness and TGF-β. (A) Representative immunofluorescence images from a 96-well plate screen showing ACTA2 (red), NFATC4 (green), and DAPI (blue) staining in CCL-151 and MLT-018 fibroblasts across all experimental conditions (rows A–H, columns 1–12) on soft (0.5 kPa) and stiff (32 kPa) substrates, with and without TGF-β stimulation. Conditions include overexpression of wildtype NFATC4, phosphomimetic (S213D/S217D) and phospho-dead (S213A/S217A) mutants, pharmacological inhibition of NFAT activity (FK-506, CsA), JNK inhibition, and NFATC4 siRNA knockdown (see Table S3 for full condition layout). (B–D) Quantification of normalized fluorescence intensities for NFATC4 (B), F-actin (Phalloidin) (C), and ACTA2 (D) across all conditions in CCL-151 and MLT-018 cells on 0.5 kPa and 32 kPa substrates. Bars represent mean ±

SEM. CCL-151 results are shown in solid bars; MLT-018 in hatched bars. Gray: 0.5 kPa; gold: 32 kPa. Statistics are included in Table S5.

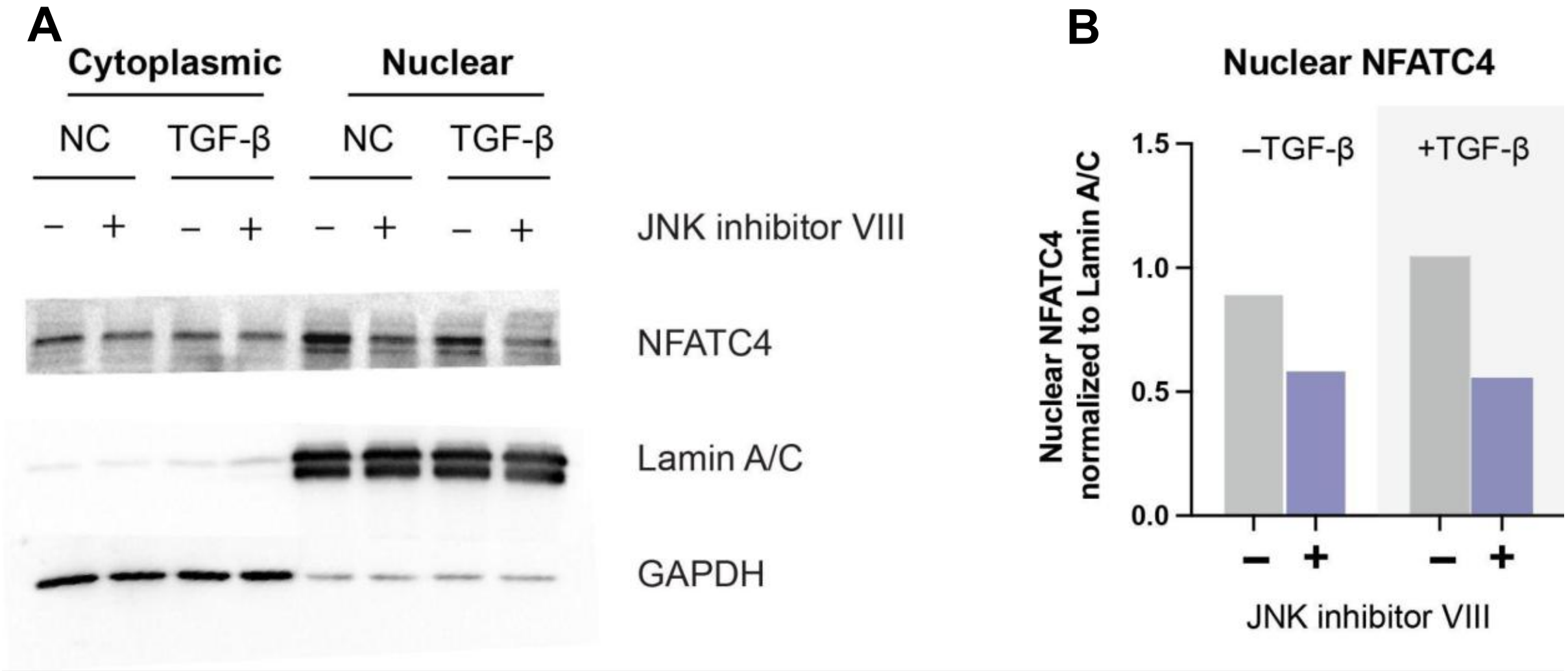

Figure S15. JNK inhibition suppresses nuclear NFATC4 translocation. (A) WB analysis of NFATC4 protein distribution in CCL-151 cells with/without TGF-β treatment. **(B)** Quantitative analysis of nuclear NFATC4 bands in A.

Supplementary Tables

Table S1:
Phospho quantification results from CCL-151 cells on substrates with different stiffness.

Table S2:
Phospho quantification results from the CCL-151 cells spreading time course.

Table S3:
Experimental conditions for NFATC4 functional validation with CCL151 and fibrotic pHLF (MLT018).

Table S4:
Tables with information on used antibodies and primer pairs.

Table S5:
Statistical analysis of ACTA2, Phalloidin, and NFATC4 immunofluorescence intensities in CCL151 and MLT018 fibroblasts across substrate stiffnesses, TGF-β stimulation, pharmacological treatments, and NFATC4 constructs.
