# Supplementary material for: Mechanosensitive phosphorylation of NFATC4 at S213/S217 drives fibroblast-to-myofibroblast transition and fibrosis
Source: J Clin Invest. 2026 Sep 1;136(17):e195121. doi: 10.1172/JCI195121 (PMC13528934; doi:10.1172/JCI195121)

Gels

ACTA2

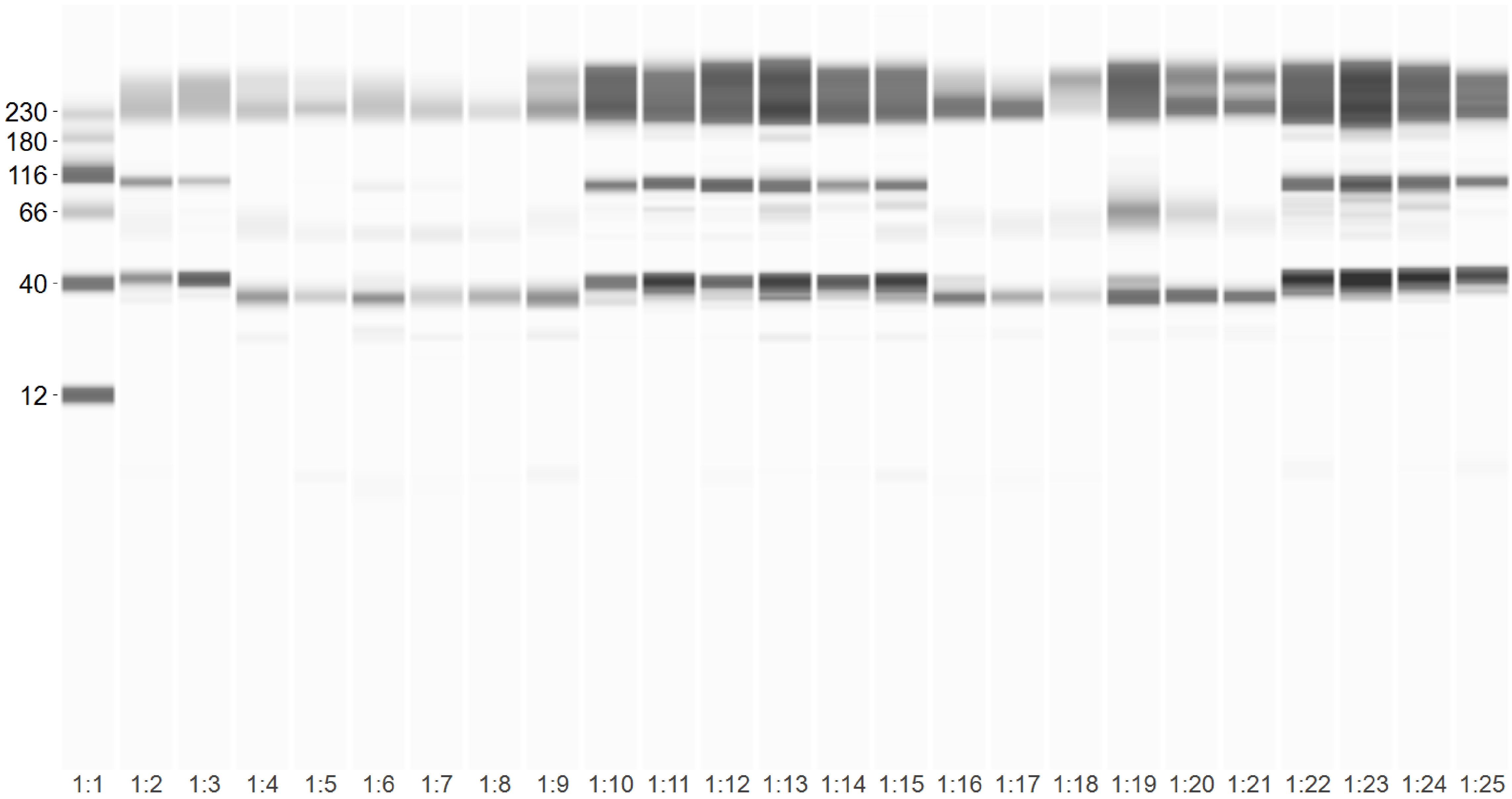

230
180
116
66
40
12
1:1
1:2
1:3
1:4
1:5
1:6
1:7
1:8
1:9
1:10
1:11
1:12
1:13
1:14
1:15
1:16
1:17
1:18
1:19
1:20
1:21
1:22
1:23
1:24
1:25

ACTA2

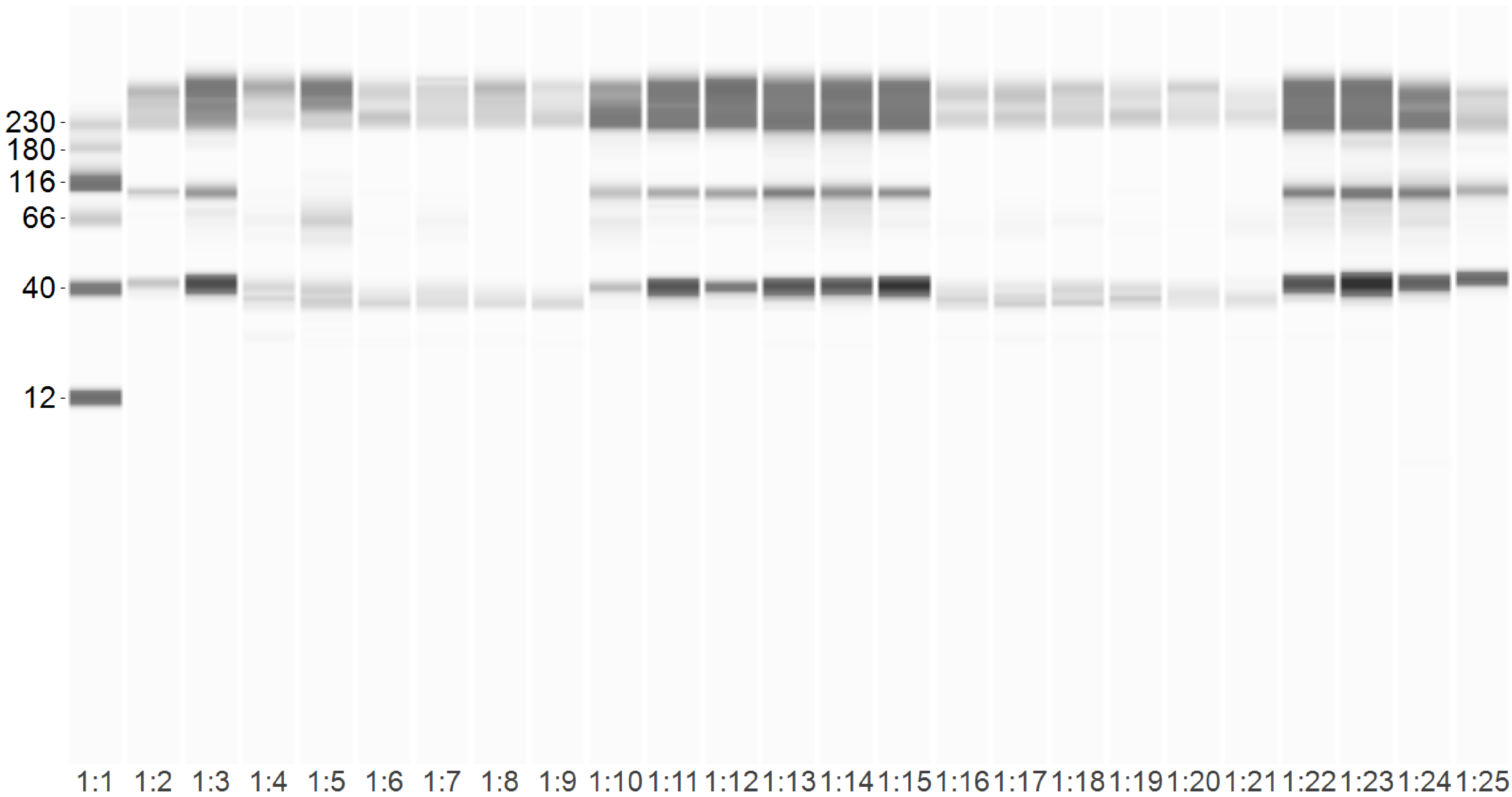
230
180
116
66
40
12
1:1 1:2 1:3 1:4 1:5 1:6 1:7 1:8 1:9 1:10 1:11 1:12 1:13 1:14 1:15 1:16 1:17 1:18 1:19 1:20 1:21 1:22 1:23 1:24 1:25

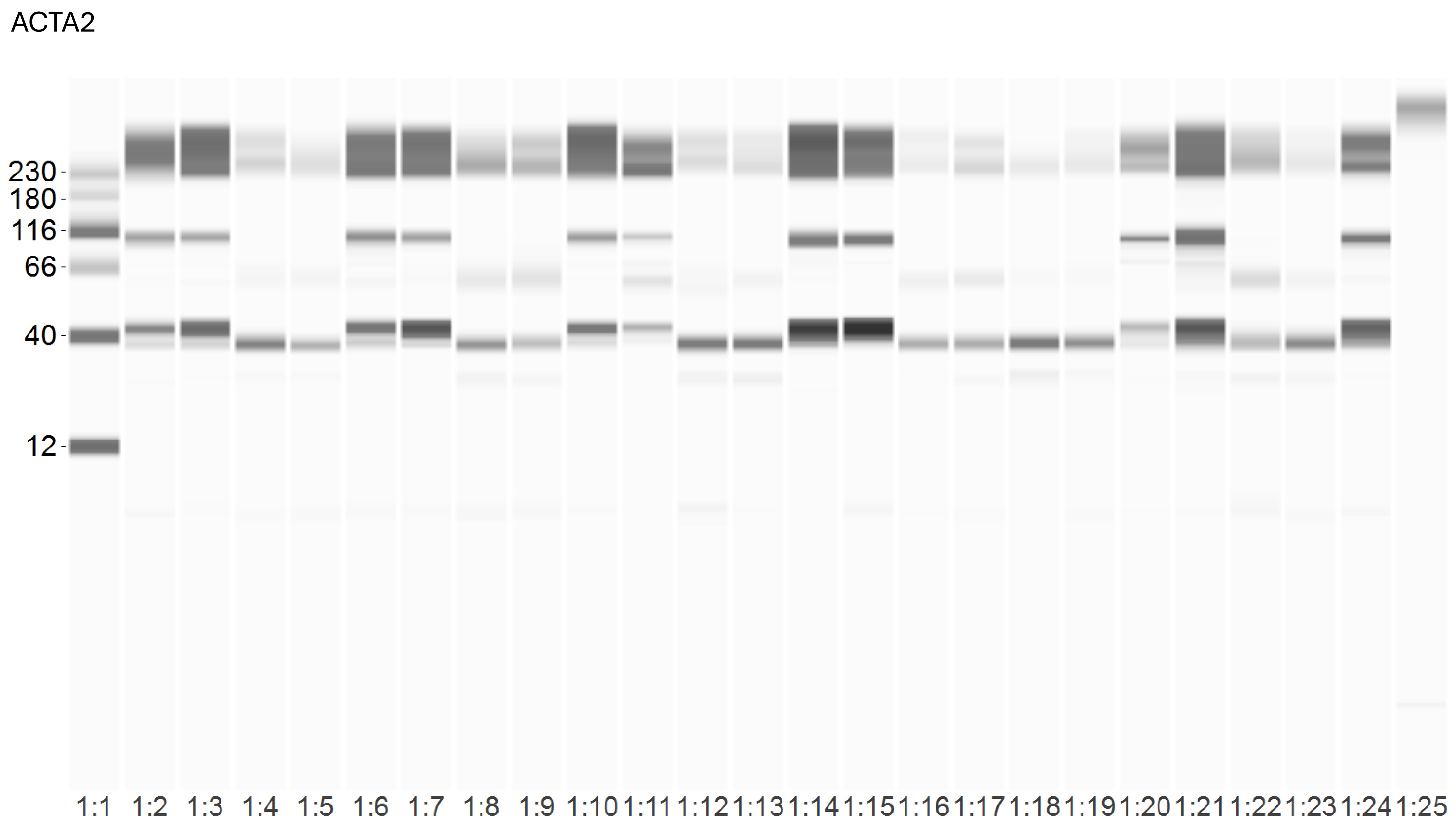
ACTA2
230
180
116
66
40
12
1:1
1:2
1:3
1:4
1:5
1:6
1:7
1:8
1:9
1:10
1:11
1:12
1:13
1:14
1:15
1:16
1:17
1:18
1:19
1:20
1:21
1:22
1:23
1:24
1:25

CTHRC1

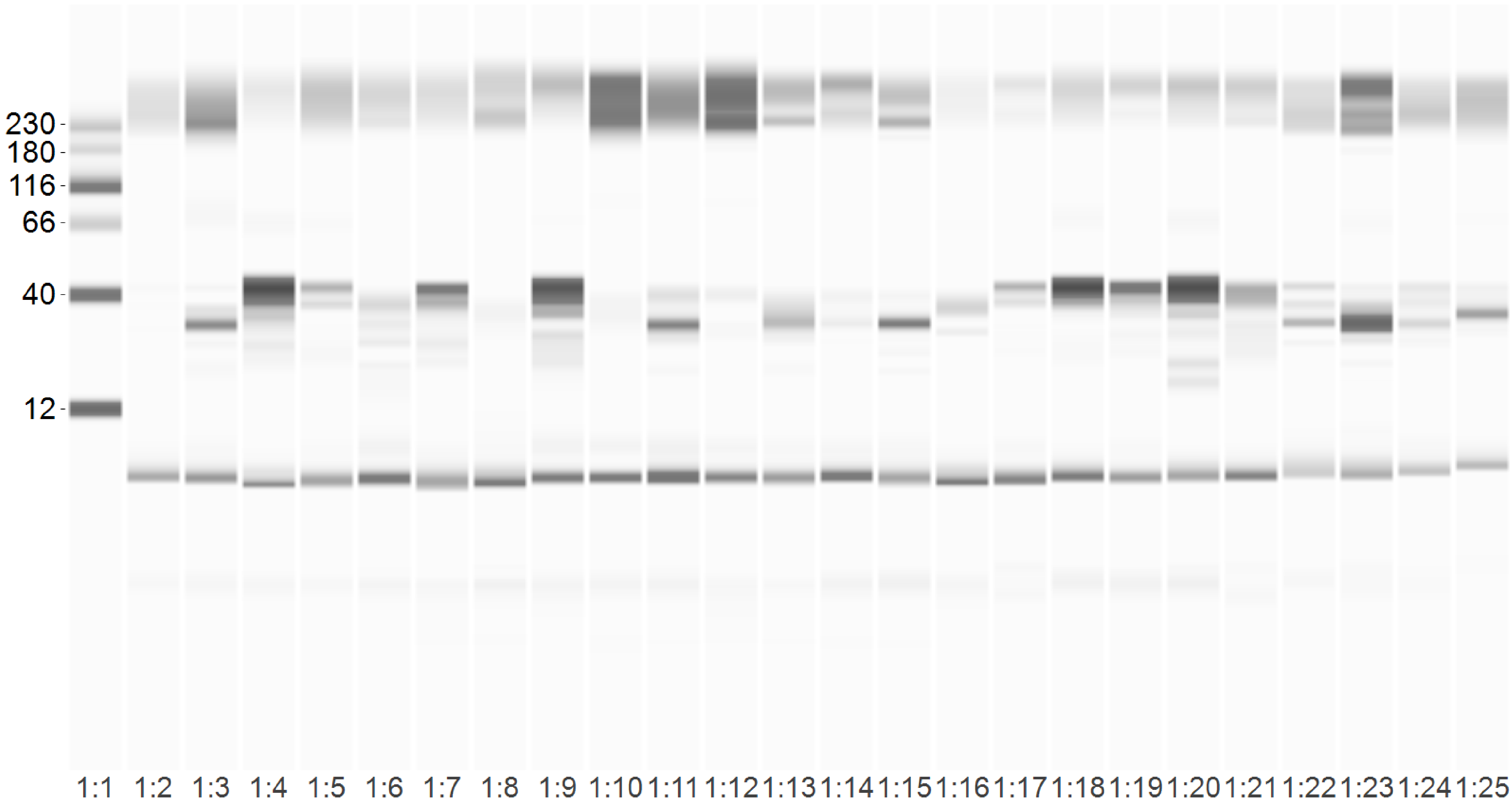

230
180
116
66
40
12
1:1 1:2 1:3 1:4 1:5 1:6 1:7 1:8 1:9 1:10 1:11 1:12 1:13 1:14 1:15 1:16 1:17 1:18 1:19 1:20 1:21 1:22 1:23 1:24 1:25

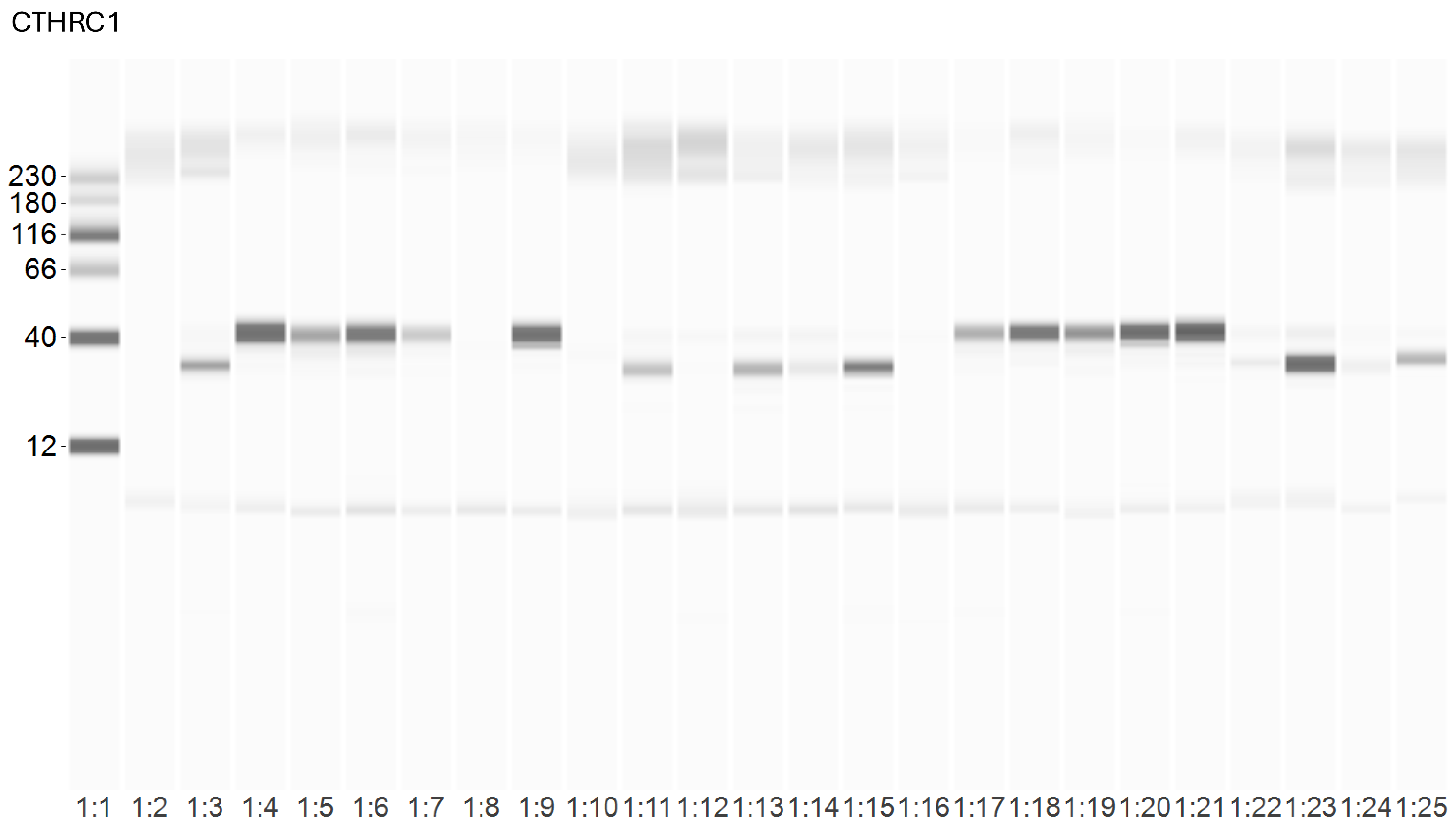
CTHRC1
230
180
116
66
40
12
1:1
1:2
1:3
1:4
1:5
1:6
1:7
1:8
1:9
1:10
1:11
1:12
1:13
1:14
1:15
1:16
1:17
1:18
1:19
1:20
1:21
1:22
1:23
1:24
1:25

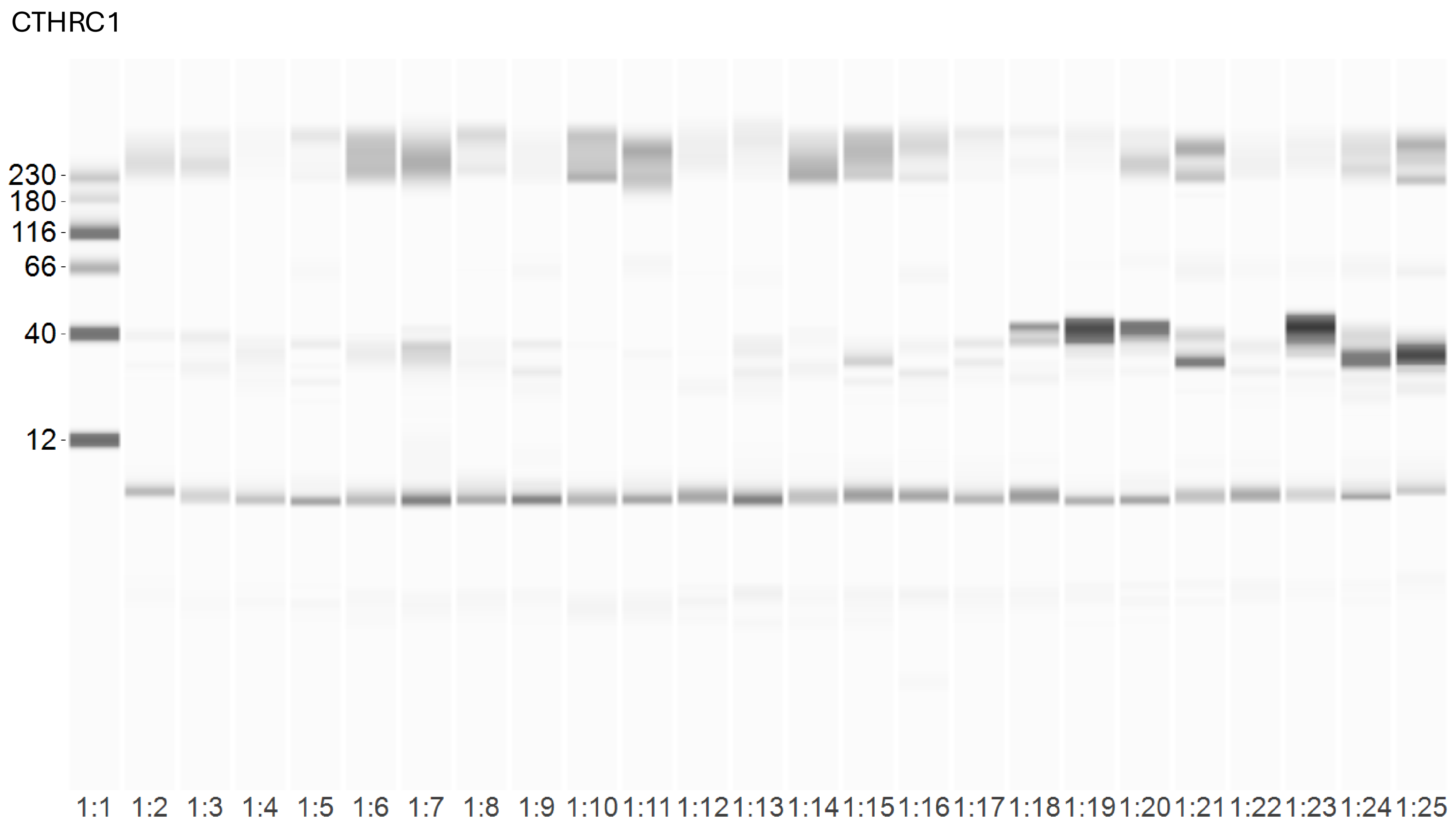
CTHRC1
230
180
116
66
40
12
1:1 1:2 1:3 1:4 1:5 1:6 1:7 1:8 1:9 1:10 1:11 1:12 1:13 1:14 1:15 1:16 1:17 1:18 1:19 1:20 1:21 1:22 1:23 1:24 1:25

NFATC4

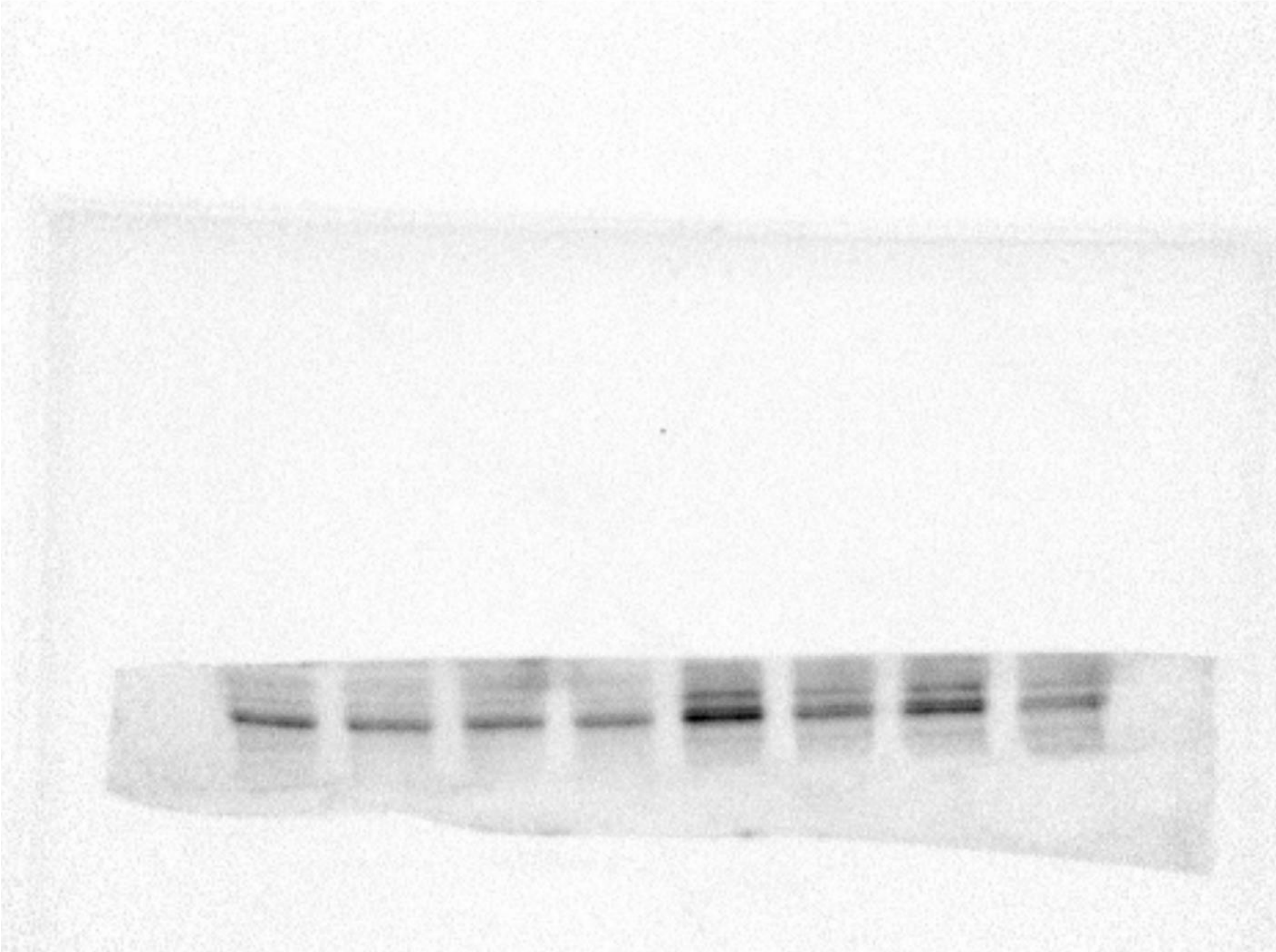

Lamin A/C

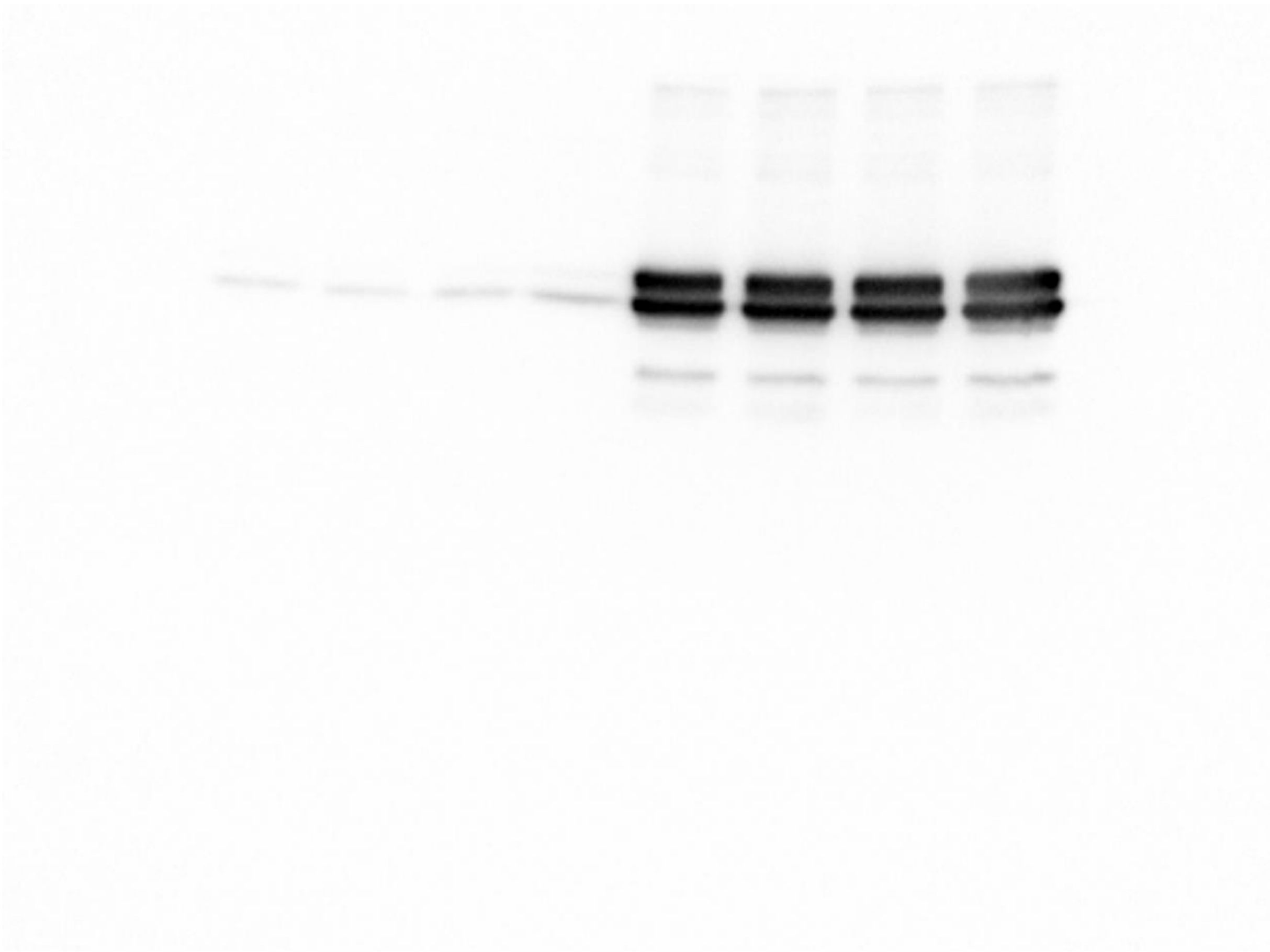

GAPDH

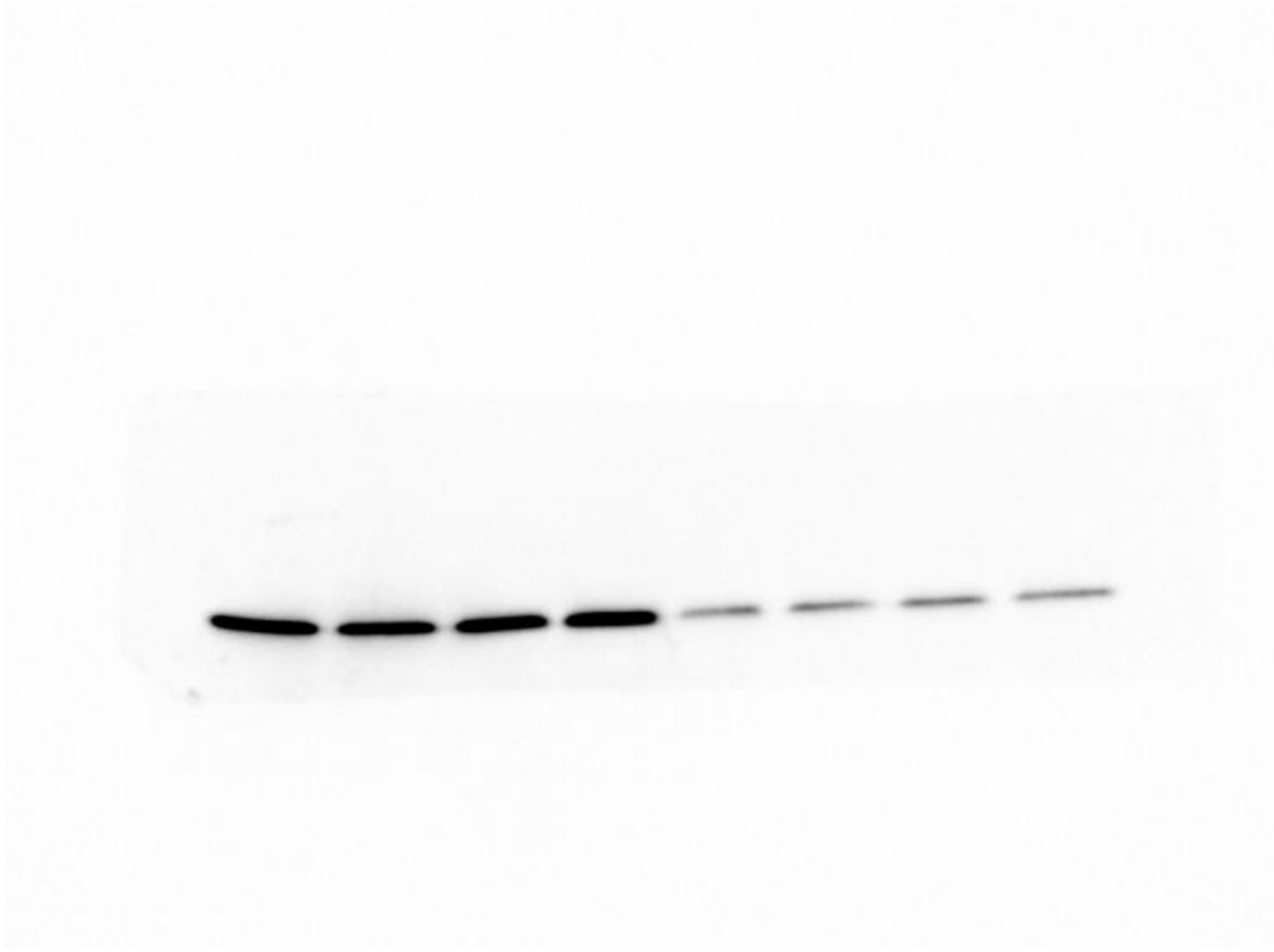

Supplement: Supplementary file 2 [file jci-136-195121-s117.pdf]
